# Creation of a Chiral All-Carbon Quaternary Center Induced by CF_3_ and CH_3_ Substituents via Cu-Catalyzed Asymmetric Conjugate Addition

**DOI:** 10.1021/acs.orglett.4c01691

**Published:** 2024-06-13

**Authors:** Taiyo Yamamoto, Masayuki Asakura, Ken Yamanomoto, Takanori Shibata, Kohei Endo

**Affiliations:** † Department of Chemistry, Faculty of Science, Tokyo University of Science, Shinjuku, Tokyo 162-8601, Japan; ‡ Department of Chemistry and Biochemistry, Graduate School of Science and Technology, Waseda University, Shinjuku, Tokyo 169-8555, Japan

## Abstract

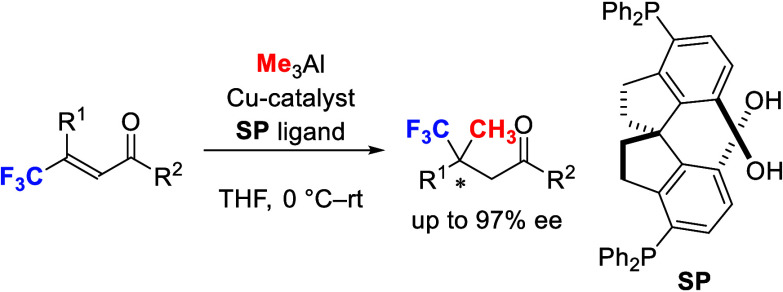

Cu-catalyzed asymmetric construction of a chiral quaternary center bearing CH_3_ and CF_3_ groups was achieved with high to excellent enantioselectivity using our originally developed ligands. The asymmetric conjugate addition of Me_3_Al to β-CF_3_-substituted enones and unsaturated ketoesters proceeded efficiently. The use of unsaturated ketoesters gives optically active furanones in high yields with high enantioselectivities. The perfluoroalkyl-substituted enone does not seem to be favorable in the present reaction.

The creation of fluorine- and fluorocarbon-bearing chiral carbons has recently attracted much attention in the area of pharmaceutical chemistry.^1^ There have been promising studies on the enantioselective introduction of fluorine atoms and a trifluoromethyl group (CF_3_), since natural products contain various C–H- and C–CH_3_-substituted achiral or chiral centers. Although the introduction of H and F at the same carbon atom has been reported extensively, there have been several reports on the introduction of the CH_3_ group and the CF_3_ group at the same carbon atom (Figure 1). While several biologically active compounds have fluorine- and fluorocarbon-bearing chiral carbons, to our knowledge there have been rare examples on the enantioselective construction of CH_3_ and CF_3_ group-induced all-carbon quaternary centers (Figure 2).^2,3^ For the preparation of a quaternary carbon bearing CH_3_ and CF_3_ groups, several approaches are available including nucleophilic methylation and electrophilic methylation (Figure 3). There have been a several examples of nucleophilic, electrophilic, and radical trifluoromethylations, which are not reactive enough for the construction of a chiral quaternary carbon.^4^ Therefore, the addition reaction to CF_3_-substituted compounds seems to be a favorable approach.^5^ Herein, we report a novel example of the enantioselective catalytic construction of a CH_3_- and CF_3_-substituted chiral quaternary carbon via the conjugate addition of a CH_3_-nucleophile to a CF_3_-substituted activated olefin.^6^

**Figure 1:**
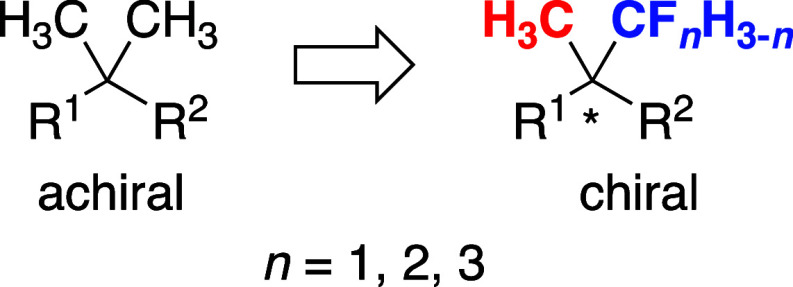
Fluorine-atom induced chirality.

**Figure 2:**
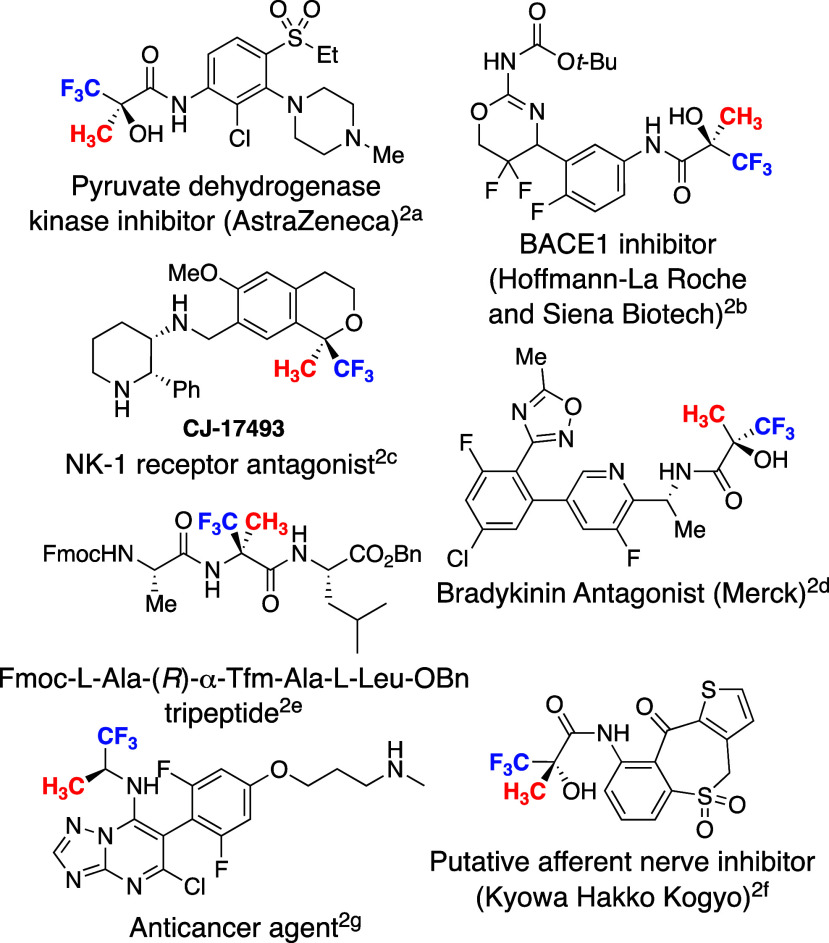
Examples of compounds bearing a CH_3_- and CF_3_-substituted chiral center.

**Figure 3:**
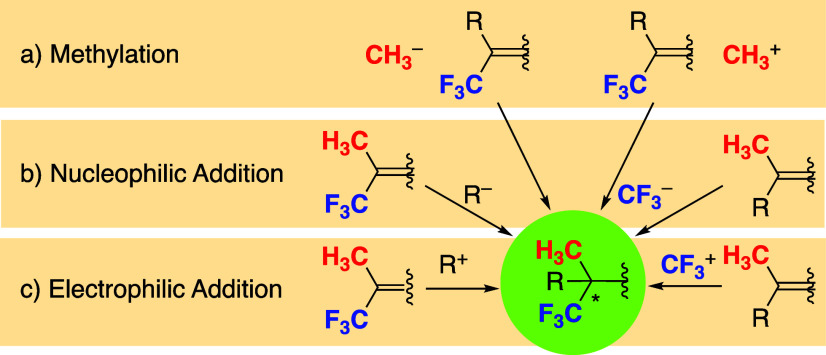
Approaches to CH_3_- and CF_3_-substituted all-carbon substituted chiral quaternary carbon.

To achieve the present strategy, we examined our previously developed multinuclear catalysts for the enantioselective conjugate addition of organoaluminum reagents to β-CF_3_-substituted α,β-unsaturated ketones (Table 1).^7^ The use of monophosphine ligand **BmP** (10 mol %) with CuCl_2_·2H_2_O (5 mol %) for the conjugate addition of Me_3_Al (3 equiv) to **1a** in THF at 0 °C to rt gave the desired product **2a** in good yield, but the enantioselectivity was moderate (entry 1). The use of bisphosphine **BP** (5 mol %) instead of **BmP** gave the product **2a** in good yield, but the enantioselectivity was poor (entry 2). Finally, the use of **SP** as a ligand gave product **2a** in moderate yield, but the enantioselectivity was high (entry 3). Therefore, we selected **SP** as a suitable ligand in the present reaction (Figure 4). The use of Cu(OAc)_2_ (5 mol %) instead of CuCl_2_·2H_2_O with **SP** (5 mol %) for the conjugate addition of Me_3_Al (3 equiv) to **1a** in THF at 0 °C to rt gave the product **2a** in improved yield, 85%, but the enantioselectivity dramatically decreased (entry 4). The use of CuI or Cu(BF_4_)_2_·6H_2_O as a Cu-source gave moderate results (entries 5 and 6). To our delight, the use of Cu(NO_3_)_2_·3H_2_O as a Cu-source showed high catalytic activity and enantioselectivity (entry 7).

**Figure 4:**
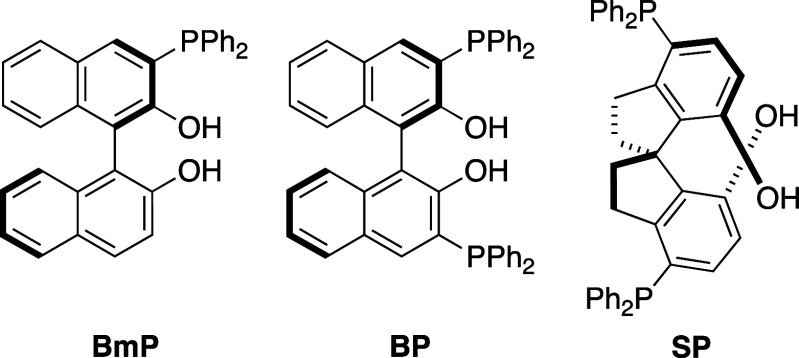
Representative ligands.

**Table 1:**
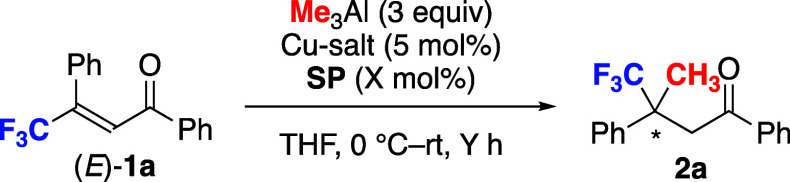
Screening of Ligands and Cu-Salts

entry	Cu-salt	ligand (*X*)	*Y*	yield, ee (%)^a^
1	CuCl_2_·2H_2_O	**BmP** (10)	48	82, 59 (−)
2	CuCl_2_·2H_2_O	**BP** (5)	168	81, 4 (−)
3	CuCl_2_·2H_2_O	**SP** (5)	30	62, 91 (+)
4	Cu(OAc)_2_	**SP** (5)	30	85, 15 (+)
5	CuI	**SP** (5)	48	64, 69 (+)
6	Cu(BF_4_)_2_·6H_2_O	**SP** (5)	30	82, 71 (+)
7	Cu(NO_3_)_2_·3H_2_O	**SP** (5)	30	93, 94 (+)

a The ee was determined by chiral HPLC analysis. The sign of optical rotation of the major enantiomer is given in parentheses.

The products are given in Figure 5. The reaction gave the *para*-substituted products **2b**, **2c**, **2d**, and **2e** in high yield with high enantioselectivity; electron-donating (-OMe in **2d**) and electron-withdrawing (-F in **2e**) groups are compatible with the reaction conditions. In contrast, the starting material was almost completely recovered for the *ortho*-tolyl derivative **1f**. The heteroaromatic derivatives **2g** and **2h** were obtained with high enantioselectivity, albeit in moderate yield. The relatively bulky 2-naphthyl group is compatible with the reaction, and product **2i** was obtained in moderate yield with high enantioselectivity.

**Figure 5:**
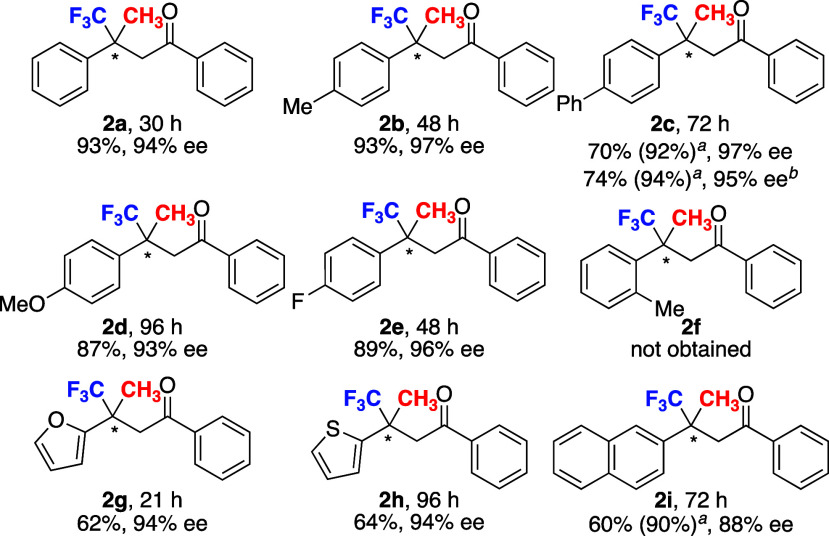
Examples of compounds bearing CH_3_- and CF_3_-substituted chiral centers. The reaction of enones (0.25 mmol) and Me_3_Al (3 equiv) was carried out in the presence of Cu(NO_3_)_2_·3H_2_O (5 mol %) and **SP** (5 mol %) in THF at 0 °C to rt. ^*a*^NMR yield is given in parentheses. ^*b*^**1c** (1 mmol) was used.

Unsaturated ketoesters were used for the synthesis of furanone derivatives via conjugate addition and subsequent intramolecular cyclization.^7a^ We have already reported the use of simple unsaturated ketoesters for the one-pot synthesis of furanones, which are promising synthetic intermediates in biologically active molecules. The optimized reaction conditions were applied to several unsaturated ketoesters (Figure 6; see the Supporting Information for screening of conditions). The products **4b**–**e** were obtained in high yield with high enantioselectivity. The Br-substituted **4e** gave a fine crystal, which showed an *R* enantiomer by X-ray crystallographic analysis. The cyclohexylcarbonyl derivative **4f** was obtained in moderate yield with high ee, but the acetyl derivative **4g** was not detected due to the high volatility. The use of Et_3_Al instead of Me_3_Al retarded the reaction and gave the desired product **4h** in moderate yield with good ee along with the generation of unidentified byproducts. The acyclic products **5a** and **5g**, not furanones, were obtained when the reaction was conducted below 0 °C (eq 1). The reaction of **3a** under low temperature gave the acyclic product **5a** in 66% yield with 92% ee and gave **4a** in 9% yield with 94% ee. The acyclic product **5g** derived from **3g** was obtained at −20 °C in a good yield with moderate enantioselectivity.
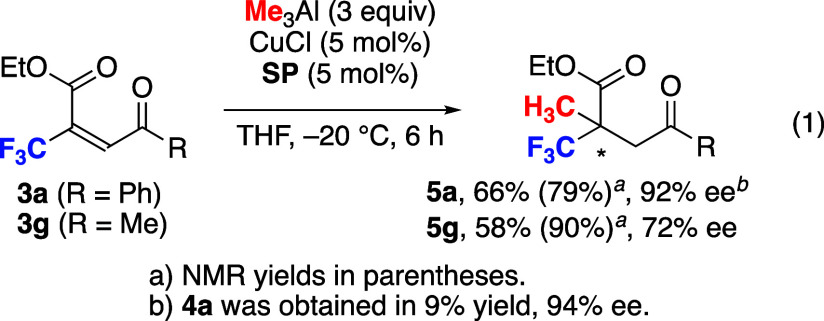
1
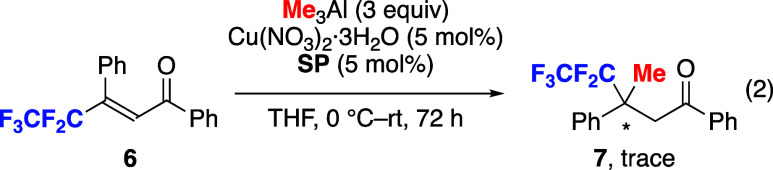
2

**Figure 6:**
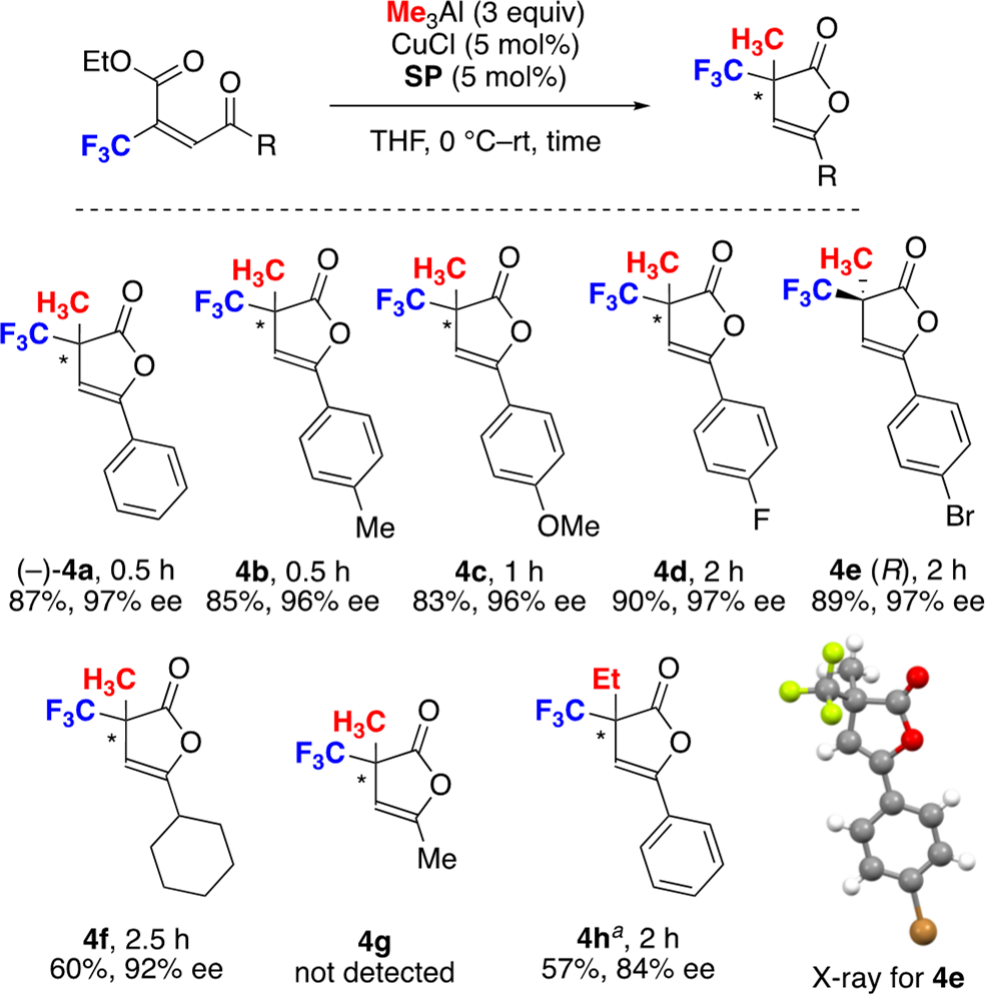
Examples of furanones. ^*a*^Et_3_Al (3 equiv) was used instead of Me_3_Al (3 equiv) for **4h**.

Other fluoroalkyl group was examined to understand the influence of the fluorine atom on the reactivity (eq 2). The reactivity of pentafluoroethyl-substituted enone **6** under the optimized conditions was very low, and only a trace amount of product was observed (not isolated). The present result indicates that the strong electron-withdrawing effect of a perfluoroalkyl substituent might not be suitable for use in the present Cu-catalysis, since a generated Cu^I^-π-complex would be a key intermediate for the insertion of a C–C double bond to a Cu–Me bond.^8,9^ The weak donating ability of a strongly electron-deficient olefin to a Cu-center might be disfavored in promoting the reaction via a π-complex for oxidative addition. There might be other factors of fluorine atoms retarding the reaction. We postulate a plausible stereoselectivity as shown in Figure 7. The reaction of CuX and Me_3_Al gives a CuMe-complex and Me_2_Al–X, where the Me_2_Al–X assisted con struction of the CuMe-enone complex promotes the methylation. The X-ray diffraction analysis of furanone **4e** indicates that the present stereoselectivity is the same as that in the reaction using typical trisubstituted enones in the presence of the same catalyst system.^7^ Based on the ESI-TOF-MS analyses of Cu-complexes derived from **SP** (see, the Supporting Information),^7d,7e^ Al-linked **SP**-Cu-complex is one of the possible structures for the effective stereoselectivity of conjugate addition (Figure 8). The result of the reaction using enone **1f** (R^1^ = Ph, R^2^ = *o*-tolyl) indicates that the steric hindrance around the carbonyl group of *s-cis* or *s-trans* enone might prevent the effective coordination to a Cu-center. The decrease of enantioselectivity using enone **3g** (R^1^ = Me, R^2^ = CO_2_Et) for **5g** might be attributed to the smaller steric repulsion in the *s-trans* enone. There might be π–π interaction between an enone and a Cu-complex for higher enantioselectivity. To elucidate the effect of olefinic geometry, (*Z*)-**1a** and (*Z*)-**3a** was used in the present reaction (eq 3). The opposite major enantiomer of **2a** and **4a** was observed as compared to that derived from (*E*)-**1a** and (*E*)-**3a**. Thus, the olefinic geometry of enones affects the stereoselectivity dramatically, as described in the transition state model. We previously reported that the Cu-catalyzed conjugate addition of Me_3_Al to enones and unsaturated ketoersters in the presence of **SP** as a ligand shows the same stereoselectivity.^7a,7d^ We consider that the same stereoselectivity works on the present conjugate addition to CF_3_-substituted enones **1** and unsaturated ketoesters **3** under the same catalyst system (see, the Supporting Information).
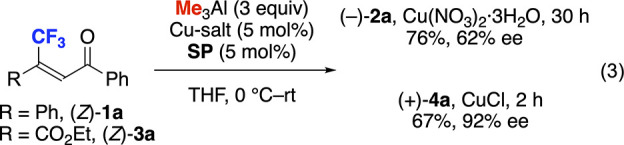
3

**Figure 7:**
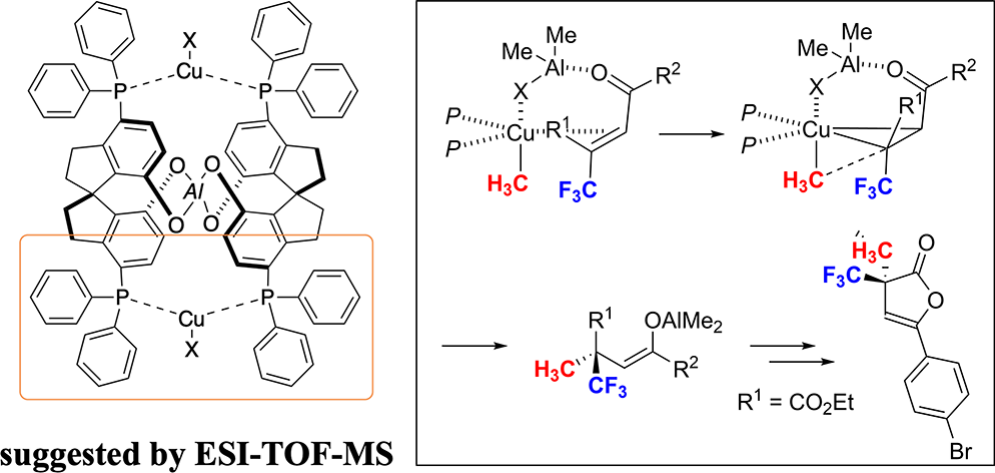
Plausible stereoselectivity.

**Figure 8:**
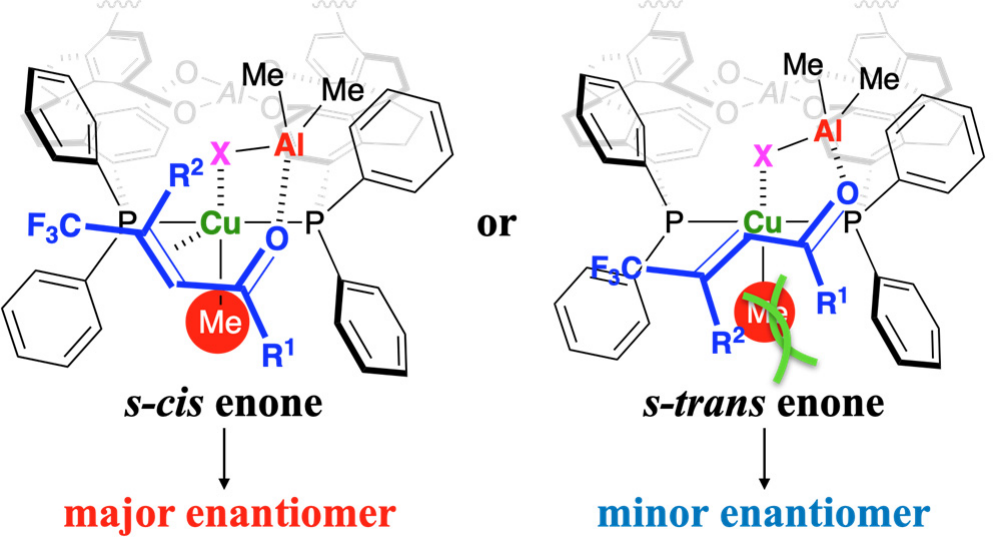
Postulated working model of the Cu complex with **SP**.

In conclusion, this report describes the highly enantioselective construction of CH_3_- and CF_3_-substituted chiral all-carbon quaternary carbon. A variety of enones possessing a CF_3_ group at the C–C double bond are tolerable under the reaction conditions and give the desired products in high enantioselectivity. The tandem conjugate addition/cyclization gives furanone derivatives in high yield with high enantioselectivity. The results of this study may encourage other researchers to develop methods for the construction of all-carbon chiral quaternary centers bearing CH_3_ and CF_3_ groups.

## Data Availability

The data underlying this study are available in the published article and the Supporting Information.

## Supporting Information Available

General information, synthesis and physical properties of new compounds, X-ray crystallographic data, NMR spectra for all compounds and HPLC spectra (PDF)
