# Supplementary material for: Creation of a Chiral All-Carbon Quaternary Center Induced by CF3 and CH3 Substituents via Cu-Catalyzed Asymmetric Conjugate Addition
Source: Org Lett. 2024 Jun 13;26(25):5312–7. doi: 10.1021/acs.orglett.4c01691 (PMC11217942; doi:10.1021/acs.orglett.4c01691)

Supporting Information

Creation of Chiral All-Carbon Quaternary Center Induced by CF_3 and CH_3 Substituents via Cu-Catalyzed Asymmetric Conjugate Addition

Taiyo Yamamoto[1], Masayuki Asakura[1], Ken Yamanomoto,[1] Takanori Shibata[2], Kohei Endo[1]*

[1] Department of Chemistry, Faculty of Science, Tokyo University of Science, Shinjuku, Tokyo 162-8601, Japan

[2] Department of Chemistry and Biochemistry, Graduate School of Science and Technology, Waseda University, Shinjuku, Tokyo, 169-8555, Japan

Contents:

**1. General Instruments and Chemicals**	**S2**
**2. Screening of Reaction Conditions and ESI-TOF-MS of Cu-complexes**	**S3**
**3. Synthesis and Physical Properties of New Compounds**	**S4**
**4. X-ray Single Crystal Data for 4e**	**S26**
**5. Prediction of Stereochemistry of Acyclic Product**	**S27**
**5. NMR Chart of New Compounds**	**S28**

1. General Instruments and Chemicals

1-1. General: All the reactions dealing with air or moisture sensitive compounds were carried out in a dry reaction vessel under positive pressure of argon. Air- and moisture-sensitive liquids and solutions were transferred via a syringe. Analytical thin layer chromatography was performed on a glass plates coated with 0.25 mm 230-400 mesh silica gel containing a fluorescent indicator (Merck, #1.05715.0009). Thin layer chromatography plates were visualized by exposure to ultraviolet light (254 nm) and/or by immersion in an acidic staining solution of p-anisaldehyde, molybdatophosphoric acid and potassium permanganate alkaline followed by heating. Organic solutions were concentrated by rotary evaporation. Flash column chromatography was performed on Kanto Silica gel 60 (spherical, neutral, 140–325 mesh).

1-2. Instrumentation: IR spectra were recorded with a spectrophotometer. NMR spectra were measured for ^{1}H, ^{13}C, and ^{19}F NMR using tetramethylsilane as an internal reference and trifluoroacetic acid as external references and $CDCl_3$ as a solvent. Chemical shift values for protons are reported in parts per million (ppm, δ scale) downfield from tetramethylsilane and are referenced to residual proton of $CDCl_3$ (δ 7.26). Carbon nuclear magnetic resonance spectra (^{13}C NMR) were recorded at 125 MHz: chemical shifts for carbons are reported in parts per million (ppm, δ scale) downfield from tetramethylsilane and are referenced to the carbon resonance of $CDCl_3$ (δ 77.0). Fluorine nuclear magnetic resonance spectra (^{19}F NMR) were recorded at 470 MHz or 375 MHz: chemical shifts for fluorine are reported in parts per million (ppm, δ scale) referenced to the fluorine resonance of trifluoroacetic acid (δ –76.5). Data are presented as following space: chemical shift, multiplicity (s = singlet, d = doublet, t = triplet, q = quartet, m = multiplet and/or multiplet resonances), coupling constant in hertz (Hz), and signal area integration in natural numbers, assignment (italic). Mass spectra were measured using ESI-MS or APCI-MS. HRMS were collected using TOF. The single crystal X-ray diffraction analysis was collected on Bruker D8 QUEST ECO using Mo Kα.

1-3. Chemicals: All reagents were purchased as commercially available source unless otherwise noted. (*R*)-BINOL (>99.0% ee) was purchased from Fuji Molecular Planning Co., Ltd. The enones (*E*)-**1a**, (*Z*)-**1a**, **1b**, **1d**, **1e**, **1g**, **1h**, **1i**, **3g** were prepared and characterized according to the literature.[1] **BP**, **B*m*P**, and **SP** were synthesized according to our previous reports.[2]

---

[1] (a) Kwiatkowski, P.; Cholewiak, A.; Kasztelan, A. *Org. Lett.* **2014**, *16*, 5930–5933. (b) L, Zhenhua.; Z, Zhihai.; Z, Guangyu.; Z, Yiqin.; Y. Lin.; G, Wen.; T, Lili.; T, Bo. *Org. Lett.* **2019**, *21*, 7324. (c) Fioravanti, S.; Colantoni, D.; Pellacani, L.; Tardella, P. A. *J. Org. Chem.* **2005**, *70*, 3296–3298.

[2] (a) Endo, K.; Ogawa, M.; Shibata, T. *Angew. Chem. Int. Ed.* **2010**, *49*, 2410–2413. (b) Endo, K. ; Tanaka, K.; Ogawa, M.; Shibata, T. *Org. Lett.* **2011**, *13*, 868. (c) Endo, K.; Hamada, D.; Yakeishi, S. ; Ogawa, M.; Shibata, T. *Org. Lett.* **2012**, *14*, 2342–2345. (d) Endo, K.; Hamada, D.; Yakeishi, S.; Shibata, T. *Angew. Chem. Int. Ed.* **2013**, *52*, 606–610.

2. Screening of Reaction Conditions and ESI-TOF-MS of Cu-complexes

· Table 1S. Screening of Cu-salts for unsaturated ketoester 3a

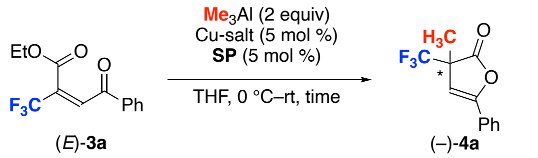

entry	Cu-salt	time	yield, ee (%)[a]
1	$Cu(NO_3)_2{\cdot}3H_2O$	30 min	49, 95
2	$CuCl_2{\cdot}2H_2O$	30 min	63, 90
3	$Cu(OTf)_2$	40 min	50, 95
4	$Cu(acac)_2$	60 min	33, 92
5	$Cu(OAc)_2{\cdot}H_2O$	40 min	34, 94
6	CuI	29 h	76, 91
7	CuCl	1 h	79, 95
8[b]	CuCl	30 min	87, 97

[a] Ee was determined by chiral HPLC analysis. [b] Me_3Al (3 equiv) was used.

· ESI-TOF-MS analysis of Cu and Al-complexes

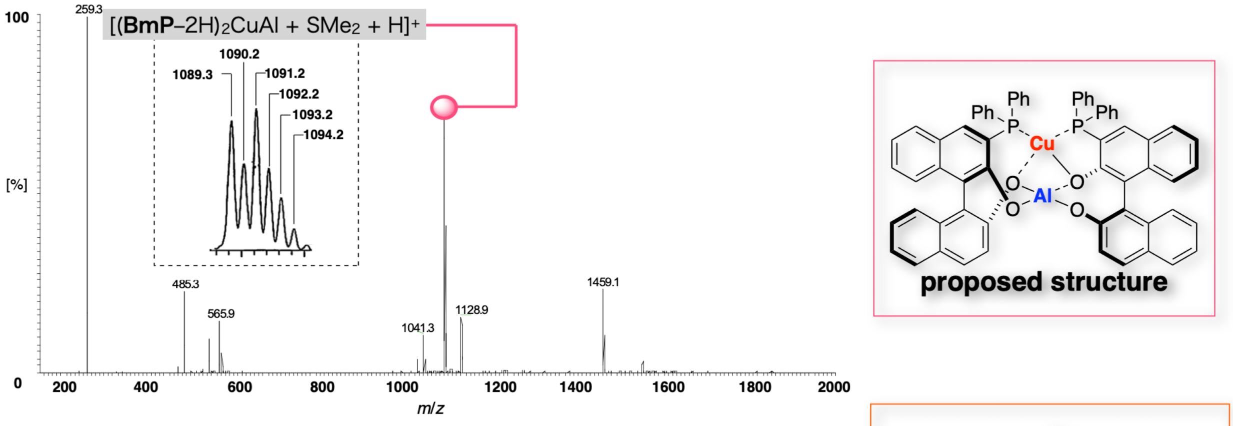

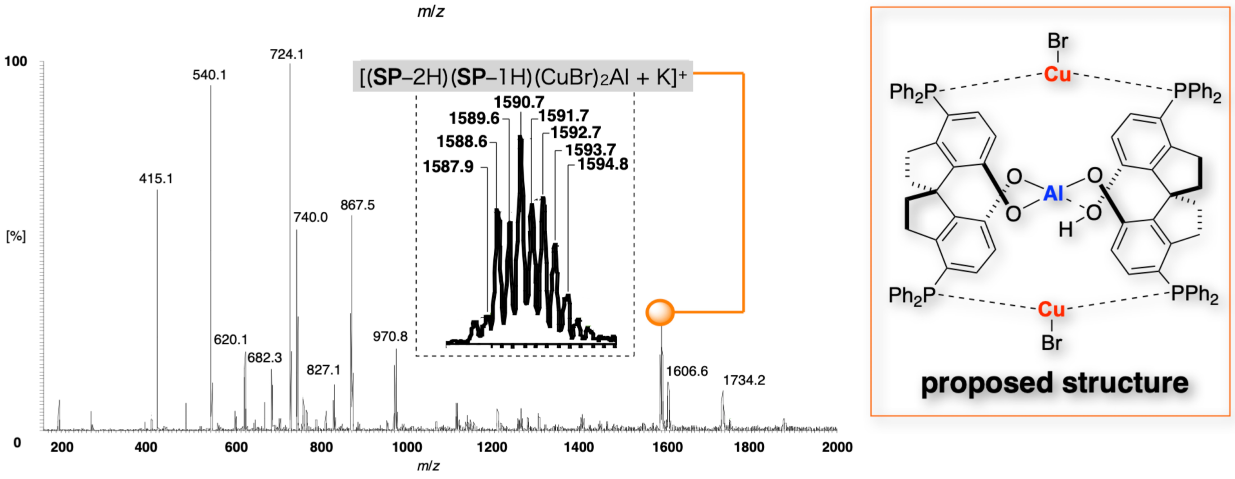

3. Synthesis and Physical Properties of New Compound
3-1. Substrate

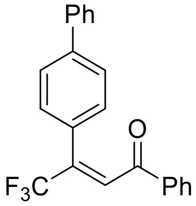

(*E*)-3-[1,1'-biphenyl]-4-yl-4,4,4-trifluoro-1-phenyl-2-buten-1-one (1c): To the solution of 1-[1,1′-Biphenyl]-4-yl-2,2,2-trifluoro-1-ethanone (5.0 mmol, 1.25 g) in toluene (10 mL), 1-phenyl-2-(triphenylphosphoranylidene)ethanone (6.0 mmol, 2.28 g) was added. The mixture was stirred under reflux condition for 24 hours. Then the mixture was concentrated and filtered through silica gel pad and washed with hexane/EtOAc (10/1) to remove triphenylphosphine oxide. The eluent was concentrated and purified by silica gel chromatography (hexane/EtOAc = 20/1) to afford **1c** (2.5 mmol, 0.90 g, 51%): white solid; mp 96 °C; ^{1}H NMR (400 MHz, $CDCl_3$) δ 7.88–7.82 (m, 2H), 7.56–7.45 (m, 5H), 7.44–7.37 (m, 4H), 7.37–7.28 (m, 4H); ^{13}C{^{1}H} NMR (100 MHz, $CDCl_3$) δ 192.1, 142.2, 140.0, 138.7 (q, J = 31.0 Hz), 136.1, 133.9, 130.8 (q, J = 5.1 Hz), 129.7, 129.5, 128.9, 128.8, 128.7, 127.7, 127.1, 127.0, 122.88 (q, J = 275.0 Hz); ^{19}F NMR (375 MHz) δ –66.02; HRMS (APCI, positive) *m/z*: $[M + H]^+$ calcd for $C_{22}H_{16}F_3O^+$ 353.1148; found 353.1140.

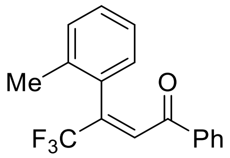

(*E*)-4,4,4-trifluoro-3-(2-methylphenyl)-1-phenyl-2-buten-1-one (1f): To the solution of 2,2,2-Trifluoro-1-(2-methylphenyl)-1-ethanone (3.3 mmol, 0.62 g) in toluene (10 mL), 1-phenyl-2-(triphenylphosphoranylidene)ethanone (3.3 mmol, 1.26 g) was added. The mixture was stirred under reflux condition for 40 hours. Then the mixture was concentrated and filtered through silica gel pad and washed with hexane/EtOAc (10/1) to remove triphenylphosphine oxide. The eluent was concentrated and purified by silica gel chromatography (hexane/EtOAc = 20/1) to **1f** (1.2 mmol, 0.34 g, 23%): yellow oil; ^{1}H NMR (400 MHz, $CDCl_3$) δ 7.84 (d, J = 7.7 Hz, 2H), 7.54 (t, J = 7.7 Hz, 1H), 7.49 (s, 1H), 7.42 (t, J = 7.7 Hz), 7.26-7.05 (m, 4H), 2.26 (s, 3H); ^{13}C{^{1}H} NMR (100 MHz, $CDCl_3$) δ 190.2, 139.6 (q, J = 31.2 Hz), 137.3, 136.4, 133.8, 130.2 (q, J = 5.8 Hz), 130.1, 129.1, 129.0, 128.7, 128.6, 125.3, 122.8 (q, J = 274.9 Hz), 19.8 (One carbon atom was not found probably due to overlapping); ^{19}F NMR (375 MHz, $CDCl_3$) δ –67.52; HRMS (APCI, positive) *m/z*: $[M + H]^+$ calcd for $C_{17}H_{14}F_3O^+$ 291.0991; found 291.0997.

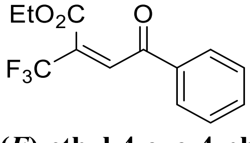

(*E*)-ethyl-4-oxo-4-phenyl-2-(trifluoromethyl)but-2-enoate ((*E*)-**3a**): To the solution of ethyl trifluoropyruvate (10 mmol, 1.3 mL, d = 1.28), 1-phenyl-2-(triphenylphosphoranylidene)ethanone (12 mmol, 4.57 g) was added. The mixture was stirred under reflux condition for 4 hours. Then the mixture was concentrated and filtered through silica gel pad and washed with hexane/EtOAc (10/1) to remove triphenylphosphine oxide. The eluent was concentrated and purified by silica gel chromatography (hexane/EtOAc = 10/1) to afford (*E*)-**3a** (6.98 mmol, 1.90 g, 70%): colorless oil; ^{1}H NMR (500 MHz, $CDCl_3$) δ 7.91–7.89 (m, 2H), 7.67–7.63 (m, 1H), 7.54–7.51 (m, 2H), 7.44 (q, J = 1.4 Hz, 1H), 4.13 (q, J = 7.2 Hz, 2H), 1.07 (t, J = 7.2 Hz, 3H); ^{13}C{^{1}H} NMR (125 MHz, $CDCl_3$) δ 190.8, 160.5, 141.2 (q, J = 4.8 Hz), 135.0, 134.4, 129.0, 128.7, 121.0 (q, J = 273.9 Hz), 62.5, 13.4; ^{19}F NMR (375 MHz, $CDCl_3$) δ –66.10; HRMS (APCI, positive) *m/z*: $[M + H]^+$ calcd for $C_{13}H_{12}F_3O_3^+$ 273.0733; found 273.0735.

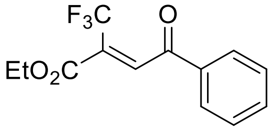

(*Z*)-ethyl-4-oxo-4-phenyl-2-(trifluoromethyl)but-2-enoate ((*Z*)-**3a**): The product was obtained from synthesis of (*E*)-**3a** as a minor product (1.42 mmol, 385.6 mg, 14%): pale yellow oil; ^{1}H NMR (400 MHz, $CDCl_3$) δ 7.89 (d, *J* = 7.5 Hz, 2H), 7.81 (s, 1H), 7.65 (t, *J* = 7.5 Hz, 1H), 7.52 (t, *J* = 7.5 Hz, 2H), 4.39 (q, *J* = 7.2 Hz, 2H), 1.38 (t, J = 7.2 Hz, 3H); $^{13}C\{^1H\}$ NMR (100 MHz, $CDCl_3$) δ 191.2, 161.1, 144.7 (q, *J* = 2.9 Hz), 134.7, 134.4, 129.0, 129.0, 126.8 (q, *J* = 32.7 Hz), 120.9 (q, *J* = 274.6 Hz), 62.6, 13.9; ^{19}F NMR (375 MHz, $CDCl_3$) δ -60.3; HRMS (APCI, positive) *m/z*: [M + H]$^+$ calcd for $C_{13}H_{12}F_3O_3^+$ 273.0733; found 273.0736.

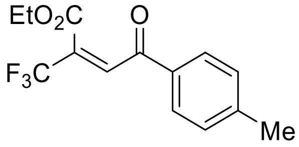

(*E*)-ethyl-4-oxo-4-(*p*-tolyl)-2-(trifluoromethyl)but-2-enoate (**3b**); To the solution of ethyl trifluoropyruvate (5.0 mmol, 0.66 mL, d = 1.28), 1-(4-methylphenyl)-2-(triphenylphosphoranylidene)ethanone (6.0 mmol, 2.37 g) was added. The mixture was stirred under reflux condition for 4 hours. Then the mixture was concentrated and filtered through silica gel pad and washed with hexane/EtOAc (10/1) to remove triphenylphosphine oxide. The eluent was concentrated and purified by silica gel chromatography (hexane/EtOAc = 50/1) to afford **3b** (1.8 mmol, 0.52 g, 35%): colorless oil; ^{1}H NMR (500 MHz, $CDCl_3$) δ 7.79 (d, *J* = 8.2 Hz, 2H), 7.42 (d, *J* = 1.3 Hz, 1H), 7.31 (d, *J* = 8.2 Hz, 2H), 4.13 (q, *J* = 7.1 Hz, 2H), 2.44 (s, 3H), 1.07 (t, *J* = 7.1 Hz, 3H); $^{13}C\{^1H\}$ NMR (125 MHz, $CDCl_3$) δ 190.3, 160.6, 145.6, 141.3 (d, *J* = 4.6 Hz), 132.5, 129.7, 128.8, 128.6 (q, *J* = 32.3 Hz) 121.1 (q, *J* = 273.7 Hz), 62.4, 21.8, 13.4; ^{19}F NMR (375 MHz, $CDCl_3$) δ –65.84; HRMS (ESI, positive) *m/z*: [M + Na]$^+$ calcd for $C_{14}H_{13}F_3O_3Na^+$ 309.0709; found 309.0723.

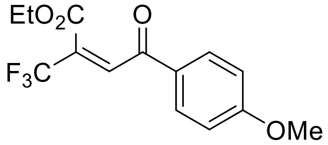

(*E*)-ethyl-4-(4-methoxyphenyl)-4-oxo-2-(trifluoromethyl)but-2-enoate (**3c**): To the solution of ethyl trifluoropyruvate (5.0 mmol, 0.66 mL, *d* = 1.28), 1-(4-methoxyphenyl)-2-(triphenylphosphoranylidene) ethanone (6.0 mmol, 2.46 g) was added. The mixture was stirred under reflux condition for 4 hours. Then the mixture was concentrated and filtered through silica gel pad and washed with hexane/EtOAc (10/1) to remove triphenylphosphine oxide. The eluent was concentrated and purified by silica gel chromatography (hexane/EtOAc = 5/1) to afford **3c** (1.7 mmol, 0.52 g, 34%): colorless oil; ^{1}H NMR (500 MHz, $CDCl_3$) δ 7.87–7.84 (m, 2H), 7.78 (s, 1H), 7.00–6.97 (d, *J* = 9.2 Hz, 2H), 4.39 (q, *J* = 7.1 Hz, 2H), 3.90 (s, 3H), 1.39 (t, *J* = 7.1 Hz, 3H); $^{13}C\{^1H\}$ NMR (125 MHz, $CDCl_3$) δ 189.1, 164.5, 160.7, 141.2-141.0 (m), 131.1, 128.4 (q, *J* = 32.2 Hz), 128.1, 121.1 (q, *J* = 273.7 Hz), 114.2, 62.3, 55.6, 13.4; ^{19}F NMR (375 MHz, $CDCl_3$) δ –66.03; HRMS (ESI, positive) *m/z*: [M + Na]$^+$ calcd for $C_{14}H_{13}F_3O_4Na^+$ 325.0658; found 325.0664.

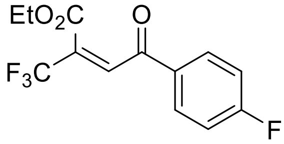

(*E*)-ethyl-4-(4-fluorophenyl)-4-oxo-2-(trifluoromethyl)but-2-enoate (**3d**): To the solution of ethyl trifluoropyruvate (5.0 mmol, 0.66 mL, *d* = 1.28), 1-(4-fluorophenyl)-2-(triphenylphosphoranylidene)ethanone (6.0 mmol, 2.39 g) was added. The mixture was stirred under reflux condition for 4 hours. Then the mixture was concentrated and filtered through silica gel pad and washed with hexane/EtOAc (10/1) to remove triphenylphosphine oxide. The eluent was concentrated and purified by silica gel chromatography (hexane/EtOAc = 10/1) to afford **3d** (2.3 mmol, 0.67 g, 44%): colorless oil; ^{1}H NMR (500 MHz, $CDCl_3$) δ 7.96–7.92 (m, 2H), 7.42 (d, *J* = 1.3 Hz, 1H), 7.22–7.18 (m, 2H), 4.15 (q, *J* = 7.2 Hz, 2H), 1.10 (t, *J* = 7.2 Hz, 3H); ^{13}C{^{1}H} NMR (125 MHz, $CDCl_3$) δ 189.3, 167.4, 165.4, 160.4, 141.1 (d, *J* = 3.5 Hz), 131.4 (d, *J* = 9.6 Hz), 128.9 (q, *J* = 32.4 Hz), 120.9 (q, *J* = 273.9 Hz), 116.3 (d, *J* = 21.5 Hz), 62.5, 13.4; ^{19}F NMR (375 MHz, $CDCl_3$) δ –66.07, –103.17; HRMS (ESI, positive) *m*/*z*: [M + Na]$^{+}$ calcd for $C_{13}H_{10}F_4O_3Na^{+}$ 313.0458; found 313.0467.

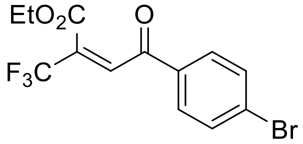

(*E*)-ethyl-4-(4-bromophenyl)-4-oxo-2-(trifluoromethyl)but-2-enoate (**3e**): To the solution of ethyl trifluoropyruvate (5.0 mmol, 0.66 mL, *d* = 1.28), 1-(4-bromophenyl)-2-(triphenylphosphoranylidene)ethanone (6.0 mmol, 2.76 g) was added. The mixture was stirred under reflux condition for 4 hours. Then the mixture was concentrated and filtered through silica gel pad and washed with hexane/EtOAc (10/1) to remove triphenylphosphine oxide. The eluent was concentrated and purified by silica gel chromatography (hexane/EtOAc = 50/1) to afford **3e** (2.4 mmol, 0.83 g, 47%): pale yellow solid, mp. 40–41 °C; ^{1}H NMR (500 MHz, $CDCl_3$) δ 7.77–7.74 (m, 2H), 7.68–7.65 (m, 2H), 7.40 (q, *J* = 1.3 Hz, 1H), 4.16 (q, *J* = 7.1 Hz, 2H), 1.11 (t, *J* = 7.1 Hz, 3H); ^{13}C{^{1}H} NMR (125 MHz, $CDCl_3$) δ 189.9, 160.4, 140.9 (q, *J* = 4.8 Hz), 133.7, 132.4, 130.0, 129.8, 129.2 (q, *J* = 32.4 Hz), 120.9 (q, *J* = 273.9 Hz), 62.6, 13.4; ^{19}F NMR (375 MHz, $CDCl_3$) δ –66.10; HRMS (ESI, positive) *m*/*z*: [M + Na]$^{+}$ calcd for $C_{13}H_{10}Br_1F_3O_3Na^{+}$ 372.9657; found 372.9666.

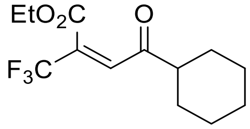

(*E*)-ethyl-4-(cyclohexyl)-4-oxo-2-(trifluoromethyl)but-2-enoate (**3f**): To the solution of ethyl trifluoropyruvate (5.0 mmol, 0.66 mL, *d* = 1.28), 1-cyclohexyl-2-(triphenylphosphoranylidene)ethanone (6.0 mmol, 2.32 g) was added. The mixture was stirred under reflux condition for 4 hours. Then the mixture was concentrated and filtered through silica gel pad and washed with hexane/EtOAc (10/1) to remove triphenylphosphine oxide. The eluent was concentrated and purified by silica gel chromatography (hexane/EtOAc = 20/1) to afford **3f** (2.8 mmol, 0.77 g, 53%): pale yellow oil; ^{1}H NMR (400 MHz, $CDCl_3$) δ 7.07 (s, 1H), 4.30 (q, *J* = 7.0 Hz, 2H), 2.58 (tt, *J* = 3.5, 10.9 Hz, 1H), 2.00–1.90 (m, 2H), 1.86-1.76 (m, 2H), 1.73–1.63 (m, 1H), 1.50–1.10 (m, 8H); ^{13}C{^{1}H} NMR (100 MHz, $CDCl_3$) δ 202.9, 161.1, 140.3 (q, *J* = 4.6 Hz), 128.8 (q, *J* = 32.7 Hz), 120.9 (q, *J* = 273.9 Hz), 62.4, 50.3, 27.7, 25.6, 25.3, 13.6; ^{19}F NMR (375 MHz, $CDCl_3$) δ –65.30; HRMS (ESI, positive) *m*/*z*: [M + H]$^{+}$ calcd for $C_{13}H_{18}F_3O_3^{+}$ 279.1203; found 279.1190.

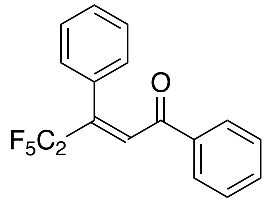

(*E*)-4,4,5,5,5-pentafluoro-1,3-diphenyl-2-penten-1-one (6): To the solution of 2,2,3,3,3-pentafluoro-1-phenyl-1-propanone (5.0 mmol, 1.12 g) in CH_2Cl_2 (15 mL), 1-phenyl-2-(triphenylphosphoranylidene)ethanone (6.0 mmol, 2.28 g) was added. The mixture was stirred at rt for 20 hours. Then the mixture was concentrated and filtered through silica gel pad and washed with hexane/EtOAc (10/1) to remove triphenylphosphine oxide. The eluent was concentrated and purified by silica gel chromatography (hexane/EtOAc = 20/1) to afford **6** (1.84 mmol, 0.60 g, 37%): white solid; mp. 46 °C; ^{1}H NMR (400 MHz, $CDCl_3$) δ 7.82–7.76 (m, 2H), 7.52 (tt, *J* = 1.5, 7.5 Hz, 1H), 7.44–7.35 (m, 2H), 7.31 (t, *J* = 1.5 Hz, 1H), 7.30–7.17 (m, 5H);^{13}C{^{1}H} NMR (100 MHz, $CDCl_3$) δ 191.7, 137.9 (t, *J* = 21.4 Hz), 136.0, 134.3 (t, *J* = 7.6 Hz), 133.8, 130.8, 129.4, 129.2, 128.7, 128.6, 128.1, 118.8 (tq, *J* = 38, 287 Hz), 112.8 (qt, *J* = 38, 256 Hz); ^{19}F NMR (375 MHz, $CDCl_3$) δ –82.17, –114.52; HRMS (APCI, positive) *m/z*: $[M + H]^+$ calcd for $C_{17}H_{12}F_5O^+$ 327.0803; found 327.0803.

3-2. Products

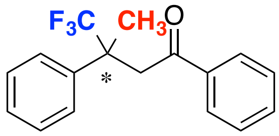

(+)-4,4,4-trifluoro-3-methyl-1,3-diphenylbutan-1-one (2a):

[*Using (E)-**1a***]

$Cu(NO_3)_2{\cdot}3H_2O$ (3.0 mg, 0.0125 mmol, 5 mol%) and **SP** (7.8 mg, 0.0125 mmol, 5 mol%) were dissolved in THF (5.0 mL), and the mixture was stirred at rt for 30 minutes, then the mixture was cooled with ice bath. The solution of Me_3Al in hexane (0.75 mmol, 0.54 mL, 1.4 M) was added dropwise. To the clear yellow solution was added **1a** (69.1 mg, 57 µL, *d* = 1.22, 0.25 mmol) at once. The reaction was carried out at rt and monitored by TLC. After 30 h, a minimal amount of sat. NH_4Cl aq. was added at 0 °C. After stirring at 0 °C for 30 minutes, the mixture was extracted with EtOAc (5 mL x 3). The combined organic layers were dried over anh. Na_2SO_4, filtered and concentrated. The crude product was purified by silica gel (treated with Et_3N) chromatography using hexane/EtOAc (60/1) with 1% Et_3N to afford **2a** (67.3mg, 93%).

[*Using (Z)-**1a***]

$Cu(NO_3)_2{\cdot}3H_2O$ (3.0 mg, 0.0125 mmol, 5 mol%) and **SP** (7.8 mg, 0.0125 mmol, 5 mol%) were dissolved in THF (5.0 mL), and the mixture was stirred at rt for 30 minutes, then the mixture was cooled with ice bath. The solution of Me_3Al in hexane (0.75 mmol, 0.54 mL, 1.4 M) was added dropwise. To the clear yellow solution was added **1a** (69.1 mg, 54 µL, *d* = 1.28, 0.25 mmol) at once. The reaction was carried out at rt and monitored by TLC. After 30 h, a minimal amount of sat. NH_4Cl aq. was added at 0 °C. After stirring at 0 °C for 30 minutes, the mixture was extracted with EtOAc (5 mL x 3). The combined organic layers were dried over anh. Na_2SO_4, filtered and concentrated. The crude product was purified by silica gel (treated with Et_3N) chromatography using hexane/EtOAc (60/1) with 1% Et_3N to afford **2a** (55.5mg, 76%).

colorless oil; ^{1}H NMR (500 MHz, $CDCl_3$) δ 7.92–7.90 (m, 2H), 7.59–7.55 (m, 1H), 7.45 (dd, *J* = 15.6, 8.0 Hz, 4H), 7.34–7.26 (m, 3H), 4.16 (d, *J* = 17.9 Hz, 1H), 3.46 (d, *J* = 17.9 Hz, 1H), 1.88 (s, 3H); ^{13}C{^{1}H} NMR (125 MHz, $CDCl_3$) δ 195.0, 137.4, 137.1, 133.3, 128.7, 128.2, 127.8, 127.8 (q, *J* = 283.1 Hz), 127.6, 127.3, 46.4 (q, *J* = 24.0 Hz), 41.9, 18.8; ^{19}F NMR (375 MHz, $CDCl_3$) δ –77.38; HRMS (ESI, positive) *m*/*z*: $[M + Na]^+$ calcd for $C_{17}H_{15}F_3O_1Na^+$ 315.0967; found 315.0966; (*E*)-**1a**: $[\alpha]^{26.0}_D$ (94% ee) = +110.3 (*c* 0.202, $CHCl_3$), (*Z*)-**1a**: $[\alpha]^{24.0}_D$ (62% ee) = –68.2 (*c* 0.847, $CHCl_3$); HPLC (CHIRALPAK IB, Daicel, 4.6 x 150 mm, hexane/^{i}PrOH=99.5/0.5, 0.8 mL/min, 254 nm), t_r = 8.6 min, t_r = 10.1 min.

Chiral HPLC Chart (racemic) 2a

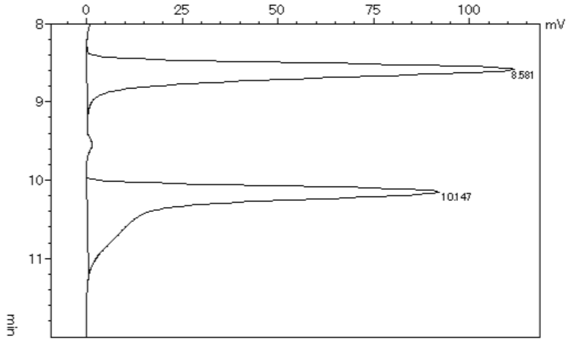

Peak#	Ret. Time	Area	Height	Area %
1	8.581	1493438	111699	49.920
2	10.147	1498204	92112	50.080
Total		2991642	203811	100.000

Chiral HPLC Chart (94% ee (+) using (*E*)-1a) 2a

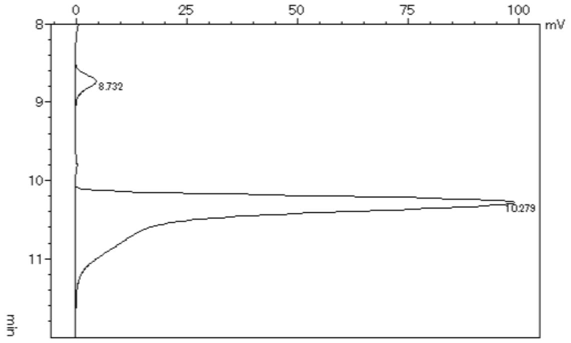

Peak#	Ret. Time	Area	Height	Area %
1	8.732	59759	4641	3.178
2	10.279	1820897	99269	96.822
Total		1880656	103910	100.000

Chiral HPLC Chart (62% ee (-) using (*Z*)-1a) 2a

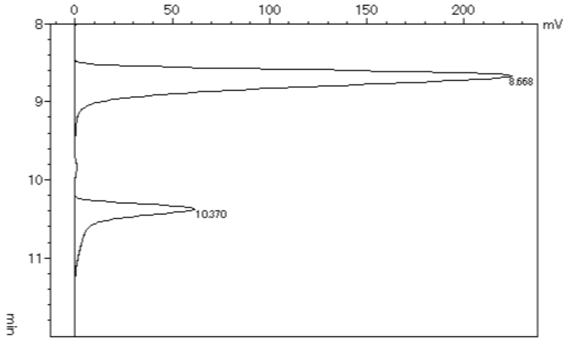

Peak#	Ret. Time	Area	Height	Area %
1	8.668	3363939	225146	80.907
2	10.370	793838	61883	19.093
Total		4157777	287028	100.000

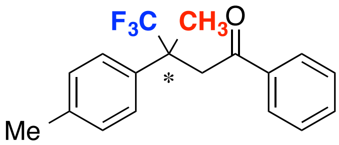

(+)-4,4,4-trifluoro-3-methyl-1-phenyl-3-(*p*-tolyl)butan-1-one (**2b**): $Cu(NO_3)_2{\cdot}3H_2O$ (3.0 mg, 0.0125 mmol, 5 mol%) and **SP** (7.8 mg, 0.0125 mmol, 5 mol%) were dissolved in THF (5.0 mL), and the mixture was stirred at rt for 30 minutes, then the mixture was cooled with ice bath. The solution of Me_3Al in hexane (0.75 mmol, 0.54 mL, 1.4 M) was added dropwise. To the clear yellow solution was added **1b** (72.6 mg, 60 µL, d = 1.20, 0.25 mmol) at once. The reaction was carried out at rt and monitored by TLC. After 48 h, a minimal amount of sat. NH_4Cl aq. was added at 0 °C. After stirring at 0 °C for 30 minutes, the mixture was extracted with EtOAc (5 mL x 3). The combined organic layers were dried over anh. Na_2SO_4, filtered and concentrated. The crude product was purified by silica gel (treated with Et_3N) chromatography using hexane/EtOAc (60/1) with 1% Et_3N to afford **2b** (69.9 mg, 93%): colorless oil; ^{1}H NMR (500 MHz, $CDCl_3$) δ 7.91 (d, J = 8.0 Hz, 2H), 7.58–7.55 (m, 1H), 7.45 (t, J = 7.4 Hz, 2H), 7.31 (d, J = 8.0 Hz, 2H), 7.13 (d, J = 8.0 Hz, 2H), 4.13 (d, J = 17.8 Hz, 1H), 3.44 (d, J = 18.3 Hz, 1H), 2.31 (s, 3H), 1.86 (s, 3H); ^{13}C{^{1}H} NMR (125 MHz, $CDCl_3$) δ 195.1, 137.2, 137.2, 134.4, 133.3, 129.0, 128.6, 127.9 (q, J = 283.1 Hz), 127.8, 127.1, 46.1 (q, J = 24.2 Hz), 41.8, 20.9, 18.8; ^{19}F NMR (375 MHz, $CDCl_3$) δ –77.25; HRMS (ESI, positive) m/z: $[M + Na]^+$ calcd for $C_{18}H_{17}F_3O_1Na^+$ 329.1124; found 329.1135; $[\alpha]^{28.0}_D$ (97% ee) = +113.0 (c 0.287, $CHCl_3$); HPLC (CHIRALPAK IB, Daicel, 4.6 x 150 mm, hexane/^{i}PrOH= 99.5/0.5, 0.8 mL/min, 254 nm), t_r (minor) = 6.3 min, t_r (major) = 7.2 min.

Chiral HPLC Chart (racemic) 2b

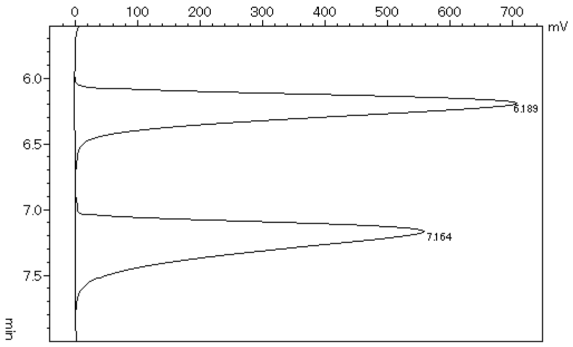

Peak#	Ret. Time	Area	Height	Area %
1	6.189	8384826	708700	50.009
2	7.164	8381934	558825	49.991
Total		16766759	1267525	100.000

Chiral HPLC Chart (97% ee) 2b

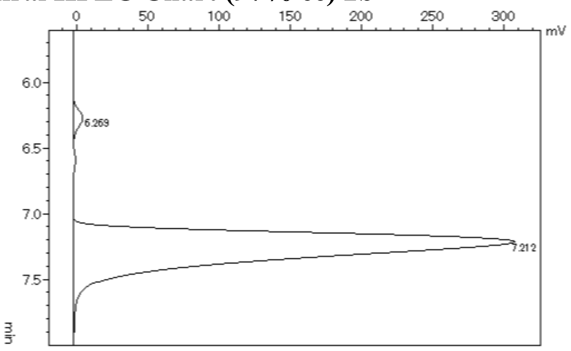

Peak#	Ret. Time	Area	Height	Area %
1	6.269	58084	6483	1.417
2	7.212	4041120	310479	98.583
Total		4099204	316962	100.000

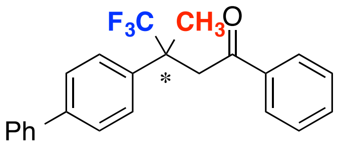

(+)-3-[1,1'-biphenyl]-4-yl-4,4,4-trifluoro-3-methyl-1-phynylbutan-1-one (2c):

[*0.25 mmol scale*]

$Cu(NO_3)_2{\cdot}3H_2O$ (3.0 mg, 0.0125 mmol, 5 mol%) and **SP** (7.8 mg, 0.0125 mmol, 5 mol%) were dissolved in THF (5.0 mL), and the mixture was stirred at rt for 30 minutes, then the mixture was cooled with ice bath. The solution of Me_3Al in hexane (0.75 mmol, 0.54 mL, 1.4 M) was added dropwise. To the clear yellow solution was added **1c** (88.1 mg, 0.25 mmol) at once. The reaction was carried out at rt and monitored by TLC. After 72 h, a minimal amount of sat. NH_4Cl aq. was added at 0 °C. After stirring at 0 °C for 30 minutes, the mixture was extracted with EtOAc (5 mL x 3). The combined organic layers were dried over anh. Na_2SO_4, filtered and concentrated. The crude product was purified by silica gel (treated with Et_3N) chromatography using hexane/EtOAc (60/1) with 1% Et_3N to afford **2c** (64.2 mg, 70%).

[*1 mmol scale*]

$Cu(NO_3)_2{\cdot}3H_2O$ (12.1 mg, 0.050 mmol, 5 mol%) and **SP** (31.0 mg, 0.050 mmol, 5 mol%) were dissolved in THF (20 mL), and the mixture was stirred at rt for 30 minutes, then the mixture was cooled with ice bath. The solution of Me_3Al in hexane (3.0 mmol, 2.14 mL, 1.4 M) was added dropwise. To the clear yellow solution was added **1c** (368.4 mg, 1.05 mmol) at once. The reaction was carried out at rt and monitored by TLC. After 72 h, a minimal amount of sat. NH_4Cl aq. was added at 0 °C. After stirring at 0 °C for 30 minutes, the mixture was extracted with EtOAc (20 mL x 3). The combined organic layers were dried over anh. Na_2SO_4, filtered and concentrated. The crude product was purified by silica gel (treated with Et_3N) chromatography using hexane/EtOAc (20/1) with 1% Et_3N to afford **2c** (286.4 mg, 74%).

white solid; mp. 92 °C; ^1H NMR (400 MHz, $CDCl_3$) δ 7.91 (d, J = 7.3 Hz, 2H), 7.59–7.51 (m, 5H), 7.51–7.36 (m, 6H), 7.31 (t, J = 7.3 Hz, 1H), 4.18 (d, J = 18.0 Hz, 1H), 3.48 (d, J = 18.0 Hz, 1H), 1.91 (s, 3H); $^{13}C\{^1H\}$ NMR (100 MHz, $CDCl_3$) δ 195.0, 140.4, 140.3, 137.2, 136.4, 133.3, 128.7, 127.8 (q, J = 283.11 Hz), 127.8, 127.7, 127.3, 127.1, 126.9, 46.3 (q, J = 24.2 Hz), 41.9, 18.8 (One carbon atom was not found probably due to overlapping); ^{19}F NMR (375 MHz, $CDCl_3$) δ –76.00; HRMS (APCI, positive) *m*/*z*: $[M + H]^+$ calcd for $C_{23}H_{20}F_3O^+$ 369.1461; found 369.1459; $[\alpha]^{28.0}_D$ (97% ee) = +137.7 (*c* 0.103, $CHCl_3$); HPLC (CHIRALCEL OD-H, Daicel, 4.6 x 250 mm, hexane/^{i}PrOH=90/10, 0.3 mL/min, 254 nm), t_r (minor) = 19.2 min, t_r (major) = 20.6 min.

Chiral HPLC Chart (racemic) 2c

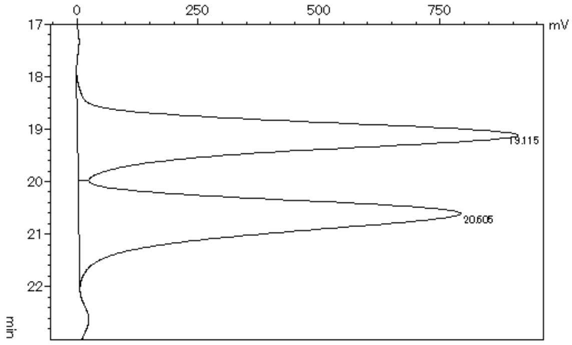

Peak#	Ret. Time	Area	Height	Area %
1	19.115	33535294	910330	50.102
2	20.605	33399397	789547	49.898
Total		66934691	1699876	100.000

Chiral HPLC Chart (97% ee) 2c

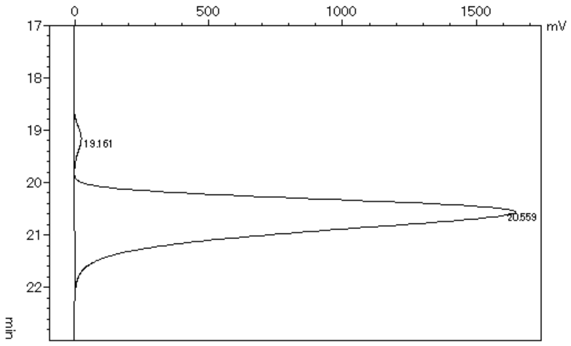

Peak#	Ret. Time	Area	Height	Area %
1	19.161	907117	26988	1.274
2	20.559	70322801	1647391	98.726
Total		71229918	1674379	100.000

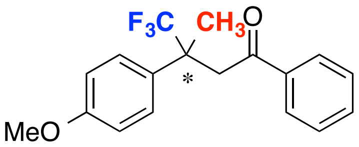

(+)-4,4,4-trifluoro-3-(4-methoxyphenyl)-3-methyl-1-phenylbutan-1-one (**2d**): $Cu(NO_3)_2 \cdot 3H_2O$ (3.0 mg, 0.0125 mmol, 5 mol%) and **SP** (7.8 mg, 0.0125 mmol, 5 mol%) were dissolved in THF (5.0 mL), and the mixture was stirred at rt for 30 minutes, then the mixture was cooled with ice bath. The solution of Me_3Al in hexane (0.75 mmol, 0.54 mL, 1.4 M) was added dropwise. To the clear yellow solution was added **1d** (76.6 mg, 61 µL, *d* = 1.26, 0.25 mmol) at once. The reaction was carried out at rt and monitored by TLC. After 96 h, a minimal amount of sat. NH_4Cl aq. was added at 0 °C. After stirring at 0 °C for 30 minutes, the mixture was extracted with EtOAc (5 mL x 3). The combined organic layers were dried over anh. Na_2SO_4, filtered and concentrated. The crude product was purified by silica gel (treated with Et_3N) chromatography using hexane/EtOAc (60/1) with 1% Et_3N to afford **2d** (68.9 mg, 87%): colorless oil; ^{1}H NMR (500 MHz, $CDCl_3$) δ 7.91–7.89 (m, 2H), 7.58–7.54 (m, 1H), 7.47–7.44 (m, 2H), 7.34 (d, J = 8.6 Hz, 2H), 6.85 (dt, J = 6.3, 3.8 Hz, 2H), 4.10 (d, J = 17.8 Hz, 1H), 3.77 (s, 3H), 3.43 (d, J = 17.8 Hz, 1H), 1.85 (s, 3H); $^{13}C\{^1H\}$ NMR (125 MHz, $CDCl_3$) δ 195.1, 158.7, 137.2, 133.3, 129.3, 128.6, 128.4, 127.9 (q, J = 283.1 Hz), 127.8, 113.6, 55.1, 45.9 (q, J = 24.4 Hz), 41.8, 18.9; ^{19}F NMR (470 MHz, $CDCl_3$) δ –79.11; HRMS (ESI, positive) *m/z*: $[M + Na]^+$ calcd for $C_{18}H_{17}F_3O_2Na^+$ 345.1073; found 345.1080; $[\alpha]^{28.0}_D$ (93% ee) = +118.4 (*c* 0.137, $CHCl_3$); HPLC (CHIRALPAK AD, Daicel, 4.6 x 250 mm, hexane/^{i}PrOH= 99.0/1.00, 1.0 mL/min, 254 nm), t_r (minor) = 15.0 min, t_r (major) = 26.9 min.

Chiral HPLC Chart (racemic) 2d

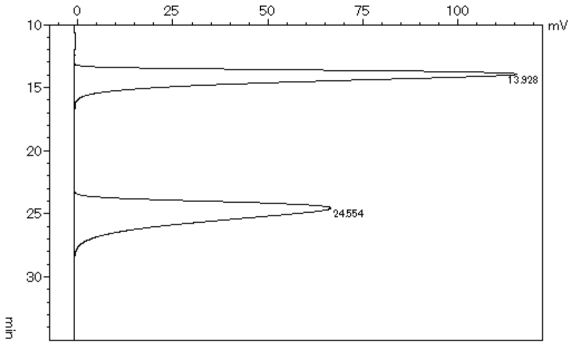

Peak#	Ret. Time	Area	Height	Area %
1	13.928	7237884	116666	50.110
2	24.554	7206229	67711	49.890
Total		14444113	184377	100.000

Chiral HPLC Chart (93% ee) 2d

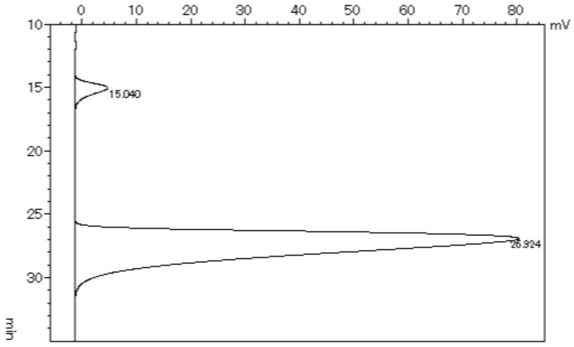

Peak#	Ret. Time	Area	Height	Area %
1	15.040	364274	6014	3.552
2	26.924	9891639	81787	96.448
Total		10255912	87801	100.000

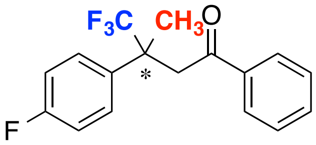

(+)-4,4,4-trifluoro-3-(4-fluorophenyl)-3-methyl-1-phenylbutan-1-one (**2e**): $Cu(NO_3)_2{\cdot}3H_2O$ (3.0 mg, 0.0125 mmol, 5 mol%) and **SP** (7.8 mg, 0.0125 mmol, 5 mol%) were dissolved in THF (5.0 mL), and the mixture was stirred at rt for 30 minutes, then the mixture was cooled with ice bath. The solution of Me_3Al in hexane (0.75 mmol, 0.54 mL, 1.4 M) was added dropwise. To the clear yellow solution was added **1e** (73.6 mg, 58 μL, *d* = 1.28, 0.25 mmol) at once. The reaction was carried out at rt and monitored by TLC. After 48 h, a minimal amount of sat. NH_4Cl aq. was added at 0 °C. After stirring at 0 °C for 30 minutes, the mixture was extracted with EtOAc (5 mL x 3). The combined organic layers were dried over anh. Na_2SO_4, filtered and concentrated. The crude product was purified by silica gel (treated with Et_3N) chromatography using hexane/EtOAc (60/1) with 1% Et_3N to afford **2e** (69.4 mg, 89%): colorless oil; ^{1}H NMR (500 MHz, $CDCl_3$) δ 7.91–7.89 (m, 2H), 7.59–7.56 (m, 1H), 7.47–7.44 (m, 2H), 7.39 (q, *J* = 4.6 Hz, 2H), 7.03–6.98 (m, 2H), 4.10 (d, *J* = 18.3 Hz, 1H), 3.47 (d, *J* = 17.8 Hz, 1H), 1.86 (s, 3H); $^{13}C\{^1H\}$ NMR (125 MHz, $CDCl_3$) δ 194.9, 162.1 (d, *J* = 245.9 Hz), 137.0, 133.5, 133.2, 129.1 (d, *J* = 8.4 Hz), 128.7, 127.8, 127,6 (q, *J* = 283.1 Hz), 115.1 (d, *J* = 21.6 Hz), 46.0 (q, *J* = 24.4 Hz), 41.9, 18.9; ^{19}F NMR (470 MHz, $CDCl_3$) δ –77.39, –116.11; HRMS (ESI, positive) *m*/*z*: $[M + Na]^+$ calcd for $C_{17}H_{14}F_4O_1Na^+$ 333.0873; found 333.0874; $[\alpha]^{27.0}_D$ (96% ee) = +113.4 (*c* 0.460, $CHCl_3$); HPLC (CHIRALPAK IB, Daicel, 4.6 x 150 mm, hexane/^{i}PrOH=99.5/0.5, 0.7 mL/min, 254 nm), t_r (minor) = 10.7 min, t_r (major) = 11.2 min.

Chiral HPLC Chart (racemic) 2e

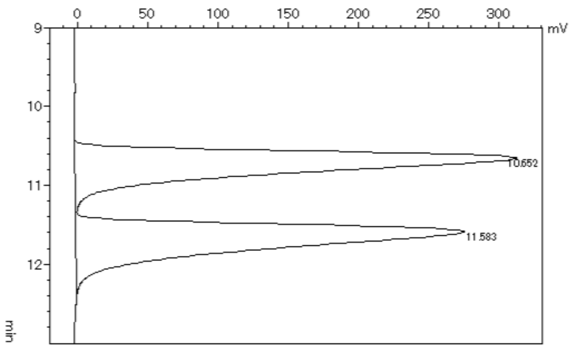

Peak#	Ret. Time	Area	Height	Area %
1	10.652	5706468	314784	49.947
2	11.583	5718565	276969	50.053
Total		11425033	591753	100.000

Chiral HPLC Chart (96% ee) 2e

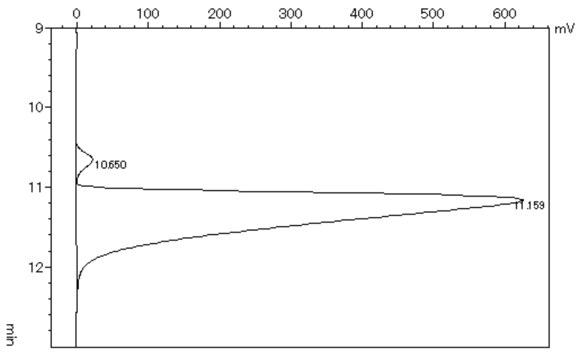

Peak#	Ret. Time	Area	Height	Area %
1	10.650	321830	23740	1.899
2	11.159	16629809	628134	98.101
Total		16951639	651874	100.000

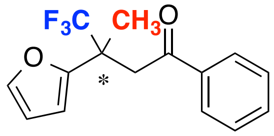

(+)-4,4,4-trifluoro-3-(furan-2-yl)-3-methyl-1-phenylbutan-1-one (**2g**): $Cu(NO_3)_2{\cdot}3H_2O$ (3.0 mg, 0.0125 mmol, 5 mol%) and **SP** (7.8 mg, 0.0125 mmol, 5 mol%) were dissolved in THF (5.0 mL), and the mixture was stirred at rt for 30 minutes, then the mixture was cooled with ice bath. The solution of Me_3Al in hexane (0.75 mmol, 0.54 mL, 1.4 M) was added dropwise. To the clear yellow solution was added **1g** (66.6 mg, 52 µL, *d* = 1.29, 0.25 mmol) at once. The reaction was carried out at rt and monitored by TLC. After 21 h, a minimal amount of sat. NH_4Cl aq. was added at 0 °C. After stirring at 0 °C for 30 minutes, the mixture was extracted with EtOAc (5 mL x 3). The combined organic layers were dried over anh. Na_2SO_4, filtered and concentrated. The crude product was purified by silica gel (treated with Et_3N) chromatography using hexane/EtOAc (60/1) with 1% Et_3N to afford **2g** (44.4 mg, 62%): pale yellow oil; ^{1}H NMR (500 MHz, $CDCl_3$) δ 7.91–7.89 (m, 2H), 7.57–7.54 (m, 1H), 7.46–7.43 (m, 2H), 7.30 (d, *J* = 1.7 Hz, 1H), 6.34–6.31 (m, 2H), 4.01 (d, *J* = 17.2 Hz, 1H), 3.31 (d, *J* = 17.2 Hz, 1H), 1.75 (s, 3H); ^{13}C{^{1}H} NMR (125 MHz, $CDCl_3$) δ 195.3, 151.2, 142.1, 137.0, 133.3, 128.6, 127.9,126.8 (q, *J* = 283.9 Hz), 110.6, 108.4, 44.4 (q, *J* = 26.0 Hz), 39.9, 17.2; ^{19}F NMR (470 MHz, $CDCl_3$) δ –77.33; HRMS (ESI, positive) *m*/*z*: $[M + Na]^+$ calcd for $C_{15}H_{13}F_3O_2Na^+$ 305.0760; found 305.0764; $[\alpha]^{24.0}_D$ (94% ee) = +42.34 (*c* 1.00, $CHCl_3$); HPLC (CHIRALPAK IB, Daicel, 4.6 x 150 mm, hexane/^{i}PrOH=99.5/0.5, 0.7 mL/min, 254 nm), t_r (minor) = 17.1 min, t_r (major) = 22.0 min.

Chiral HPLC Chart (racemic) 2g

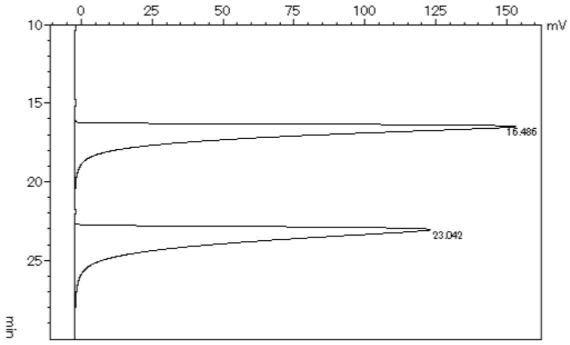

Peak#	Ret. Time	Area	Height	Area %
1	16.486	8269120	155666	49.795
2	23.042	8337215	125388	50.205
Total		16606335	281054	100.000

Chiral HPLC Chart (94% ee) 2g

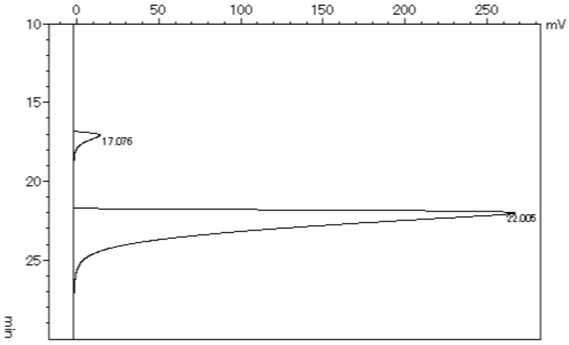

Peak#	Ret. Time	Area	Height	Area %
1	17.076	624253	16397	2.959
2	22.005	20472419	269556	97.041
Total		21096672	285953	100.000

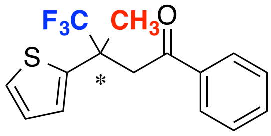

(+)-4,4,4-trifluoro-3-methyl-1-phenyl-3-(thiophen-2-yl)butan-1-one (**2h**): $Cu(NO_3)_2·3H_2O$ (3.0 mg, 0.0125 mmol, 5 mol%) and **SP** (7.8 mg, 0.0125 mmol, 5 mol%) were dissolved in THF (5.0 mL), and the mixture was stirred at rt for 30 minutes, then the mixture was cooled with ice bath. The solution of Me_3Al in hexane (0.75 mmol, 0.54 mL, 1.4 M) was added dropwise. To the clear yellow solution was added **1h** (70.6 mg, 54 µL, *d* = 1.31, 0.25 mmol) at once. The reaction was carried out at rt and monitored by TLC. After 96 h, a minimal amount of sat. NH_4Cl aq. was added at 0 °C. After stirring at 0 °C for 30 minutes, the mixture was extracted with EtOAc (5 mL x 3). The combined organic layers were dried over anh. Na_2SO_4, filtered and concentrated. The crude product was purified by silica gel (treated with Et_3N) chromatography using hexane/EtOAc (60/1) with 1% Et_3N to afford **2h** (48.4 mg, 64%): colorless oil; ^{1}H NMR (500 MHz, $CDCl_3$) δ 7.89–7.87 (m, 2H), 7.57–7.53 (m, 1H), 7.45–7.42 (m, 2H), 7.23 (t, *J* = 4.9 Hz, 1H), 7.03 (d, *J* = 3.4 Hz, 1H), 6.94 (dd, *J* = 4.9, 3.7 Hz, 1H), 3.92 (d, *J* = 17.2 Hz, 1H), 3.45 (d, *J* = 17.2 Hz, 1H), 1.92 (s, 3H); $^{13}C\{^1H\}$ NMR (125 MHz, $CDCl_3$) δ 195.0, 141.7, 137.2, 133.3, 128.6, 127.9, 127.1 (q, *J* = 238.1 Hz) 126.7, 126.3, 125.1, 45.6 (q, *J* = 26.0 Hz), 42.7, 19.5; ^{19}F NMR (375 MHz,$CDCl_3$) δ –77.98; HRMS (ESI, positive) *m*/*z*: $[M + Na]^+$ calcd for $C_{15}H_{13}F_3O_1S_1Na^+$ 321.0531; found 321.0530; $[\alpha]^{24.0}_D$ (94% ee) = +82.80 (*c* 1.00, $CHCl_3$); HPLC (CHIRALPAK IB, Daicel, 4.6 x 150 mm, hexane/^{i}PrOH = 99.5/0.5, 0.8 mL/min, 254 nm), tr (minor) = 22.7 min, tr (major) = 27.9 min.

Chiral HPLC Chart (racemic) 2h

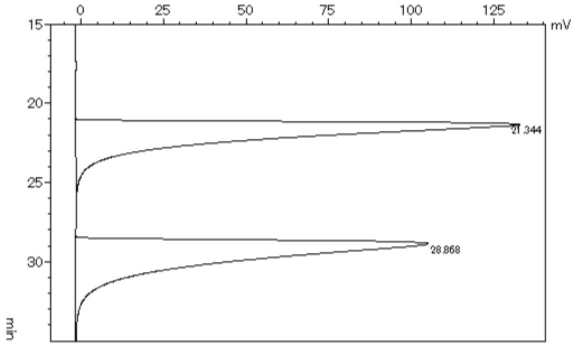

Peak#	Ret. Time	Area	Height	Area %
1	21.344	9254614	134454	50.019
2	28.868	9247662	106636	49.981
Total		18502276	241090	100.000

Chiral HPLC Chart (94%ee) 2h

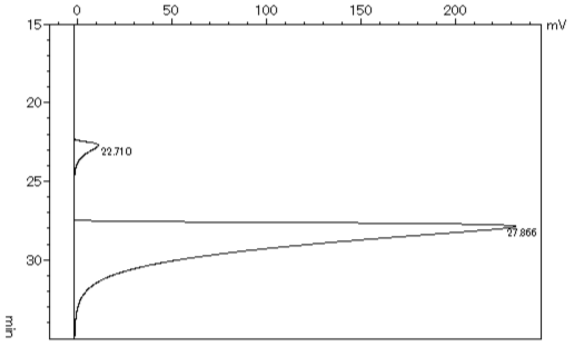

Peak#	Ret. Time	Area	Height	Area %
1	22.710	717601	13059	2.893
2	27.866	24086521	234072	97.107
Total		24804122	247131	100.000

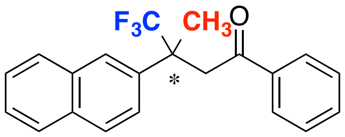

(+)-4,4,4-trifluoro-3-methyl-3-(2-naphthalenyl)-1-phenylbutan-1-one (2i): $Cu(NO_3)_2 \cdot 3H_2O$ (3.0 mg, 0.0125 mmol, 5 mol%) and **SP** (7.8 mg, 0.0125 mmol, 5 mol%) were dissolved in THF (5.0 mL), and the mixture was stirred at rt for 30 minutes, then the mixture was cooled with ice bath. The solution of Me_3Al in hexane (0.75 mmol, 0.54 mL, 1.4 M) was added dropwise. To the clear yellow solution was added **1i** (81.6 mg, 67 µL, *d* = 1.21, 0.25 mmol) at once. The reaction was carried out at rt and monitored by TLC. After 72 h, a minimal amount of sat. NH_4Cl aq. was added at 0 °C. After stirring at 0 °C for 30 minutes, the mixture was extracted with EtOAc (5 mL x 3). The combined organic layers were dried over anh. Na_2SO_4, filtered and concentrated. The crude product was purified by silica gel (treated with Et_3N) chromatography using hexane/EtOAc (60/1) with 1% Et_3N to afford **2i** (52.1 mg, 60%): white solid; mp. 117 °C; ^{1}H NMR (400 MHz, $CDCl_3$) δ 7.92–7.86 (m, 3H), 7.82–7.74 (m, 3H), 7.57–7.50 (m, 2 H), 7.47–7.39 (m, 4H), 4.27 (d, J = 18.0 Hz, 1H), 3.54 (d, J = 18.0 Hz, 1H), 2.00 (s, 3H) ^{13}C{^{1}H} NMR (100 MHz, $CDCl_3$) δ 194.9, 137.2, 134.9, 133.3, 133.0, 132.5, 128.6, 128.3, 127.9 (q, J = 283.4 Hz), 127.8, 127.4, 127.0, 126.2, 126.1, 124.8, 46.6 (q, J = 24.0 Hz), 42.0, 19.0 (One carbon atom was not found probably due to overlapping); ^{19}F NMR (375 MHz, $CDCl_3$) δ –75.76; HRMS (APCI, positive) *m*/*z*: $[M + H]^+$ calcd for $C_{21}H_{18}F_3O^+$ 343.1304; found 343.1305; $[\alpha]^{28.0}_{D}$ (88% ee) = +155.15 (*c* 0.1977); HPLC (CHIRALCEL OD-H, Daicel, 4.6 x 250 mm, hexane/^{i}PrOH=99/1, 0.3 mL/min, 254 nm), t_r (minor) = 25.5 min, t_r (major) = 27.1 min.

Chiral HPLC Chart (racemic) 2i

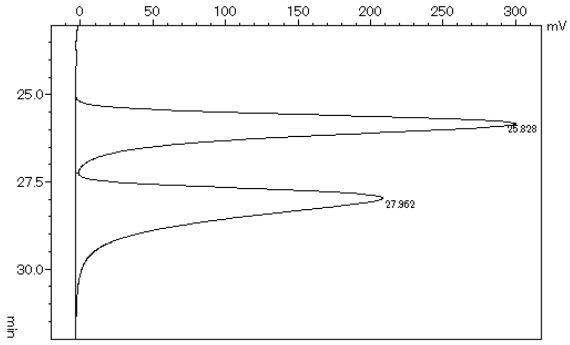

Peak#	Ret. Time	Area	Height	Area %
1	25.828	12893974	303781	50.358
2	27.962	12710593	211590	49.642
Total		25604567	515370	100.000

Chiral HPLC Chart (88% ee) 2i

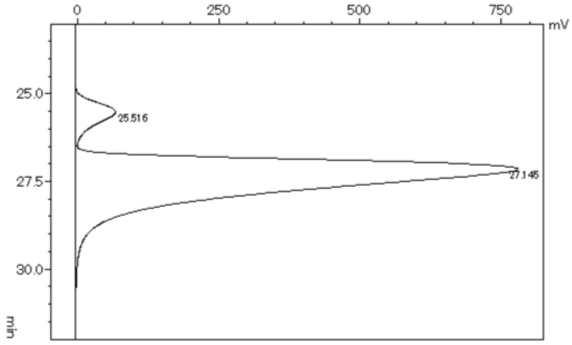

Peak#	Ret. Time	Area	Height	Area %
1	25.516	3051726	71237	6.132
2	27.145	46717581	783371	93.868
Total		49769306	854608	100.000

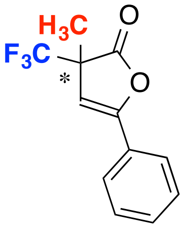

(–)-3-methyl-5-phenyl-3-(trifluoromethyl)furan-2(3*H*)-one (**4a**):

[*Using (E)-**3a***]

CuCl (1.2 mg, 0.0125 mmol, 5 mol%) and **SP** (7.8 mg, 0.0125 mmol, 5 mol%) were dissolved in THF (5.0 mL), and the mixture was stirred at rt for 30 minutes, then the mixture was cooled with ice bath. The solution of Me_3Al in hexane (0.75 mmol, 0.54 mL, 1.4 M) was added dropwise. To the colorless solution was added **3a** (68.0 mg, 55 µL, *d* = 1.23, 0.25 mmol) at once. The reaction was carried out at rt and monitored by TLC. After 0.5 h, a minimal amount of sat. NH_4Cl aq. was added at 0 °C. After stirring at 0 °C for 30 minutes, the mixture was filtered through silica gel pad and washed with Et_2O and the eluent was concentrated. The crude product was purified by silica gel chromatography using hexane/EtOAc (30/1) to afford **4a** (52.6 mg, 87%).

[*Using (Z)-**3a***]

CuCl (1.2 mg, 0.0125 mmol, 5 mol%) and **SP** (7.8 mg, 0.0125 mmol, 5 mol%) were dissolved in THF (5.0 mL), and the mixture was stirred at rt for 30 minutes, then the mixture was cooled with ice bath. The solution of Me_3Al in hexane (0.75 mmol, 0.54 mL, 1.4 M) was added dropwise. To the colorless solution was added **3a** (68.0 mg, 54 µL, *d* = 1.25, 0.25 mmol) at once. The reaction was carried out at rt and monitored by TLC. After 2 h, a minimal amount of sat. NH_4Cl aq. was added at 0 °C. After stirring at 0 °C for 30 minutes, the mixture was filtered through silica gel pad and washed with Et_2O and the eluent was concentrated. The crude product was purified by silica gel chromatography using hexane/EtOAc (30/1) to afford **4a** (40.6 mg, 67%).

white solid, mp. 45–46 °C; ^{1}H NMR (500 MHz, $CDCl_3$) δ 7.65–7.63 (m, 2H), 7.45 (dd, *J* = 5.2, 1.7 Hz, 3H), 5.83 (s, 1H), 1.66 (s, 3H); ^{13}C{^{1}H} NMR (125 MHz, $CDCl_3$) δ 172.2, 155.4, 130.8, 128.9, 127.0, 125.3, 124.1 (q, *J* = 281.9 Hz), 100.2, 53.9 (q, *J* = 29.6 Hz), 17.8; ^{19}F NMR (375 MHz, $CDCl_3$) δ –74.68; HRMS (ESI, positive) *m*/*z*: $[M + Na]^+$ calcd for $C_{12}H_9F_3O_2Na^+$ 265.0447; found 265.0449; (*E*)-**3a**: $[\alpha]^{28.0}_D$ (97% ee) = –18.12 (*c* 1.00, $CHCl_3$), (*Z*)-**3a**: $[\alpha]^{25.0}_D$ (92% ee) = +19.36 (*c* 1.27, $CHCl_3$); HPLC (CHIRALPAK IA, Daicel, 4.6 x 250 mm, hexane/^{i}PrOH = 99.9/0.1, 1.0 mL/min, 254 nm), t_r = 7.1 min, t_r = 8.2 min.

Chiral HPLC Chart (racemic) 4a

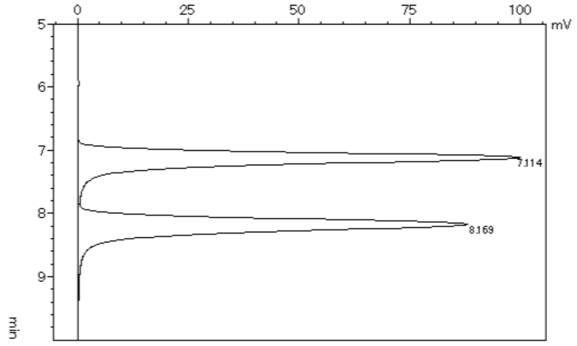

Peak#	Ret. Time	Area	Height	Area %
1	7.114	1299793	100026	50.379
2	8.169	1280247	88112	49.621
Total		2580040	188138	100.000

Chiral HPLC Chart (97% ee (−) using (*E*)-3a) 4a

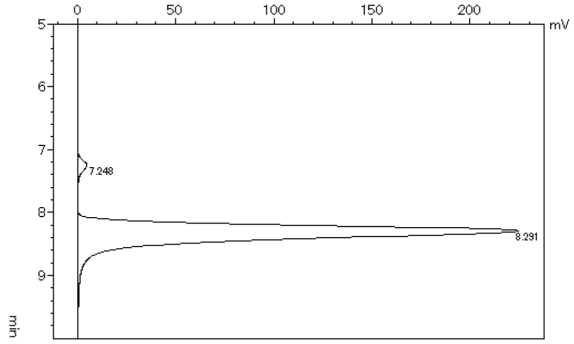

Peak#	Ret. Time	Area	Height	Area %
1	7.248	58752	4478	1.703
2	8.291	3391513	224613	98.297
Total		3450264	229091	100.000

Chiral HPLC Chart (92% ee (+) using (*Z*)-3a) 4a

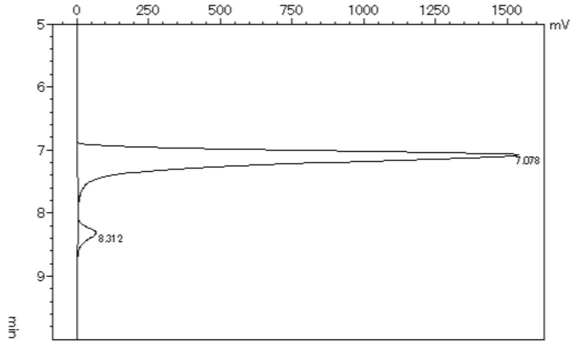

Peak#	Ret. Time	Area	Height	Area %
1	7.078	22203854	1539179	96.045
2	8.312	914245	63399	3.955
Total		23118099	1602578	100.000

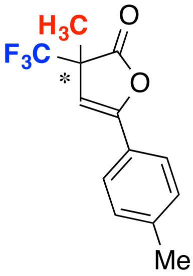

(–)-3-methyl-5-(*p*-tolyl)-3-(trifluoromethyl)furan-2(3*H*)-one (**4b**): CuCl (1.2 mg, 0.0125 mmol, 5 mol%) and **SP** (7.8 mg, 0.0125 mmol, 5 mol%) were dissolved in THF (5.0 mL), and the mixture was stirred at rt for 30 minutes, then the mixture was cooled with ice bath. The solution of Me_3Al in hexane (0.75 mmol, 0.54 mL, 1.4 M) was added dropwise. To the colorless solution was added **3b** (71.6 mg, 53 µL, *d* = 1.36, 0.25 mmol) at once. The reaction was carried out at rt and monitored by TLC. After 0.5 h, a minimal amount of sat. NH_4Cl aq. was added at 0 °C. After stirring at 0 °C for 30 minutes, the mixture was filtered through silica gel pad and washed with Et_2O and the eluent was concentrated. The crude product was purified by silica gel chromatography using hexane/EtOAc (30/1) to afford **4b** (48.7 mg, 85%): white solid, mp. 79–80 °C; ^1H NMR (500 MHz, $CDCl_3$) δ 7.53 (d, J = 8.6 Hz, 2H), 7.26–7.24 (m, 2H), 5.76 (s, 1H), 2.40 (s, 3H), 1.65 (s, 3H); $^{13}C\{^1H\}$ NMR (125 MHz, $CDCl_3$) δ 172.4, 155.5, 141.1, 129.5, 125.2, 124.2,124.1 (q, J = 281.9 Hz), 99.1, 53.8 (q, J = 29.6 Hz), 21.5, 17.9; ^{19}F NMR (375 MHz, $CDCl_3$) δ –74.74; HRMS (ESI, positive) *m*/*z*: $[M + Na]^+$ calcd for $C_{13}H_{11}F_3O_2Na^+$ 279.0603; found 279.0598; $[\alpha]^{28.0}_D$ (96% ee) = –19.21 (*c* 1.04, $CHCl_3$); HPLC (CHIRALPAK AD, Daicel, 4.6 x 250 mm, hexane/^{i}PrOH = 99.9/0.1, 1.0 mL/min, 254 nm), t_r (minor) = 11.3 min, t_r (major) = 13.6 min.

Chiral HPLC Chart (racemic) 4b

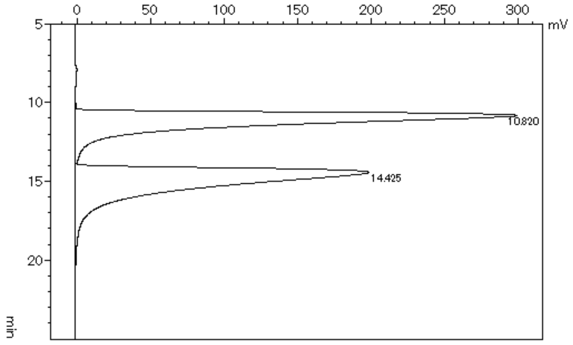

Peak#	Ret. Time	Area	Height	Area %
1	10.820	16053105	300402	50.027
2	14.425	16035847	199777	49.973
Total		32088952	500178	100.000

Chiral HPLC Chart (96% ee) 4b

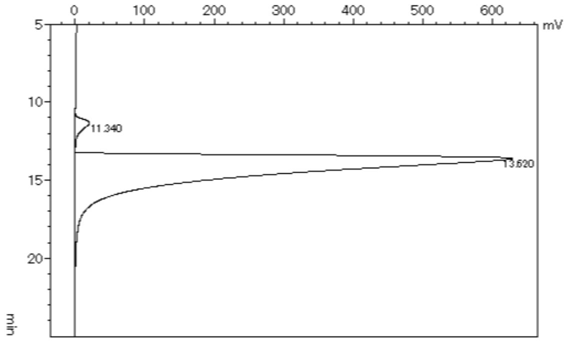

Peak#	Ret. Time	Area	Height	Area %
1	11.340	951592	20341	1.829
2	13.620	51082615	629676	98.171
Total		52034207	650017	100.00

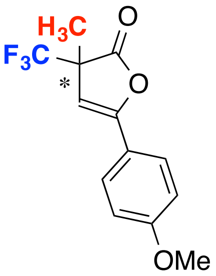

(–)-5-(4-methoxyphenyl)-3-methyl-3-(trifluoromethyl)furan-2(3*H*)-one (**4c**): CuCl (1.2 mg, 0.0125 mmol, 5 mol%) and **SP** (7.8 mg, 0.0125 mmol, 5 mol%) were dissolved in THF (5.0 mL), and the mixture was stirred at rt for 30 minutes, then the mixture was cooled with ice bath. The solution of Me_3Al in hexane (0.75 mmol, 0.54 mL, 1.4 M) was added dropwise. To the colorless solution was added **3c** (75.6 mg, 61 µL, d = 1.23, 0.25 mmol) at once. The reaction was carried out at rt and monitored by TLC. After 1 h, a minimal amount of sat. NH_4Cl aq. was added at 0 °C. After stirring at 0 °C for 30 minutes, the mixture was filtered through silica gel pad and washed with Et_2O and the eluent was concentrated. The crude product was purified by silica gel chromatography using hexane/EtOAc (10/1) to afford **4c** (57.1 mg, 83%): white solid, mp. 92–93 °C; ^{1}H NMR (500 MHz, $CDCl_3$) δ 7.59–7.56 (m, 2H), 6.97–6.94 (m, 2H), 5.67 (s, 1H), 3.86 (s, 3H), 1.65 (s, 3H); $^{13}C\{^1H\}$ NMR (125 MHz, $CDCl_3$) δ 172.5, 161.4, 155.2, 126.9, 124.1 (q, J = 281.9 Hz), 119.5, 114.2, 97.8, 55.4, 53.8 (q, J = 29.6 Hz), 17.9; ^{19}F NMR (375 MHz, $CDCl_3$) δ –75.82; HRMS (ESI, positive) m/z: $[M + Na]^+$ calcd for $C_{13}H_{11}F_3O_3Na^+$ 295.0553; found 295.0556; $[\alpha]^{28.0}_D$ (96% ee) = –19.85 (c 1.00, $CHCl_3$); HPLC (CHIRALPAK AD, Daicel, 4.6 x 250 mm, hexane/^{i}PrOH = 99/1, 0.5 mL/min, 254 nm), t_r (minor) = 23.4 min, t_r (major) = 26.9 min.

Chiral HPLC Chart (racemic) 4c

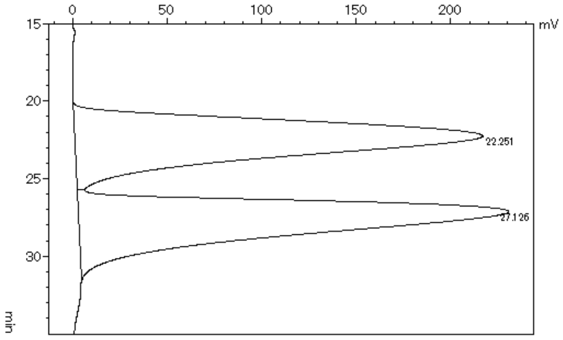

Peak#	Ret. Time	Area	Height	Area %
1	22.251	32377477	216075	50.103
2	27.126	32244850	227865	49.897
Total		64622327	443940	100.000

Chiral HPLC Chart (96% ee) 4c

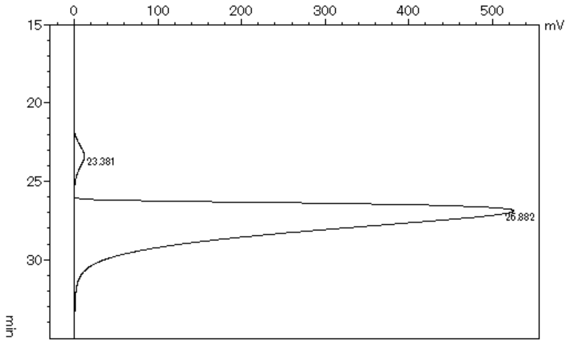

Peak#	Ret. Time	Area	Height	Area %
1	23.381	1310223	12425	2.004
2	26.882	64077175	526029	97.996
Total		65387398	538454	100.000

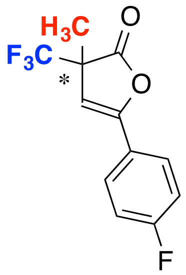

(–)-5-(4-fluorophenyl)-3-methyl-3-(trifluoromethyl)furan-2(3*H*)-one (**4d**): CuCl (1.2 mg, 0.0125 mmol, 5 mol%) and **SP** (7.8 mg, 0.0125 mmol, 5 mol%) were dissolved in THF (5.0 mL), and the mixture was stirred at rt for 30 minutes, then the mixture was cooled with ice bath. The solution of Me_3Al in hexane (0.75 mmol, 0.54 mL, 1.4 M) was added dropwise. To the colorless solution was added **3d** (72.6 mg, 56 µL, *d* = 1.29, 0.25 mmol) at once. The reaction was carried out at rt and monitored by TLC. After 2 h, a minimal amount of sat. NH_4Cl aq. was added at 0 °C. After stirring at 0 °C for 30 minutes, the mixture was filtered through silica gel pad and washed with Et_2O and the eluent was concentrated. The crude product was purified by silica gel chromatography using hexane/EtOAc (20/1) to afford **4d** (59.1 mg, 90%): pale yellow oil; ^{1}H NMR (500 MHz, $CDCl_3$) δ 7.64 (tt, *J* = 7.4, 2.4 Hz, 2H), 7.17–7.12 (m, 2H), 5.77 (s, 1H), 1.67 (s, 3H); $^{13}C\{^1H\}$ NMR (125 MHz, $CDCl_3$) δ 172.0, 164.0 (d, *J* = 251.9 Hz), 154.5, 127.4 (d, *J* = 8.4 Hz), 124.0 (q, *J* = 281.9 Hz) 123.3 (d, *J* = 3.6 Hz), 116.1 (d, *J* = 21.6 Hz), 99.8, 53.9 (q, *J* = 29.6 Hz), 17.8; ^{19}F NMR (375 MHz, $CDCl_3$) δ –74.71, –109.34; HRMS (ESI, positive) *m*/*z*: $[M + Na]^+$ calcd for $C_{12}H_8F_4O_2Na^+$ 283.0353; found 283.0377; $[\alpha]^{28.0}_D$ (97% ee) = –17.61 (*c* 1.16, $CHCl_3$); HPLC (CHIRALPAK AD, Daicel, 4.6 x 250 mm, hexane/^{i}PrOH = 99.9/0.1, 1.0 mL/min, 254 nm), t_r (minor) = 13.6 min, t_r (major) = 14.8 min.

Chiral HPLC Chart (racemic) 4d

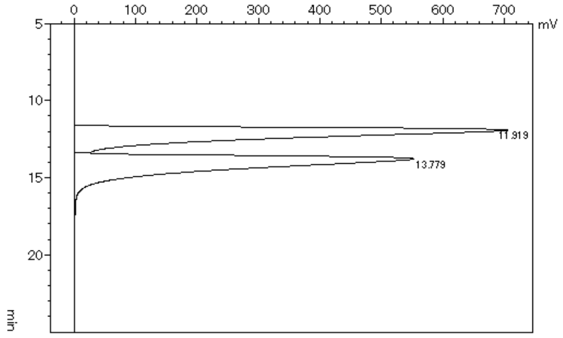

Peak#	Ret. Time	Area	Height	Area %
1	11.919	32045633	705922	50.318
2	13.779	31640266	552831	49.682
Total		63685899	1258753	100.000

Chiral HPLC Chart (97% ee) 4d

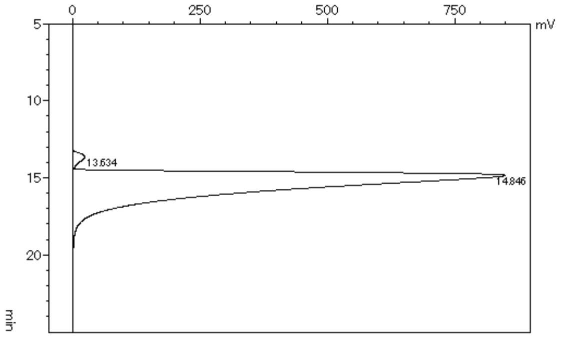

Peak#	Ret. Time	Area	Height	Area %
1	13.634	990028	24172	1.462
2	14.846	66712710	850006	98.538
Total		67702738	874178	100.000

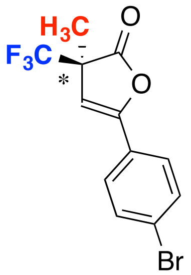

(*R*)-(–)-5-(4-bromophenyl)-3-methyl-3-(trifluoromethyl)furan-2(3*H*)-one (**4e**): CuCl (1.2 mg, 0.0125 mmol, 5 mol%) and **SP** (7.8 mg, 0.0125 mmol, 5 mol%) were dissolved in THF (5.0 mL), and the mixture was stirred at rt for 30 minutes, then the mixture was cooled with ice bath. The solution of Me_3Al in hexane (0.75 mmol, 0.54 mL, 1.4 M) was added dropwise. To the colorless solution was added **3e** (87.8 mg, 60 µL, d = 1.46, 0.25 mmol) at once. The reaction was carried out at rt and monitored by TLC. After 2 h, a minimal amount of sat. NH_4Cl aq. was added at 0 °C. After stirring at 0 °C for 30 minutes, the mixture was filtered through silica gel pad and washed with Et_2O and the eluent was concentrated. The crude product was purified by silica gel chromatography using hexane/EtOAc (20/1) to afford **4e** (73.5 mg, 89%): white solid, mp 57–58 °C; ^{1}H NMR (500 MHz, $CDCl_3$) δ 7.58 (dt, *J* = 8.8, 2.1 Hz, 2H), 7.50 (dt, *J* = 8.8, 2.1 Hz, 2H), 5.84 (s, 1H), 1.66 (s, 3H); ^{13}C NMR{^{1}H} (125 MHz, $CDCl_3$) δ 171.8, 154.5, 132.2, 126.8, 125.9, 125.1,123.9 (q, *J* = 281.9 Hz), 100.8, 54.0 (q, *J* = 29.6 Hz), 17.8; ^{19}F NMR (375 MHz, $CDCl_3$) δ –74.61; HRMS (ESI, positive) *m*/*z*: $[M + Na]^+$ calcd for $C_{12}H_8Br_1F_3O_2Na^+$ 342.9552; found 342.9554; $[\alpha]^{29.0}_D$ (97% ee) = –18.70 (*c* 1.01, $CHCl_3$); HPLC (CHIRALPAK AD, Daicel, 4.6 x 250 mm, hexane/^{i}PrOH = 99.9/0.1, 1.0 mL/min, 254 nm), t_r (minor) = 16.2 min, t_r (major) = 17.7 min.

Chiral HPLC Chart (racemic) 4e

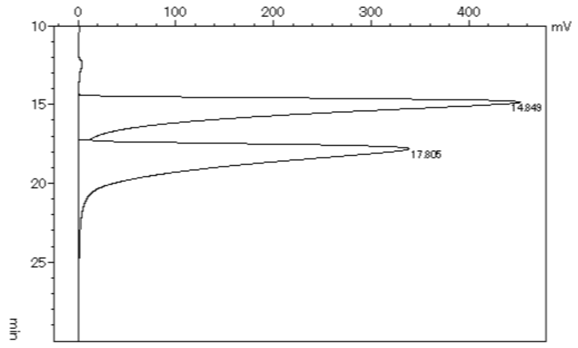

Peak#	Ret. Time	Area	Height	Area %
1	14.849	31005604	452798	50.347
2	17.805	30578061	338720	49.653
Total		61583665	791518	100.000

Chiral HPLC Chart (97% ee) 4e

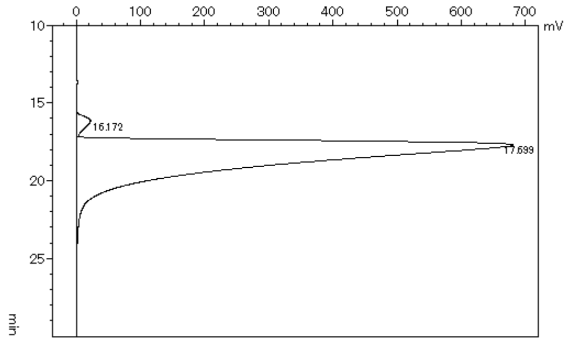

Peak#	Ret. Time	Area	Height	Area %
1	16.172	1161397	22109	1.675
2	17.699	68190229	681201	98.325
Total		69351626	703310	100.000

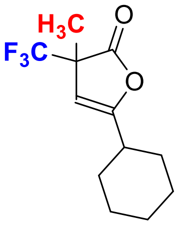

(+)-3-methyl-5-cyclohexyl-3-(trifluoromethyl)furan-2(3*H*)-one (**4f**): CuCl (1.2 mg, 0.0125 mmol, 5 mol%) and **SP** (7.8 mg, 0.0125 mmol, 5 mol%) were dissolved in THF (5.0 mL), and the mixture was stirred at rt for 30 minutes, then the mixture was cooled with ice bath. The solution of Et_3Al in hexane (0.75 mmol, 0.75 mL, 1.0 M) was added dropwise. To the colorless solution was added **3f** (69.6 mg, 61 µL, d = 1.15, 0.25 mmol) at once. The reaction was carried out at rt and monitored by TLC. After 2.5 h, a minimal amount of sat. NH_4Cl aq. was added at 0 °C. After stirring at 0 °C for 30 minutes, the mixture was filtered through silica gel pad and washed with Et_2O and the eluent was concentrated. The crude product was purified by silica gel chromatography using hexane/EtOAc (30/1) to afford **4f** (37.5 mg, 60%): colorless oil; ^{1}H NMR (400 MHz, $CDCl_3$) δ 5.12 (d, J = 1.5 Hz, 1H), 2.40–2.22 (m, 1H), ; 2.02–1.88 (m, 2H), 1.85–1.66 (m, 3H), 1.53 (s, 3H), 1.40–1.15 (m, 5H); ^{13}C{^{1}H} NMR (100 MHz, $CDCl_3$) δ 173.1, 163.5, 124.1 (q, J = 281.4 Hz), 100.0, 53.0 (q, J = 29.3 Hz), 36.8, 29.3, 29.2, 25.8, 25.4, 17.7, 17.7; ^{19}F NMR (375 MHz, $CDCl_3$) δ –74.26; HRMS (APCI, positive) *m*/*z*: [M + H]$^+$ calcd for $C_{12}H_{16}F_3O_2^+$ 249.1097; found 249.1085; $[\alpha]^{26.0}_D$ (92% ee) = +2.83 (*c* 0.99, $CHCl_3$); HPLC (CHIRALCEL OD-H, Daicel, 4.6 x 250 mm, hexane/^{i}PrOH = 99.9/0.1, 0.5 mL/min, 254 nm), t_r (major) = 11.0 min, t_r (minor) = 12.1 min.

Chiral HPLC Chart (racemic) 4f

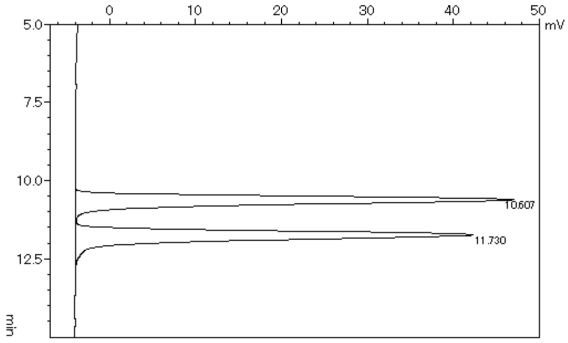

Peak#	Ret. Time	Area	Height	Area %
1	10.607	897074	51081	49.848
2	11.730	902551	46209	50.152
Total		1799626	97289	100.000

Chiral HPLC Chart (92% ee) 4f

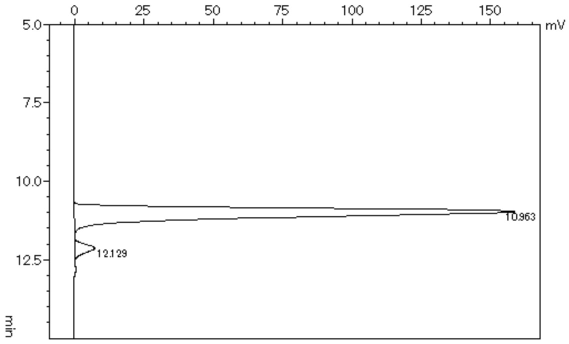

Peak#	Ret. Time	Area	Height	Area %
1	10.963	3033197	159131	95.901
2	12.129	129642	7147	4.099
Total		3162840	166277	100.000

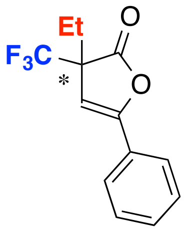

(–)-3-ethyl-5-phenyl-3-(trifluoromethyl)furan-2(3*H*)-one (**4h**): CuCl (1.2 mg, 0.0125 mmol, 5 mol%) and **SP** (7.8 mg, 0.0125 mmol, 5 mol%) were dissolved in THF (5.0 mL), and the mixture was stirred at rt for 30 minutes, then the mixture was cooled with ice bath. The solution of Et_3Al in hexane (0.75 mmol, 0.75 mL, 1.0 M) was added dropwise. To the colorless solution was added **3a** (68.0 mg, 55 µL, d = 1.23, 0.25 mmol) at once. The reaction was carried out at rt and monitored by TLC. After 2 h, a minimal amount of sat. NH_4Cl aq. was added at 0 °C. After stirring at 0 °C for 30 minutes, the mixtre was filtered through silica gel pad and washed with Et_2O and the eluent was concentrated. The crude product was purified by silica gel chromatography using hexane/EtOAc (20/1) to afford **4h** (36.8 mg, 57%): white solid, mp 48-49 °C; ^{1}H NMR (400 MHz, $CDCl_3$) δ 7.70–7.62 (m, 2H), 7.49–7.41 (m, 3H), 5.74 (s, 1H), 2.22 (dq, J = 13.6, 7.5 Hz, 1H), 2.03 (dq, J = 13.6, 7.5 Hz, 1H), 0.95 (t, J = 7.5 Hz, 3H); ^{13}C{^{1}H} NMR (100 MHz, $CDCl_3$) δ 171.8, 156.0, 130.7, 128.9, 127.0, 125.3, 124.0 (q, J = 281.9 Hz), 98.0, 59.4 (q, J =27.9 Hz), 24.5, 8.3; ^{19}F NMR (375 MHz, $CDCl_3$) δ –71.92; HRMS (APCI, positive) m/z: $[M + H]^+$ calcd for $C_{13}H_{12}F_3O_2^+$ 257.0784; found 257.0775; $[\alpha]^{29.0}_D$ (84% ee) = –21.50 (c 1.06, $CHCl_3$); HPLC (CHIRALPAK IA, Daicel, 4.6 x 250 mm, hexane/^{i}PrOH = 99.5/0.5, 0.5 mL/min, 254 nm), t_r (minor) = 11.2 min, t_r (major) = 11.8 min.

Chiral HPLC Chart (racemic) 4h

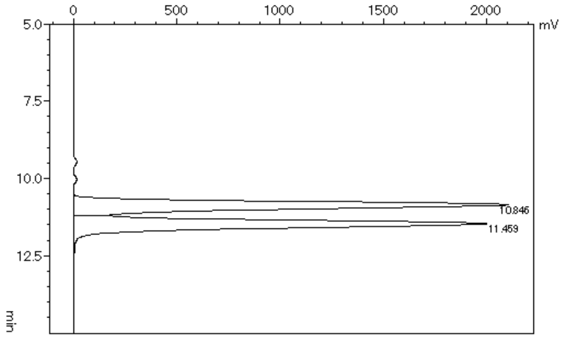

Peak#	Ret. Time	Area	Height	Area %
1	10.846	35461655	2107819	50.298
2	11.459	35040902	2001412	49.702
Total		70502557	4109231	100.000

Chiral HPLC Chart (84% ee) 4h

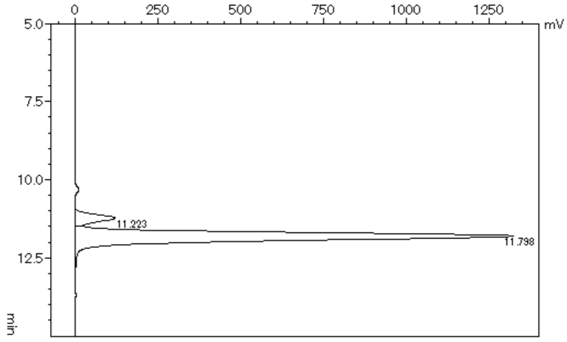

Peak#	Ret. Time	Area	Height	Area %
1	11.223	1999556	121162	7.988
2	11.798	23031763	1324488	92.012
Total		25031319	1445650	100.000

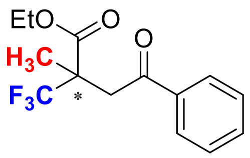

(+)-ethyl 2-methyl-4-oxo-4-phenyl-2-(trifluoromethyl)butanoate (5a): CuCl (1.2 mg, 0.0125 mmol, 5 mol%) and **SP** (7.8 mg, 0.0125 mmol, 5 mol%) were dissolved in THF (5.0 mL), and the mixture was stirred at rt for 30 minutes, then the mixture was cooled at –20 °C. The solution of Me_3Al in hexane (0.75 mmol, 0.54 mL, 1.4 M) was added dropwise. To the colorless solution was added **3a** (68.0 mg, 55 µL, d = 1.23, 0.25 mmol) at once. The reaction was carried out at rt and monitored by TLC. After 6 h, a minimal amount of sat. NH_4Cl aq. was added at –20 °C. After stirring at –20 °C for 1 hour, the mixture was filtered through silica gel pad and washed with Et_2O and the eluent was concentrated. The crude product was purified by silica gel chromatography using hexane/EtOAc (30/1) to afford **5a** (47.3 mg, 66%): colorless oil; ^{1}H NMR (400 MHz, $CDCl_3$) δ 7.97–7.91 (m, 2H), 7.60 (tt, J = 7.5, 1.5 Hz, 1H), 7.52–7.45 (m, 2H), 4.24 (q, J = 7.1 Hz, 2H), 3.90 (d, J = 17.9 Hz, 1H), 3.30 (d, J = 17.9 Hz, 1H), 1.59 (s, 3H), 1.25 (t, J = 7.1 Hz, 3H); ^{13}C{^{1}H} NMR (100 MHz, $CDCl_3$) δ 195.1, 168.6, 136.3, 133.6, 128.7, 127.9, 126.1 (q, J = 283.8 Hz), 62.0, 50.1 (q, J = 24.9 Hz), 41.4, 16.4, 13.8; ^{19}F NMR (375 MHz, $CDCl_3$) δ –74.37; HRMS (APCI, positive) m/z: $[M + H]^+$ calcd for $C_{14}H_{16}F_3O_3^+$ 289.1046; found 289.1060; $[\alpha]^{27.0}_D$ (92% ee) = +35.82 (c 1.39, $CHCl_3$); HPLC (CHIRALPAK AD, Daicel, 4.6 x 250 mm, hexane/^{i}PrOH = 99/1, 0.5 mL/min, 254 nm), t_r (minor) = 16.8 min, t_r (major) = 20.4 min.

Chiral HPLC Chart (racemic) 5a

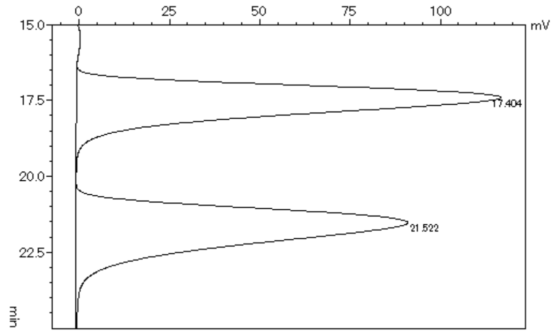

Peak#	Ret. Time	Area	Height	Area %
1	17.404	7391165	117531	50.307
2	21.522	7301058	91817	49.693
Total		14692222	209347	100.000

Chiral HPLC Chart (92% ee) 5a

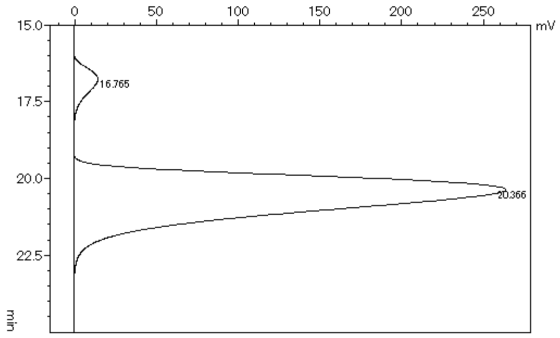

Peak#	Ret. Time	Area	Height	Area %
1	16.765	875086	14801	4.023
2	20.366	20879303	264535	95.977
Total		21754390	279336	100.000

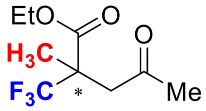

(+)-ethyl 2-methyl-4-oxo-2-(trifluoromethyl)pentanoate (5g): CuCl (1.2 mg, 0.0125 mmol, 5 mol%) and **SP** (7.8 mg, 0.0125 mmol, 5 mol%) were dissolved in THF (5.0 mL), and the mixture was stirred at rt for 30 minutes, then the mixture was cooled at –20 °C. The solution of Me_3Al in hexane (0.75 mmol, 0.54 mL, 1.4 M) was added dropwise. To the colorless solution was added **3g** (52.5 mg, 43 µL, *d* = 1.21, 0.25 mmol) at once. The reaction was carried out at rt and monitored by TLC. After 6 h, a minimal amount of sat. NH_4Cl aq. was added at –20 °C. After stirring at –20 °C for 1 hour, the mixture was filtered through silica gel pad and washed with Et_2O and the eluent was concentrated. The crude product was purified by silica gel chromatography using hexane/EtOAc (5/1) to afford **5g** (31.5 mg, 58%): colorless oil; ^1H NMR (400 MHz, $CDCl_3$) δ 4.26–4.17 (m, 2H), 3.37 (d, *J* = 18.0 Hz, 1H), 2.71 (d, *J* = 18.0 Hz, 1H), 2.18 (s, 3H), 1.50 (s, 3H), 1.26 (t, *J* = 7.1 Hz, 3H); $^{13}C\{^1H\}$ NMR (100 MHz, $CDCl_3$) δ 203.5, 168.5, 125.8 (q, *J* = 283.4 Hz), 62.0, 49.8 (q, *J* = 24.9 Hz), 45.6, 30.2, 16.2, 13.8; ^{19}F NMR (375 MHz, $CDCl_3$) δ –74.69; HRMS (APCI, positive) *m*/*z*: $[M + H]^+$ calcd for $C_9H_{14}F_3O_3^+$ 227.0890; found 227.0877; $[\alpha]^{28.0}_D$ (72% ee) = +14.01 (*c* 1.30, $CHCl_3$); HPLC (CHIRALPAK IC, Daicel, 4.6 x 250 mm, hexane/^{i}PrOH = 99.5/0.5, 0.5 mL/min, 230 nm), t_r (major) = 18.2 min, t_r (minor) = 19.5 min.

Chiral HPLC Chart (racemic) 5g

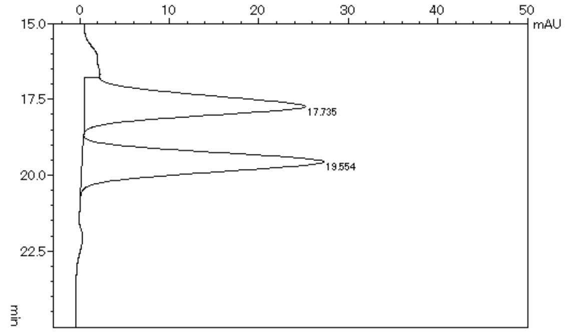

Peak#	Ret. Time	Area	Height	Area %
1	17.735	1143125	24698	49.013
2	19.554	1189178	26980	50.987
Total		2332303	51678	100.000

Chiral HPLC Chart (72% ee) 5g

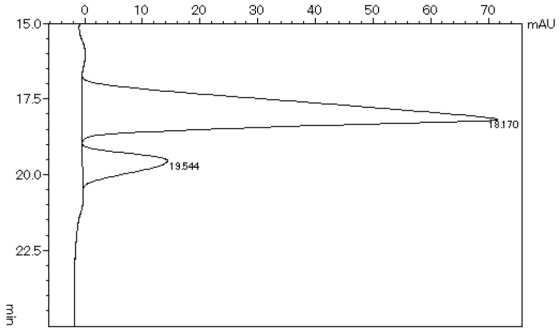

Peak#	Ret. Time	Area	Height	Area %
1	18.170	3752547	72115	85.770
2	19.544	622586	14776	14.230
Total		4375134	86890	100.000

4. X-ray Single Crystal Data for 4e (Ellipsoid contour probability level = 50%)

The crystal of **4e** was grown by the slow evaporation of its solution in chloroform and hexane at room temperature.

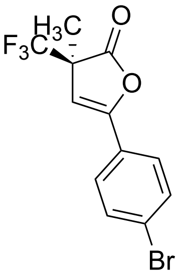

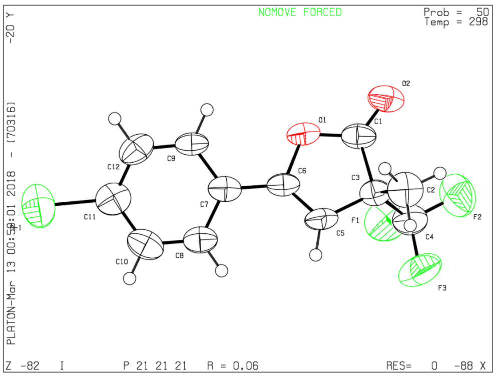

Chemical formula	$C_{12}H_8BrF_3O_2$
Formula weight	321.09
Temperature	298 K
Wavelength	0.71073 Å (MoKα)
Crystal size	0.200 x 0.300 x 0.300 mm
Crystal habit	clear colorless rectangular
Crystal system	orthorhombic
Space group	$P2_1$
Unit cell dimensions	a = 6.3102(6) Å, b = 7.0690(6) Å, c = 27.581(3) Å<br>α = 90°, β = 90°, γ = 90°
Volume	1230.30(19) $Å^3$
Z	4
Density (calculated)	1.734 g/cm^3
Absorption coefficient	3.369 mm-1
F(000)	632
Theta range for data collection	2.95 to 25.11°
Index ranges	-6<=h<=7, -7<=k<=8, -26<=l<=32
Reflections collected	7224
Independent reflections	2186 [R(int) = 0.0482]
Coverage of independent reflections	99.5%
Absorption correction	multi-scan
Data / restraints / parameters	2186 / 0 / 164
Goodness-of-fit on F^2	1.040
Final R indices (I>2σ(I))	R1 = 0.0575, wR2 = 0.1300
Final R indices (all data)	R1 = 0.0878, wR2 = 0.1456
Largest diff. peak and hole	0.495 and -0.525 $eÅ^{-3}$
Flack parameter	0.06

5. Prediction of Stereochemistry of Acyclic Product

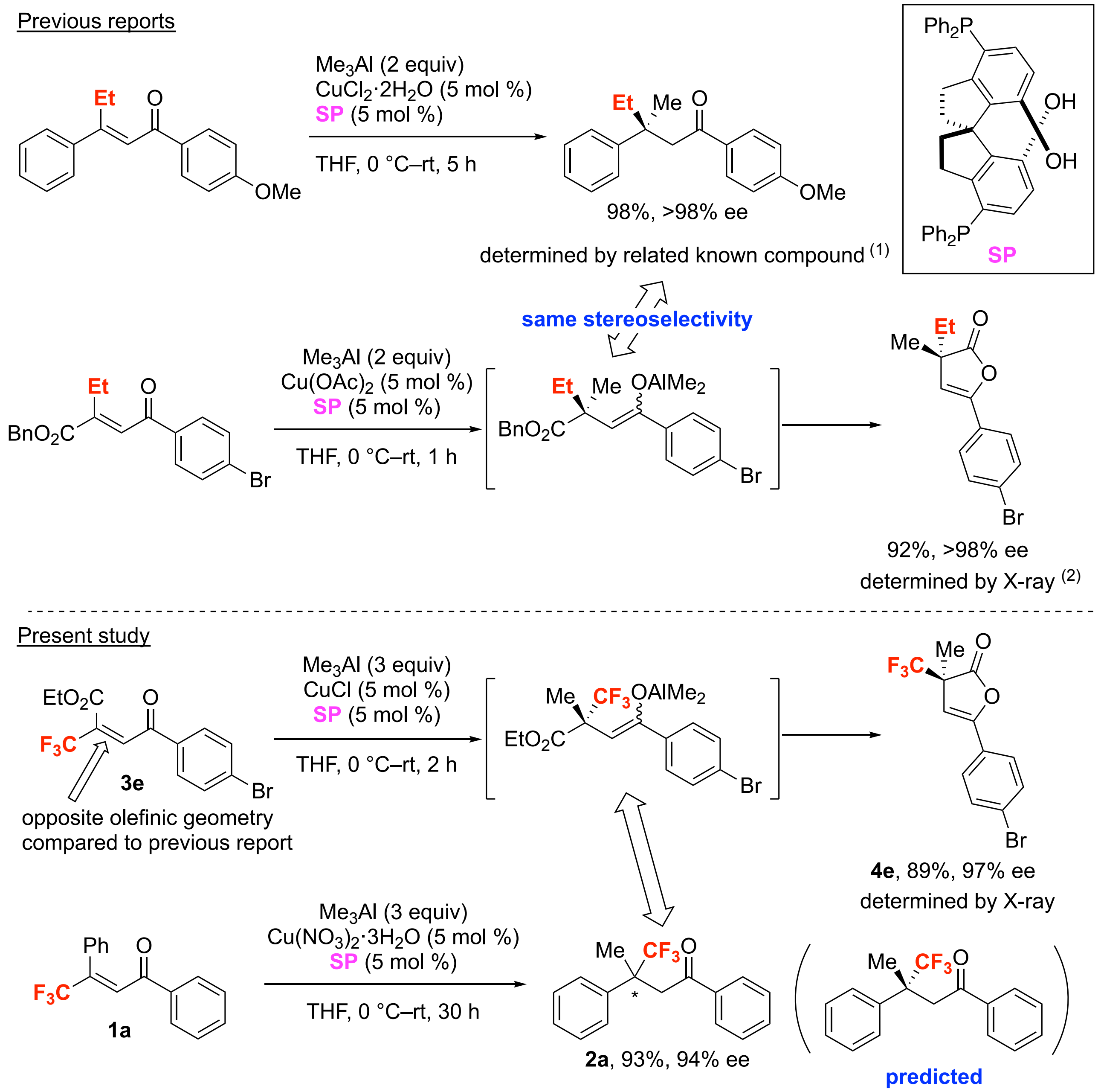

(1) Endo, K.; Hamada, D.; Yakeishi, S.; Shibata, T. *Angew. Chem. Int. Ed.* **2013**, *52*, 606–610.
(2) Endo, K.; Yakeishi, S.; Takayama, R.; Shibata, T. *Chem. Eur. J.* **2014**, *20*, 8893–8897.

We cannot determine the absolute stereochemistry of CF_3-substituted product **2** derived from enones **1**, but the absolute stereochemistry of CF_3-substituted furanones **4** derived from unsaturated ketoesters **3** was determined by single crystal X-ray diffraction analysis.

According to our previous reports (1) and (2), the Cu-catalyzed conjugate addition of Me_3Al to enones and unsaturated ketoesters takes place via the same stereoselectivity in the presence of **SP** as a ligand.

The present study for the use of CF_3-substituted unsaturated ketoester **3e** gives same stereoselectivity compared to that in (1) and (2) in the presence of **SP** as a ligand. Thus, we consider that the use of CF_3-substituted enone **1** for the synthesis of product **2** would show same stereoselectivity.

6. NMR Chart of Compounds

^{1}H NMR (400 MHz, $CDCl_3$) (*E*)-**1a** (known compound)

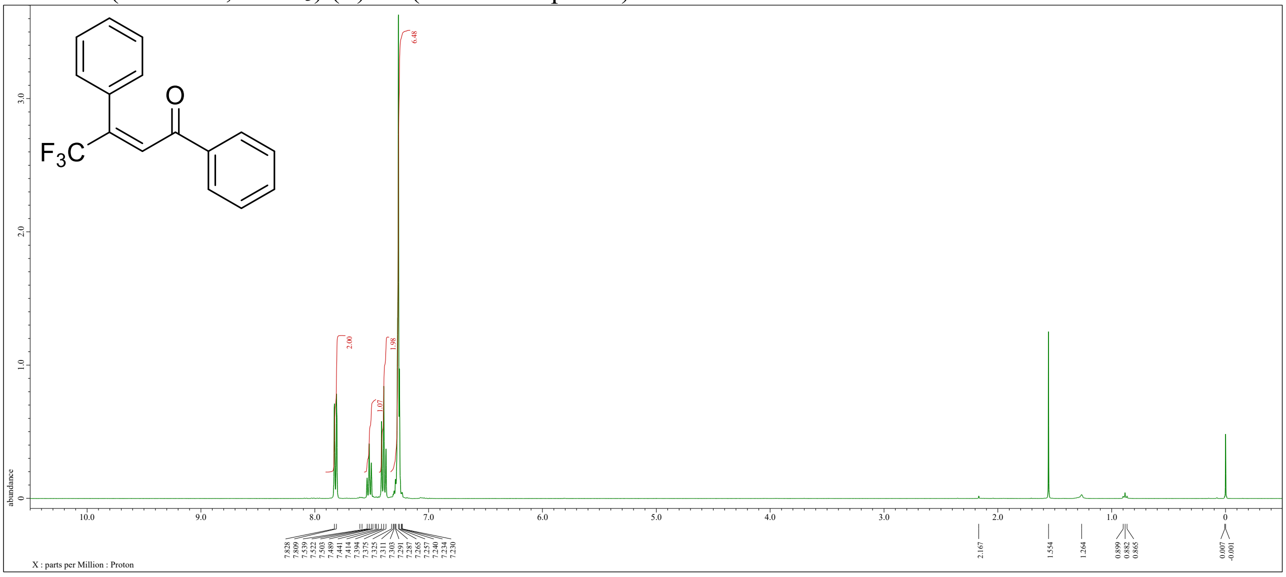

^{1}H NMR (400 MHz, $CDCl_3$) (*Z*)-**1a** (known compound)

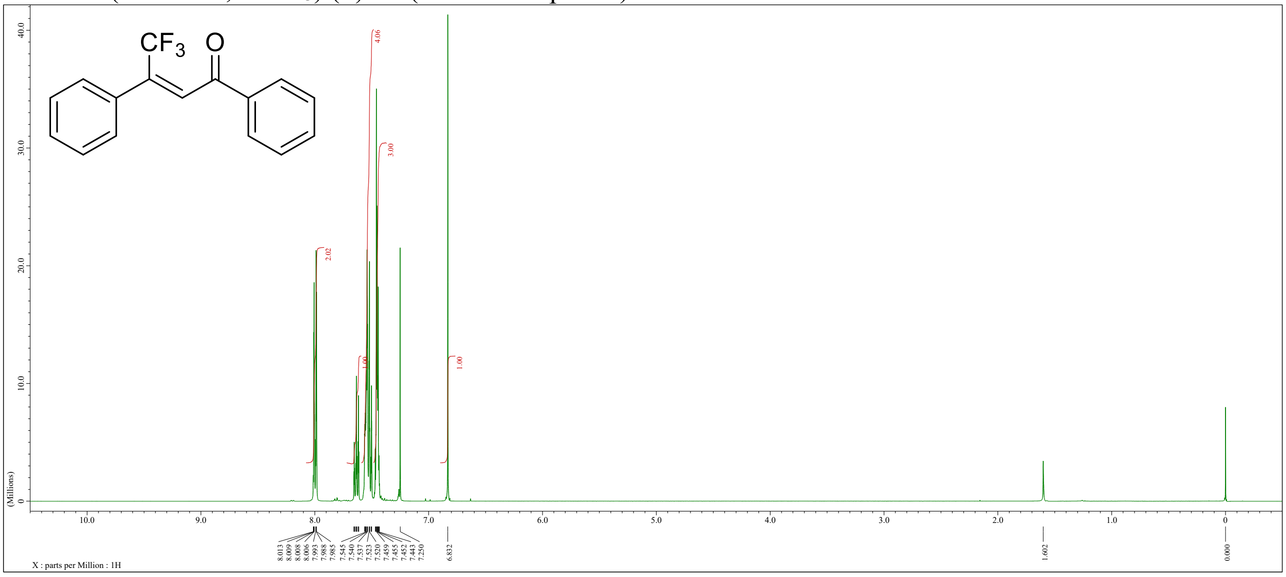

^{1}H NMR (400 MHz, $CDCl_3$) **1b** (known compound)

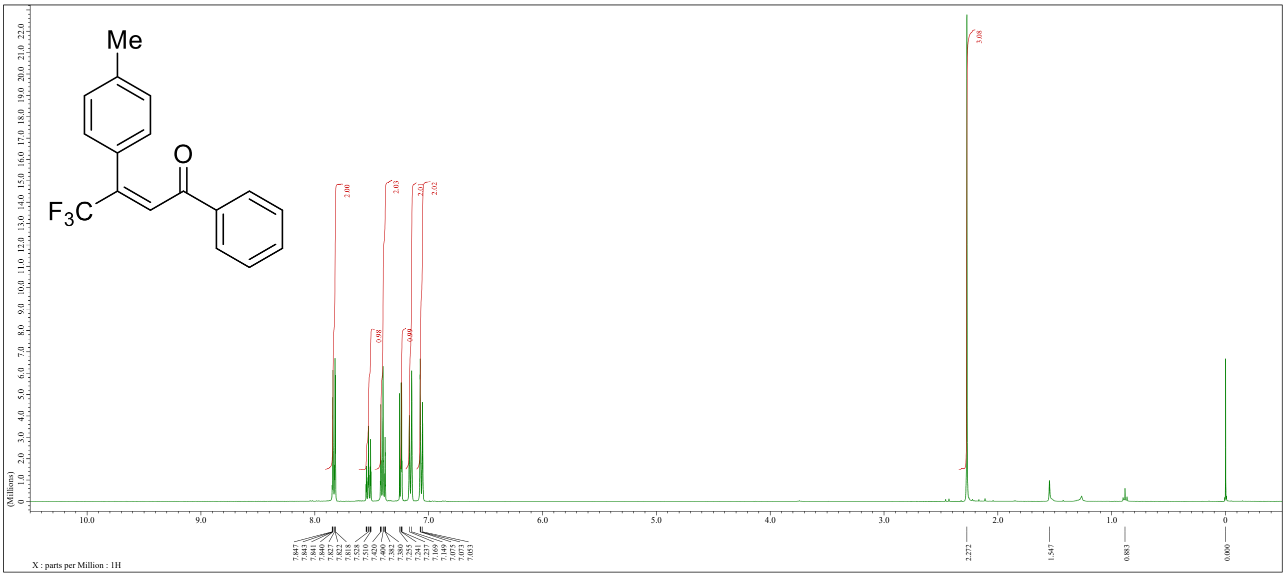

^{1}H NMR (400 MHz, $CDCl_3$) **1c**

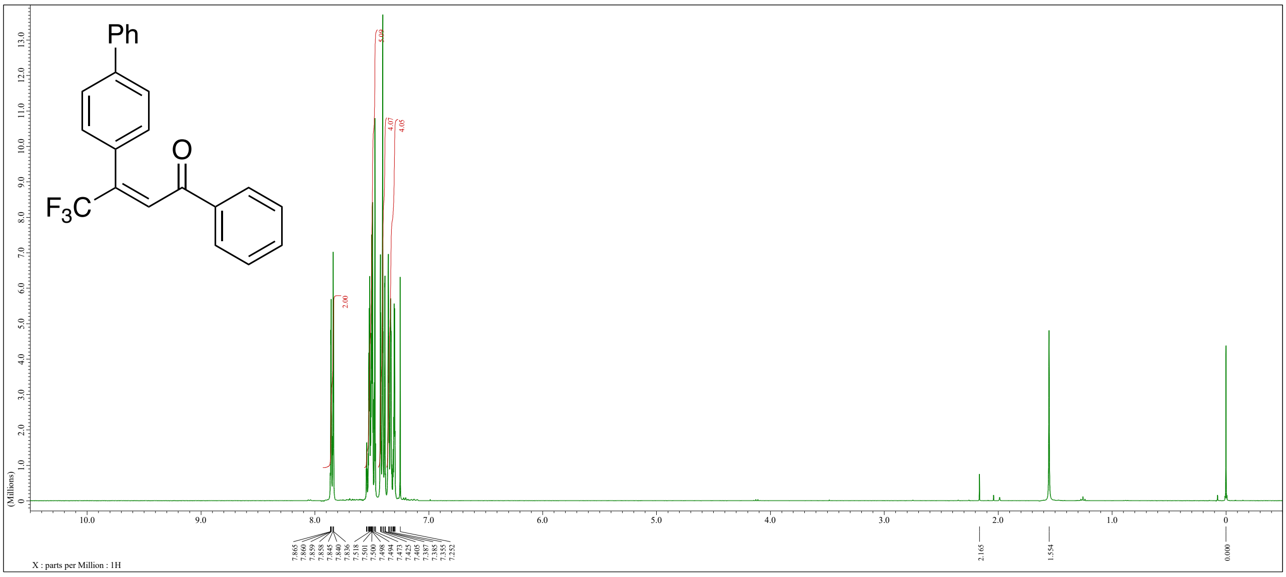

^{13}C NMR (100 MHz, $CDCl_3$) **1c**

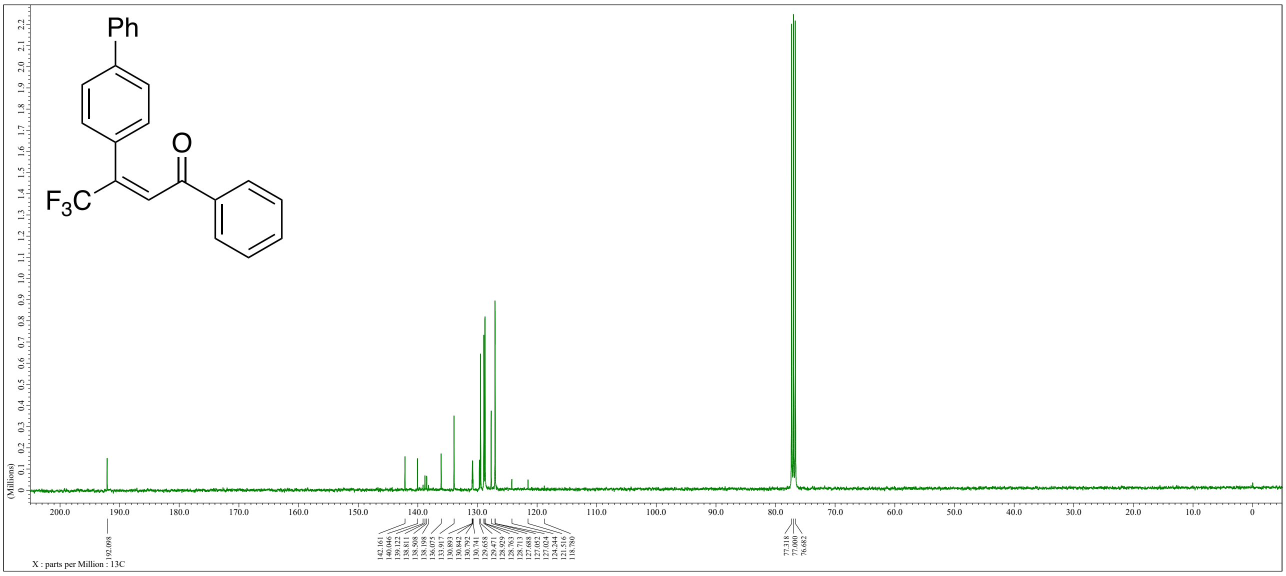

^{19}F NMR (375 MHz, $CDCl_3$) **1c**

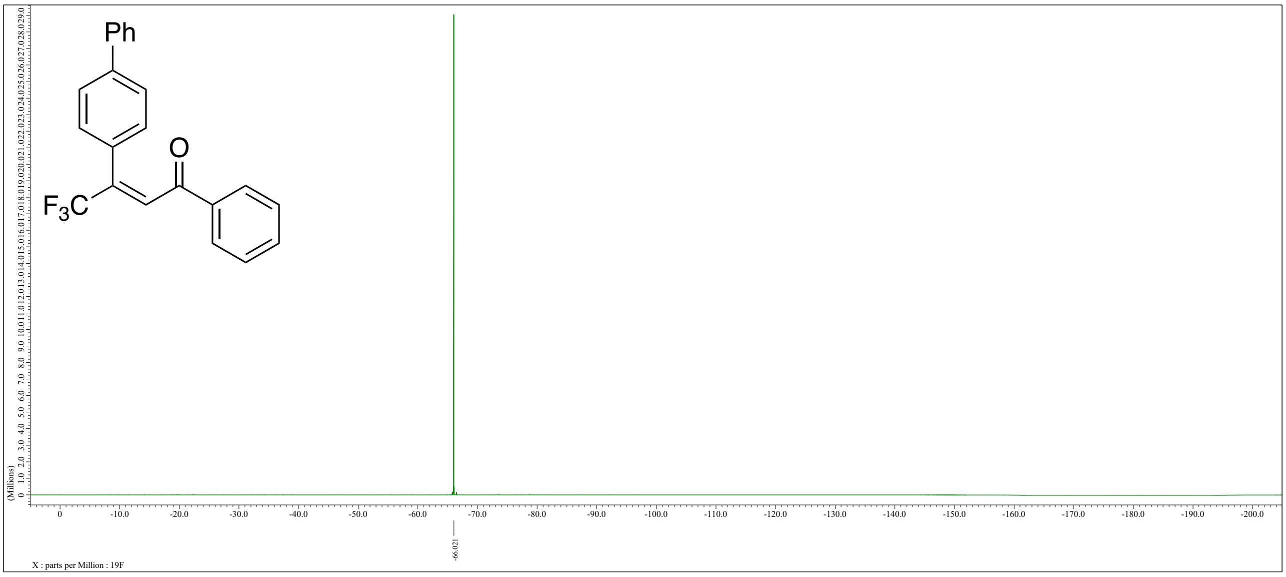

^{1}H NMR (400 MHz, $CDCl_3$) **1d** (known compound)

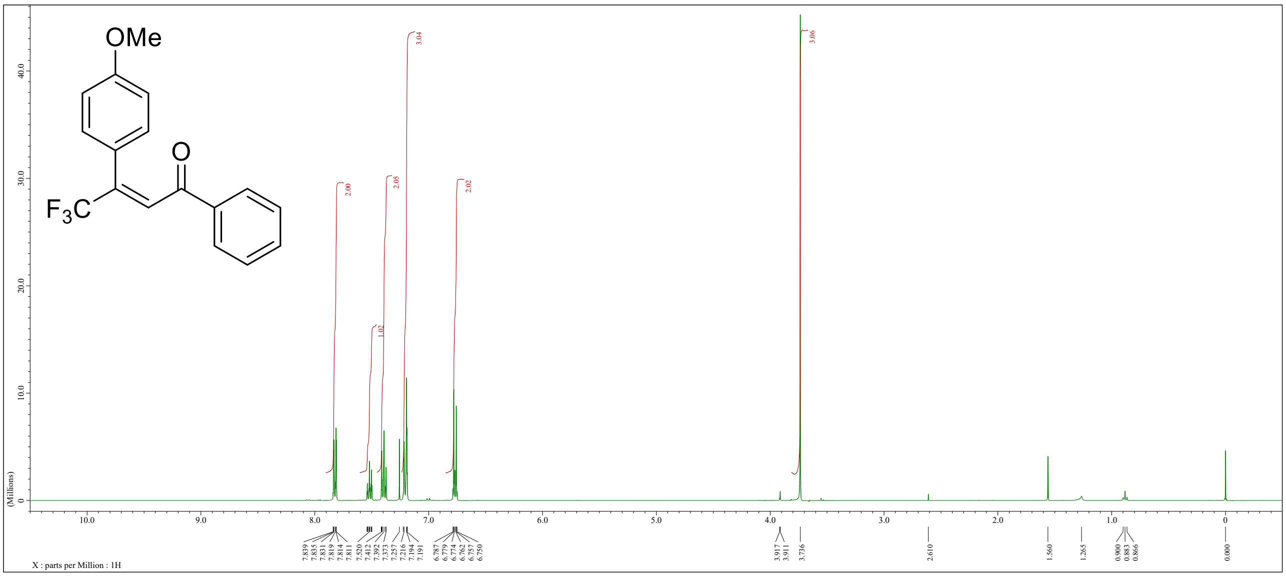

^{1}H NMR (400 MHz, $CDCl_3$) **1e** (known compound)

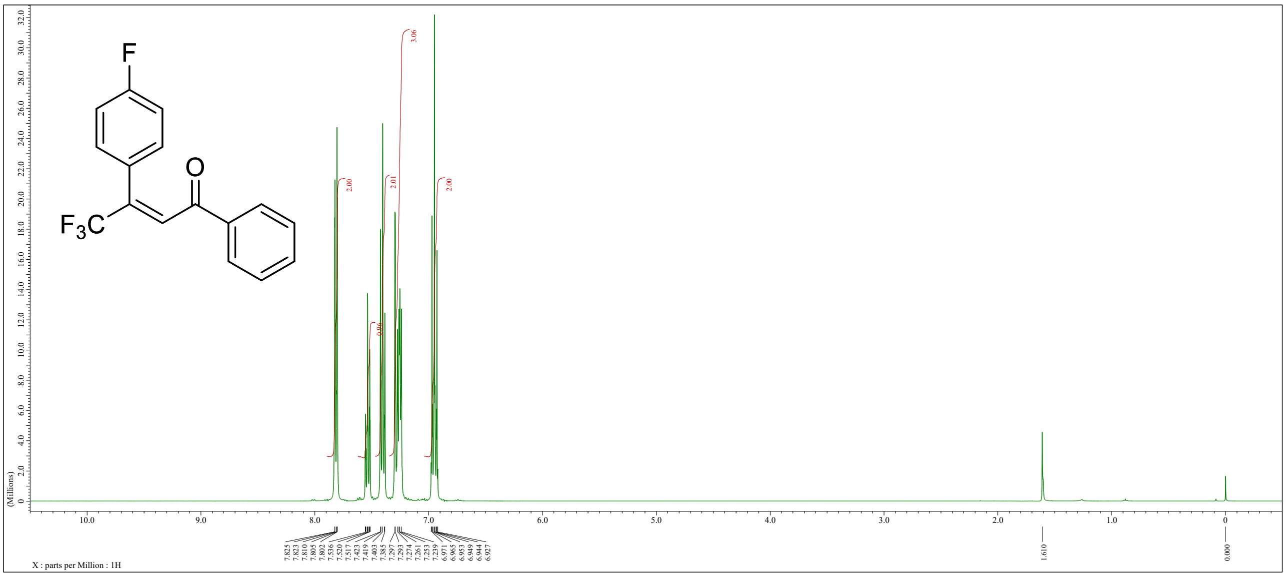

^{1}H NMR (400 MHz, $CDCl_3$) **1f**

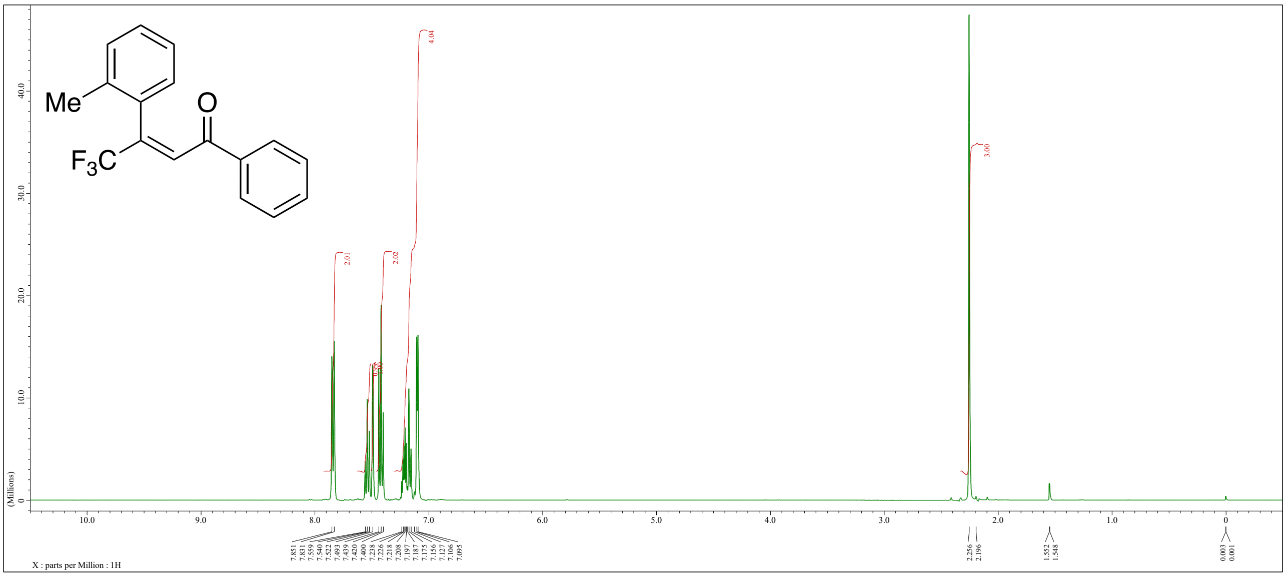

^{13}C NMR (100 MHz, $CDCl_3$) **1f**

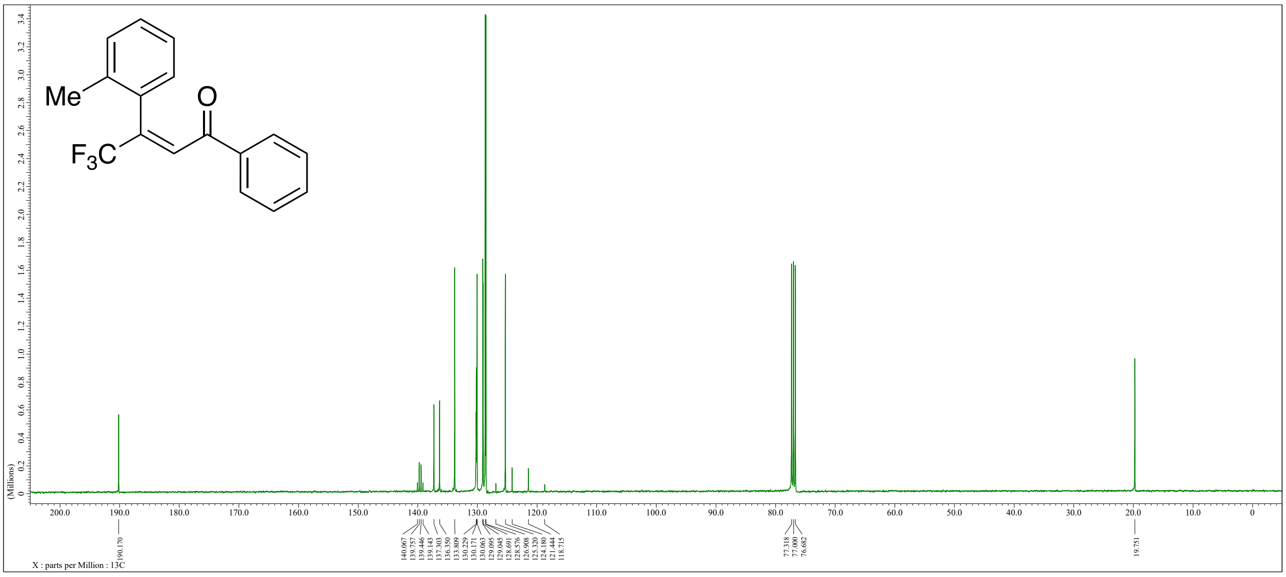

^{19}F NMR (375 MHz, $CDCl_3$) **1f**

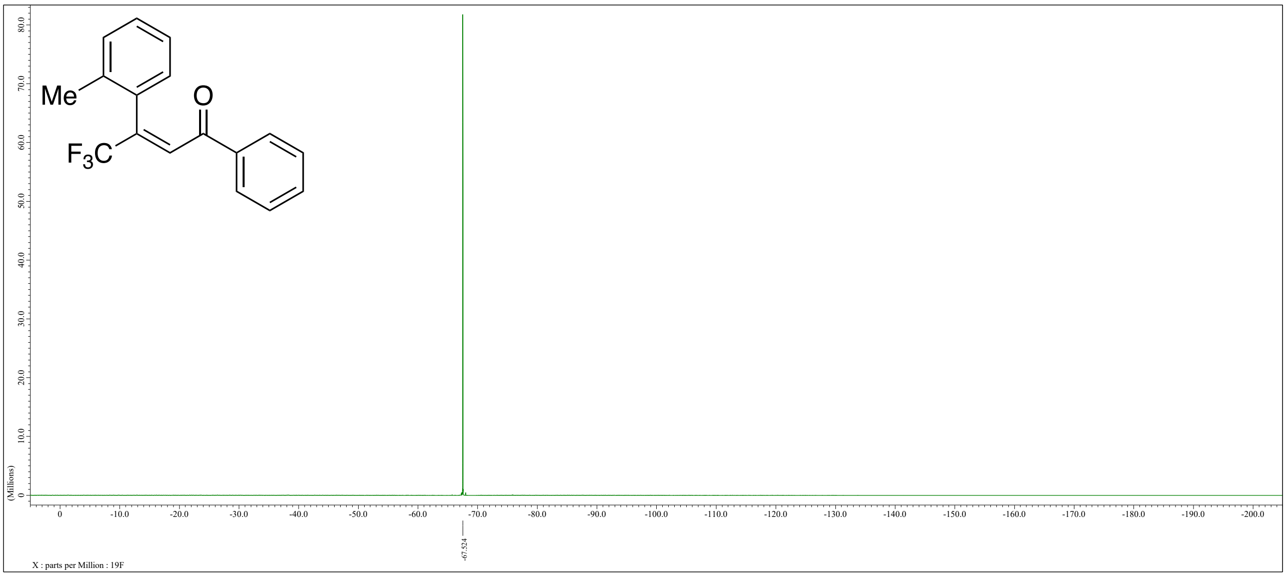

^{1}H NMR (400 MHz, $CDCl_3$) **1g** (known compound)

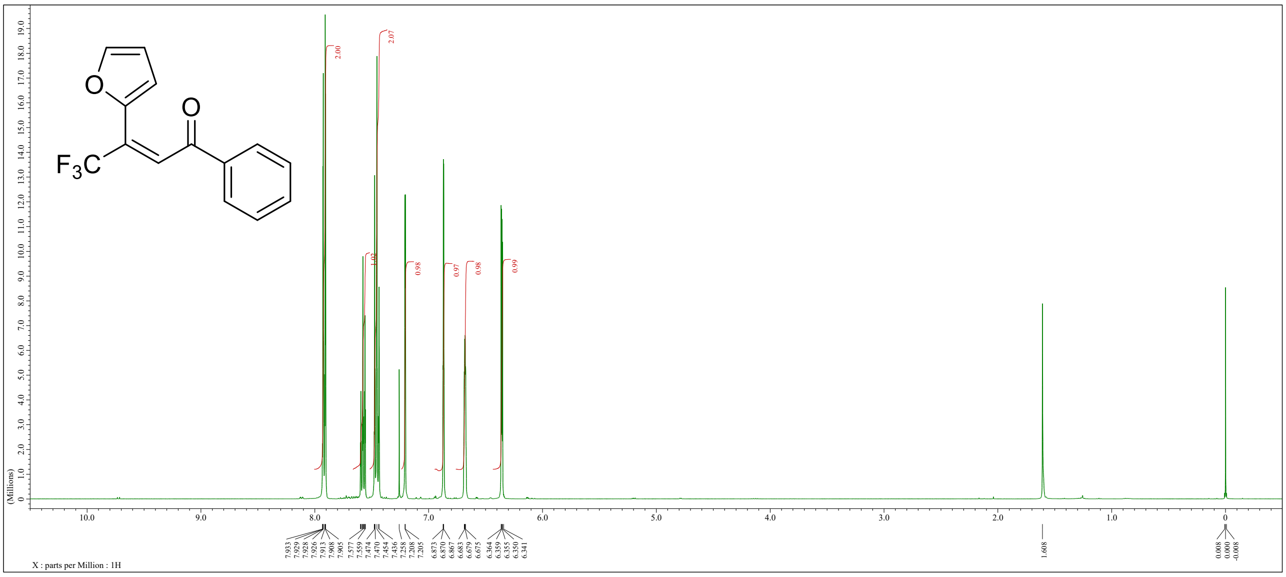

^{1}H NMR (400 MHz, $CDCl_3$) **1h** (known compound)

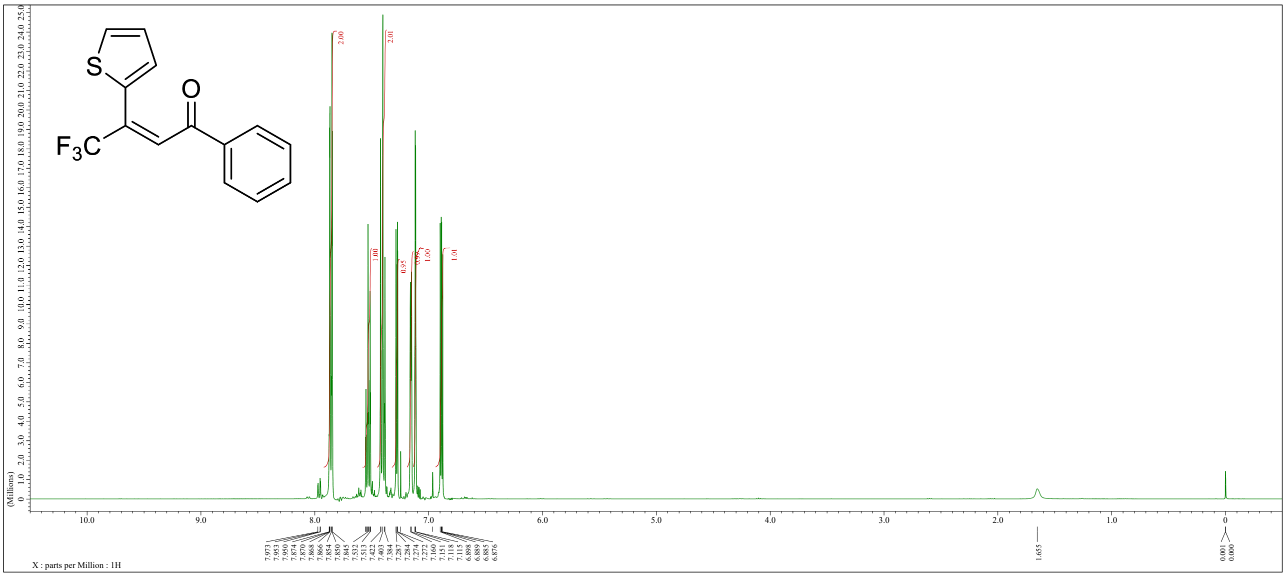

^{1}H NMR (400 MHz, $CDCl_3$) **1i** (known compound)

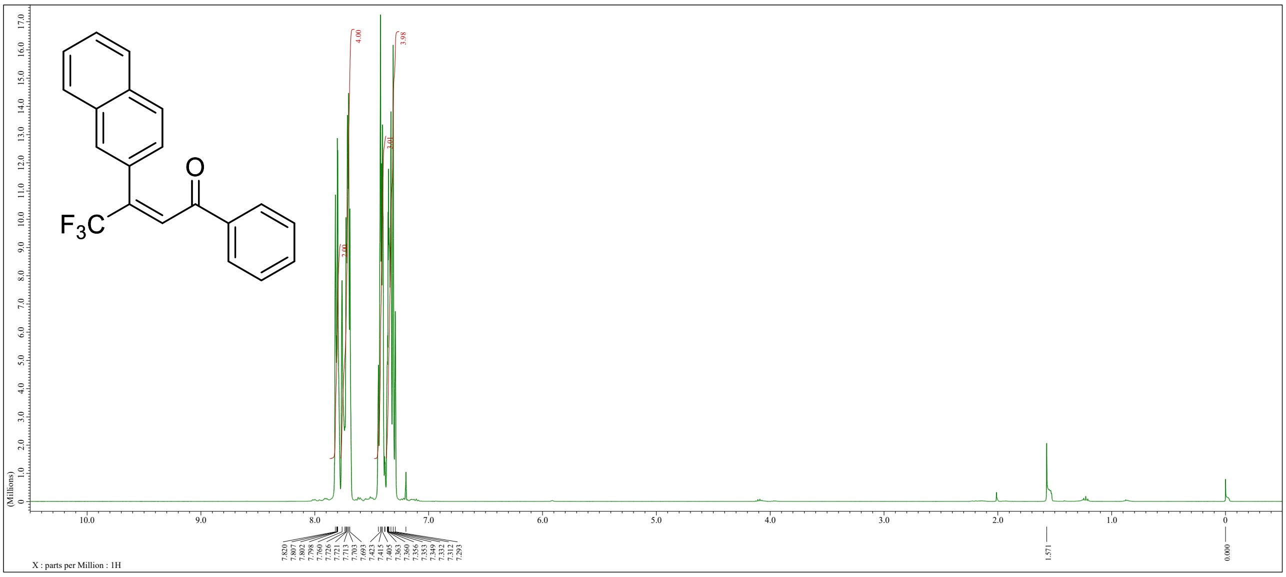

^{1}H NMR (500 MHz , $CDCl_3$) (*E*)-**3a**

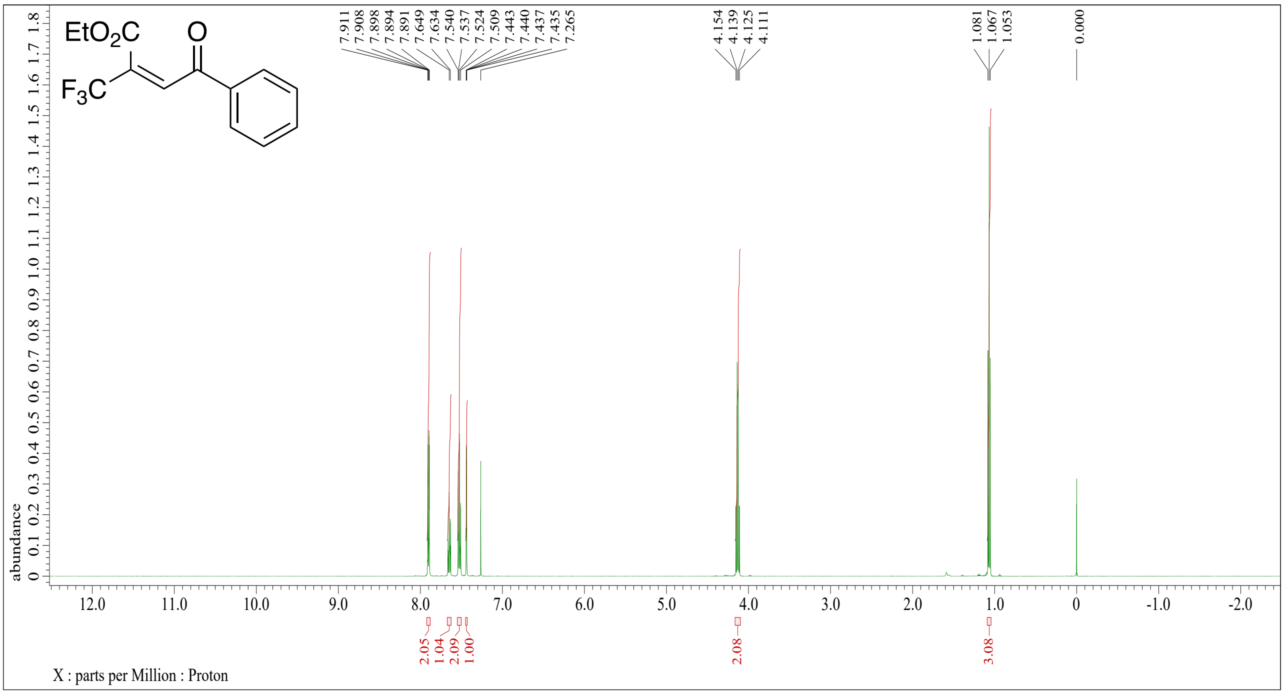

^{13}C NMR (125 MHz, $CDCl_3$) (*E*)-**3a**

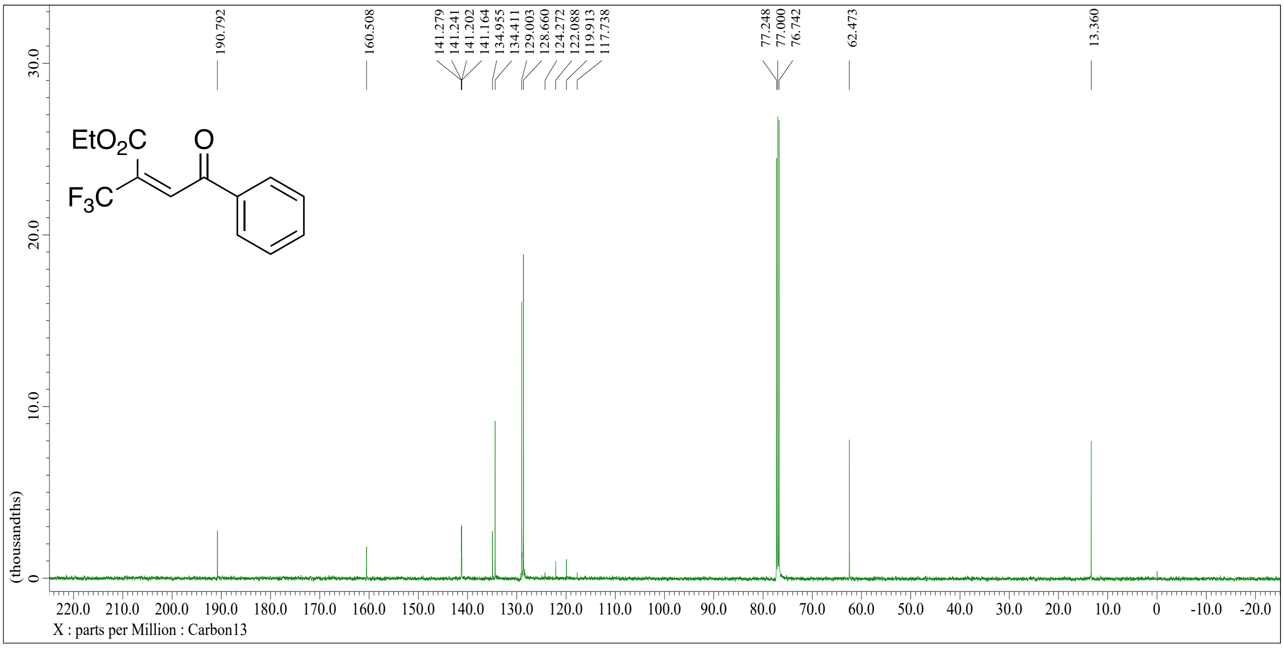

^{19}F NMR (375 MHz, $CDCl_3$) (*E*)-**3a**

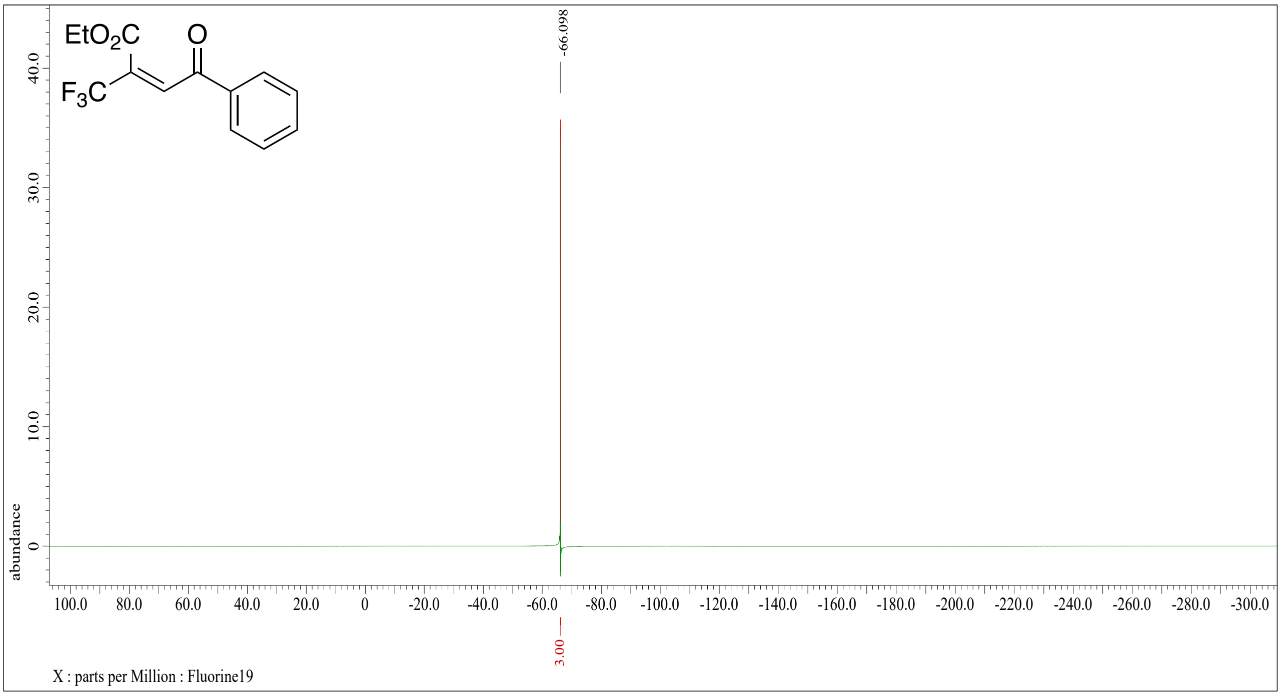

^{1}H NMR (400 MHz, $CDCl_3$) (*Z*)-**3a**

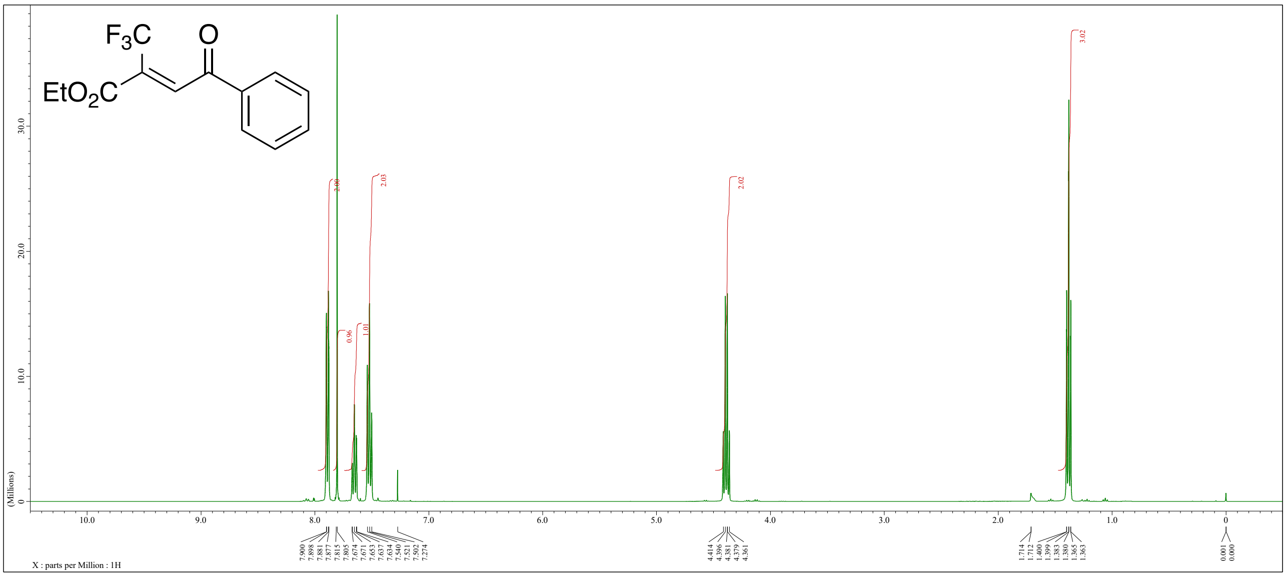

^{13}C NMR (100 MHz, $CDCl_3$) (*Z*)-**3a**

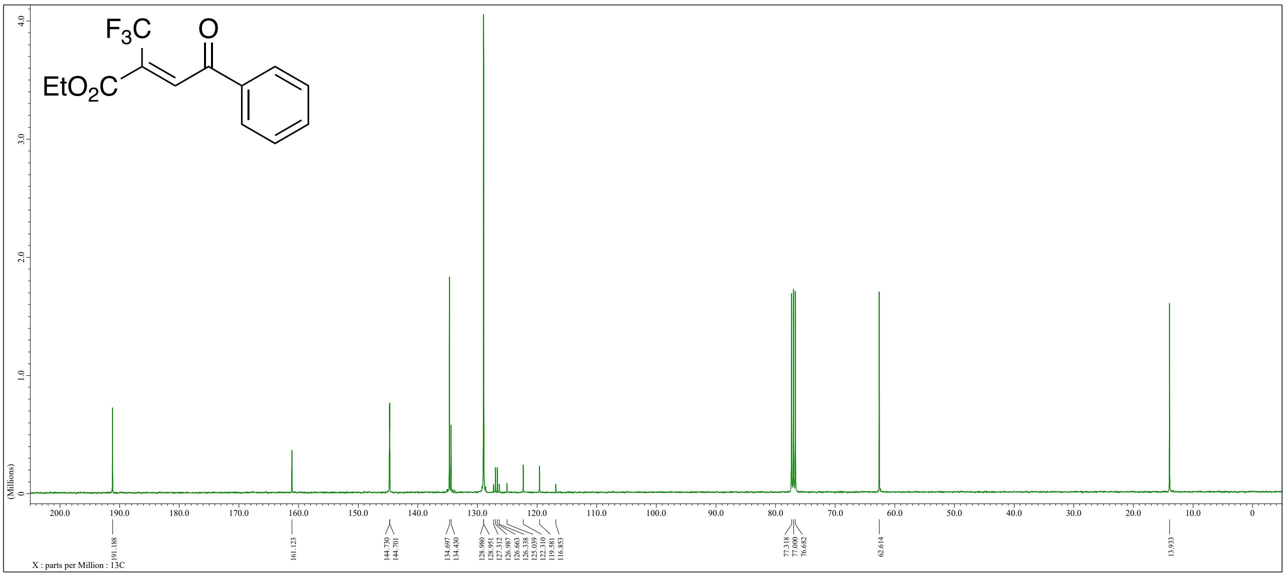

^{19}F NMR (375 MHz, $CDCl_3$) (*Z*)-**3a**

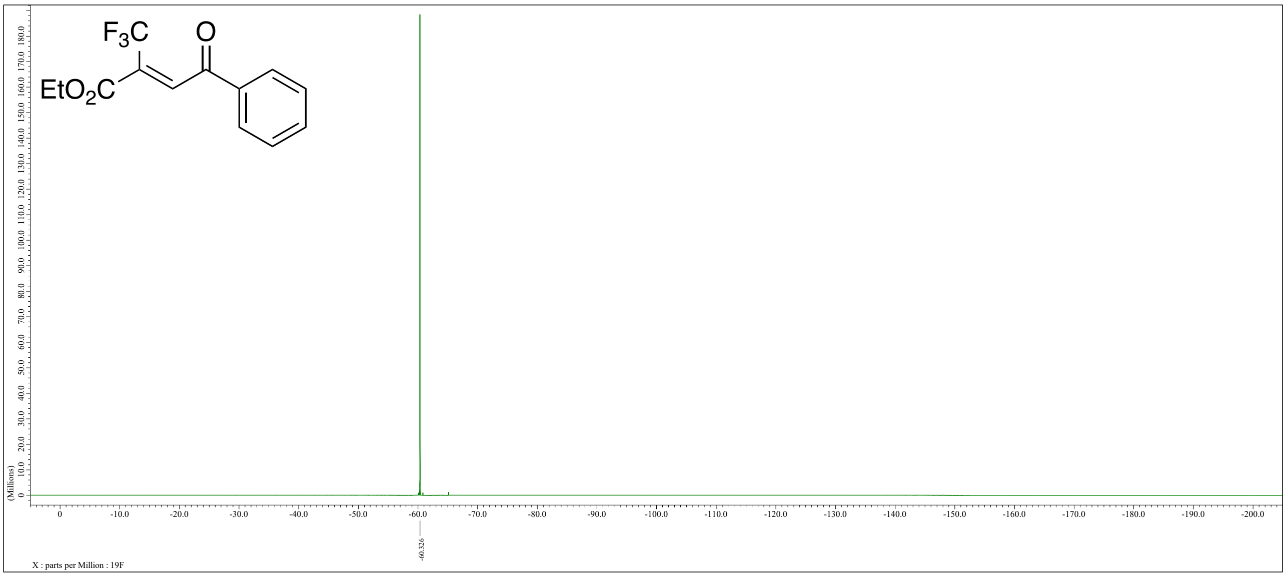

^{1}H NMR (500 MHz, $CDCl_3$) **3b**

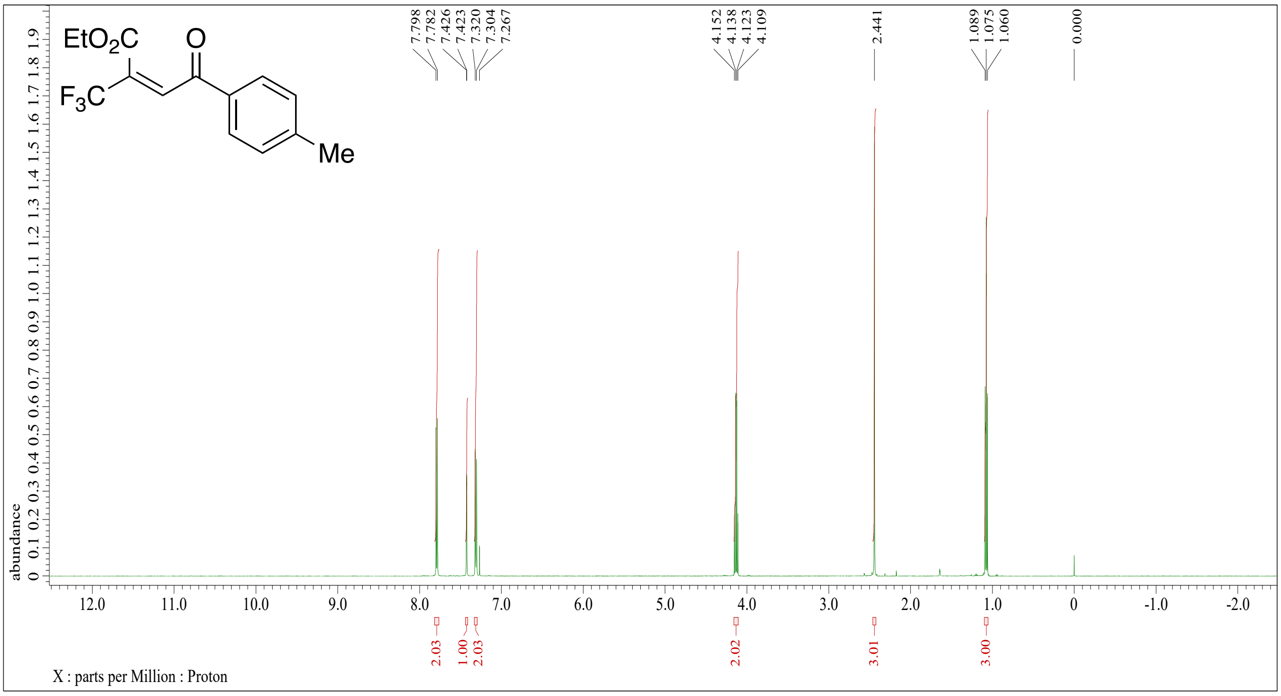

^{13}C NMR (125 MHz, $CDCl_3$) **3b**

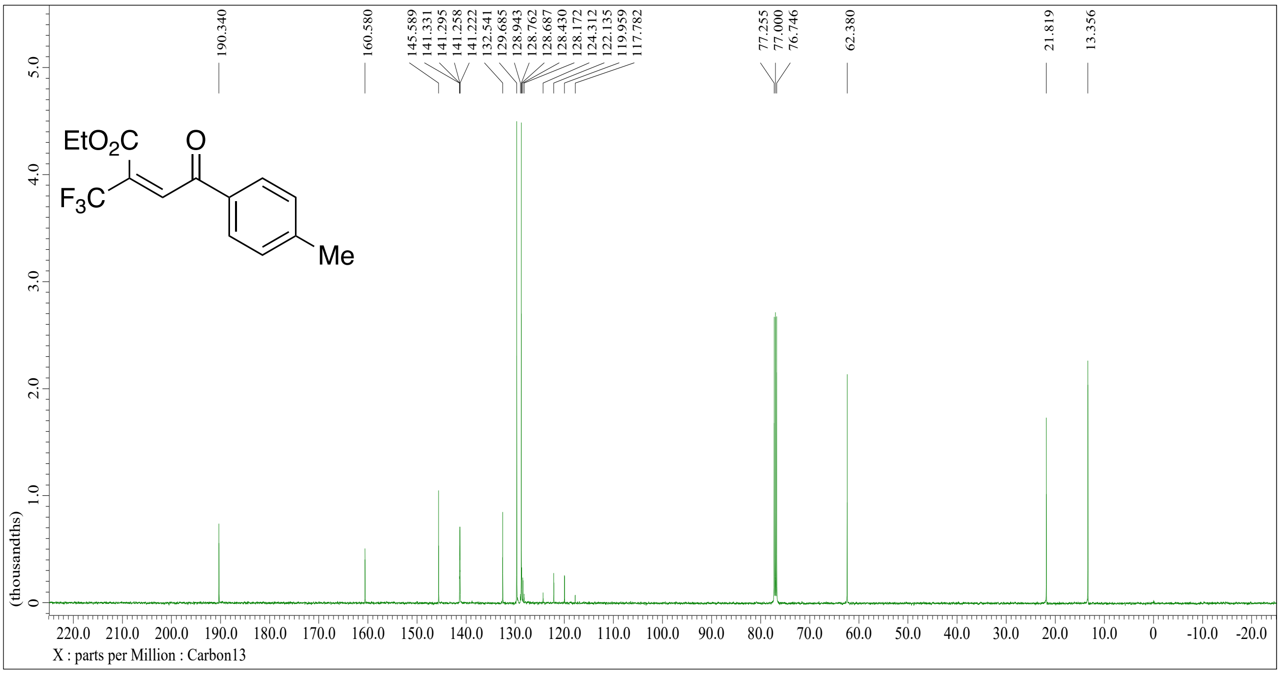

^{19}F NMR (375 MHz, $CDCl_3$) **3b**

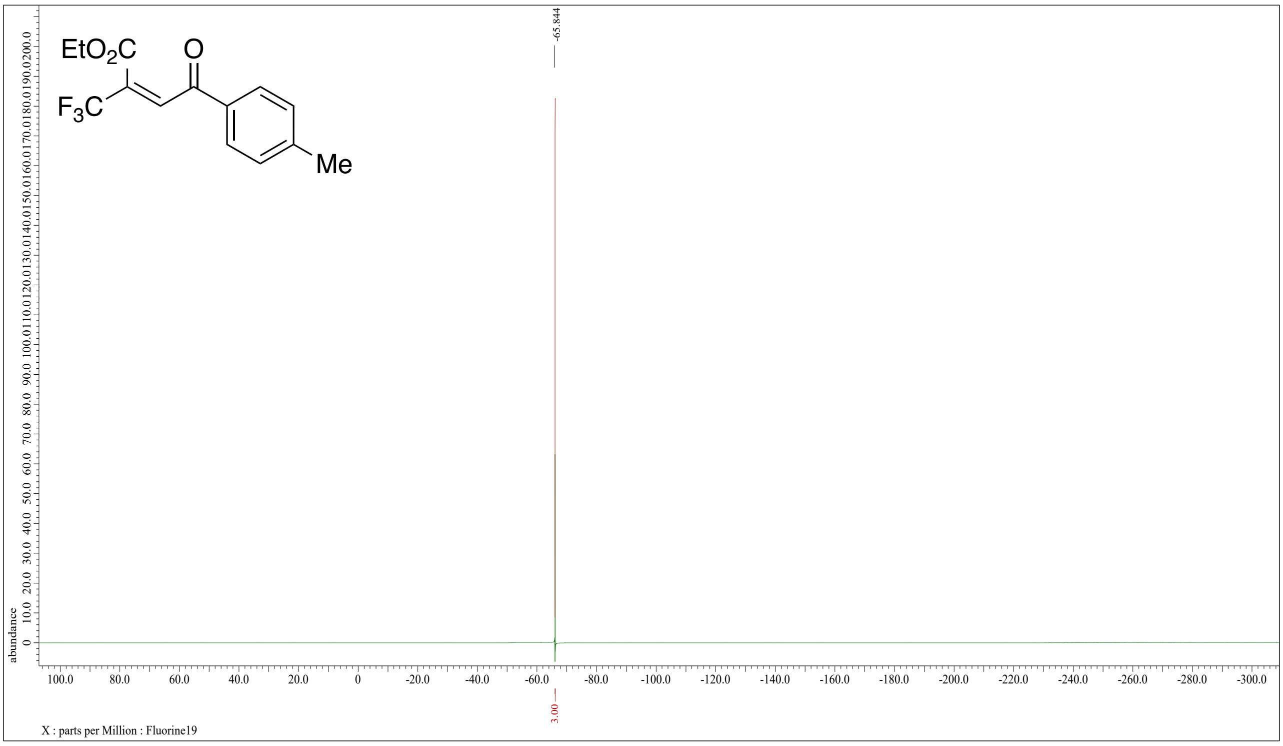

^{1}H NMR (500 MHz, $CDCl_3$) **3c**

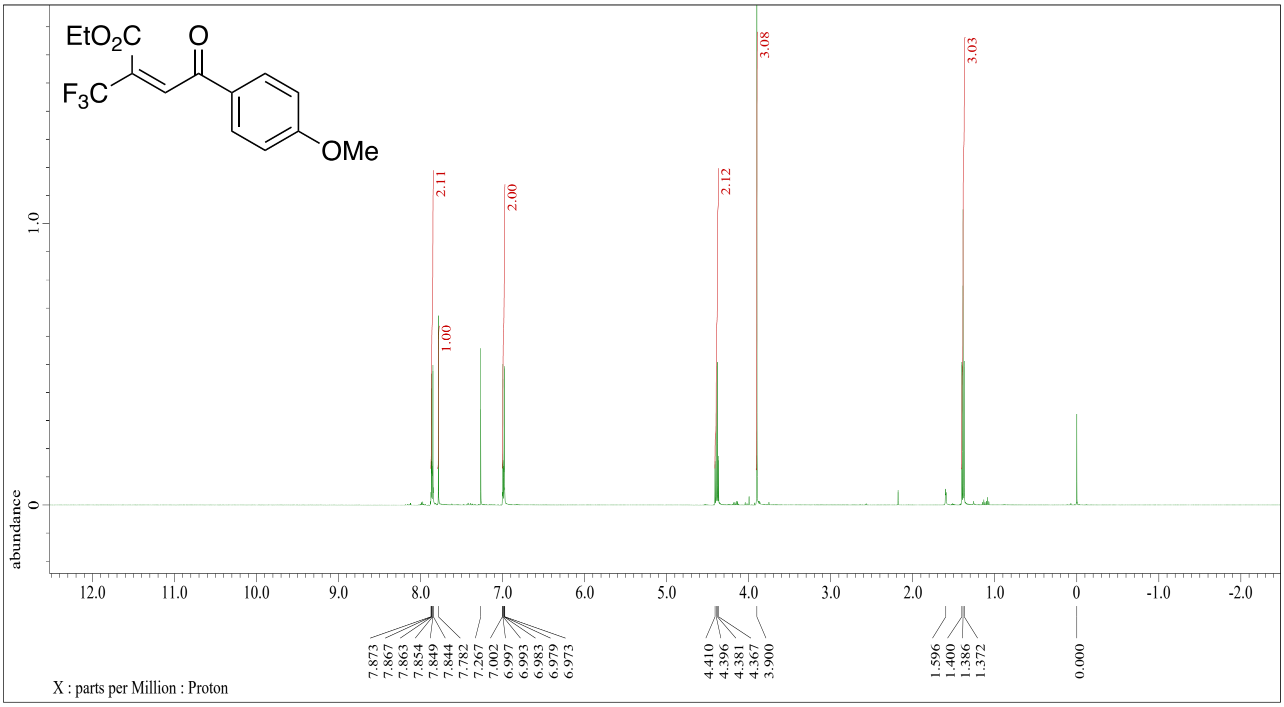

^{13}C NMR (125 MHz, $CDCl_3$) **3c**

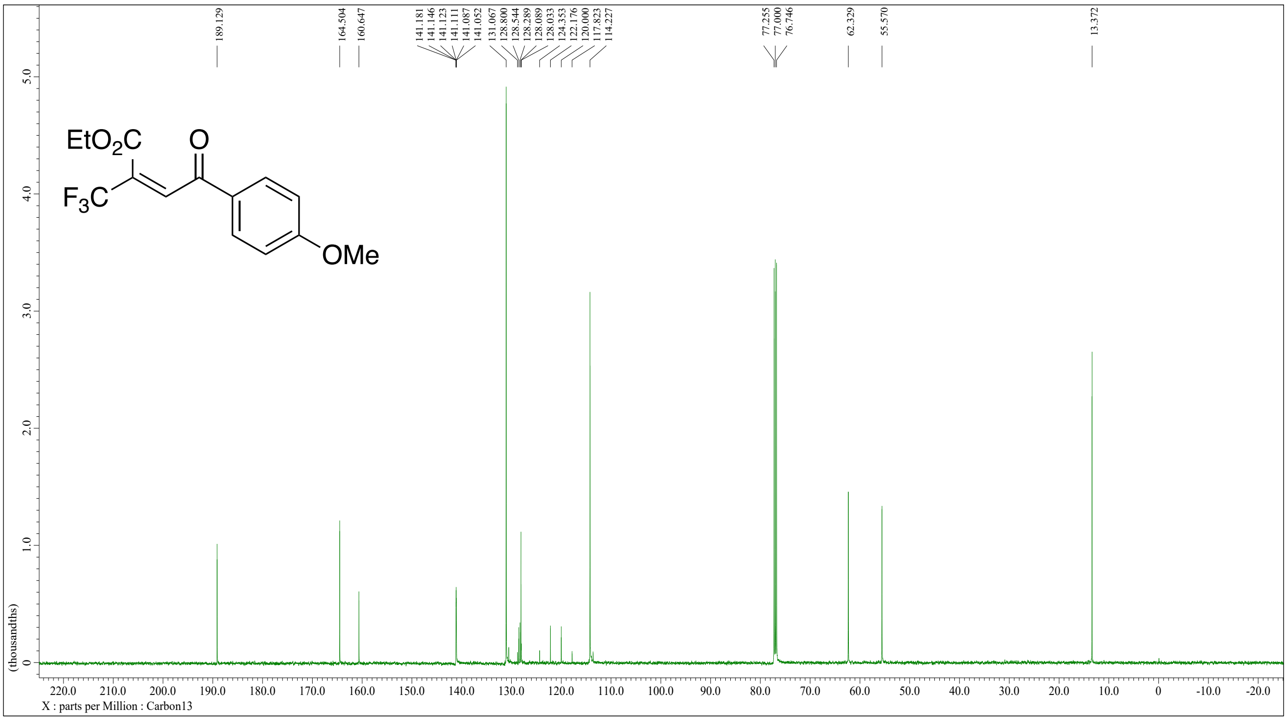

^{19}F NMR (375 MHz, $CDCl_3$) **3c**

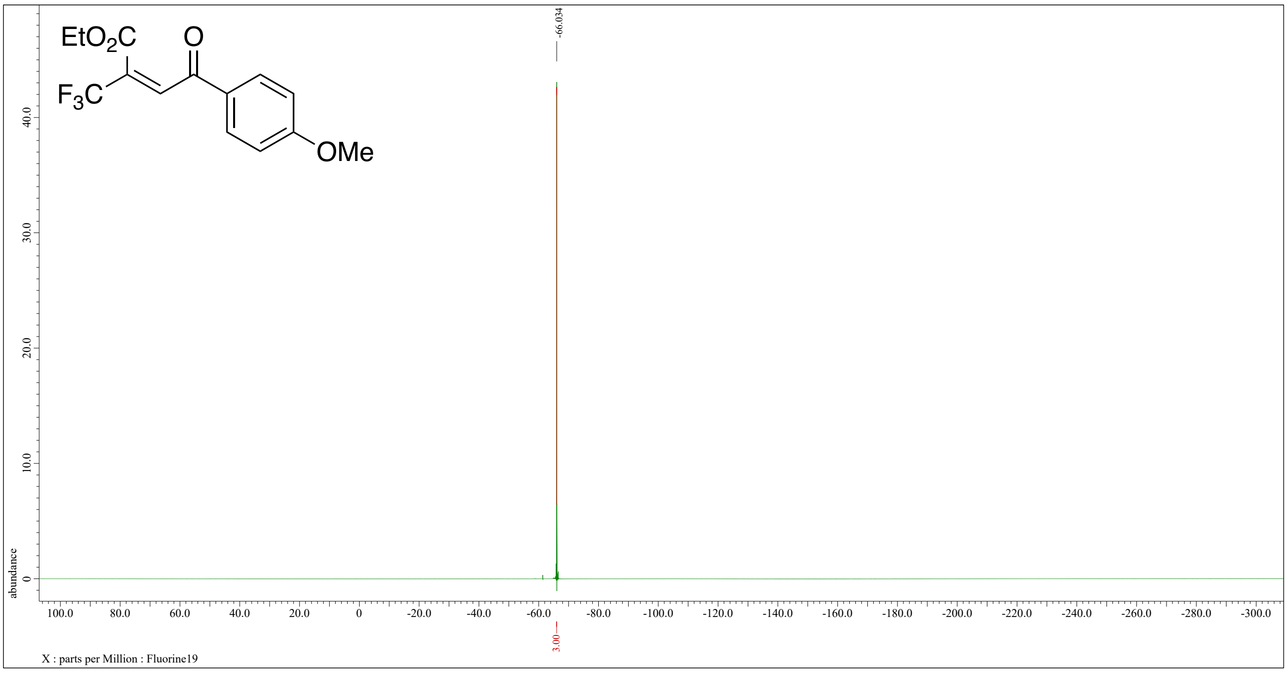

^{1}H NMR (500 MHz, $CDCl_3$) **3d**

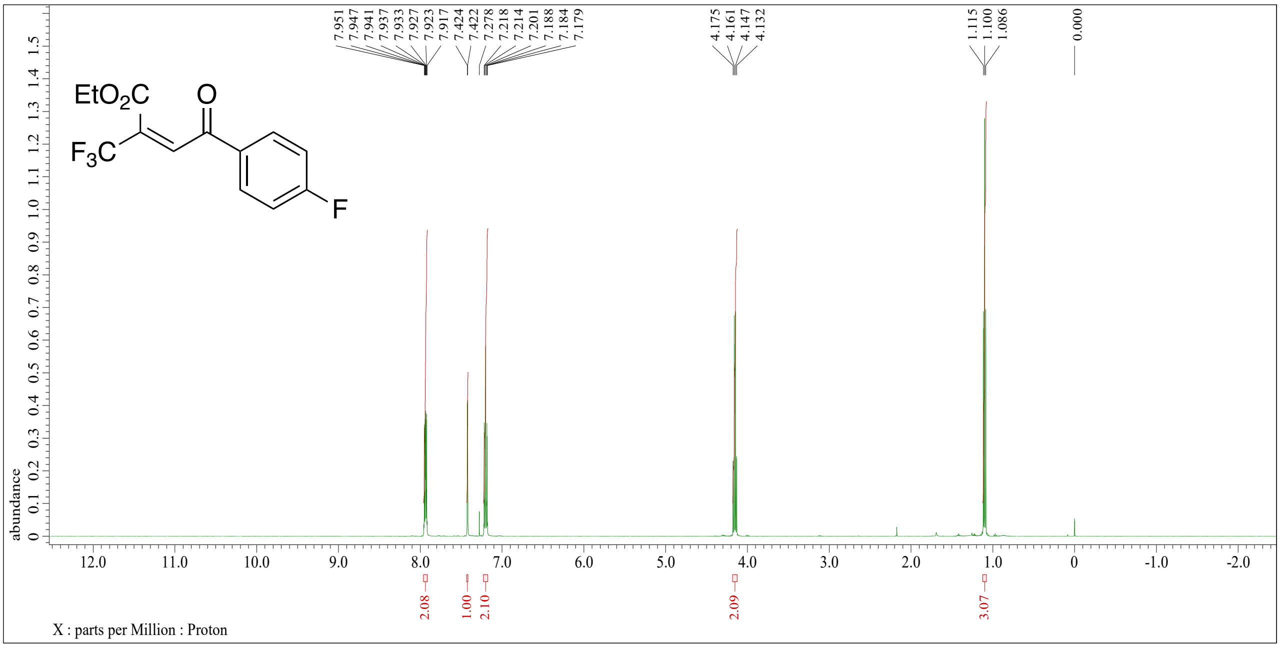

^{13}C NMR (125 MHz, $CDCl_3$) **3d**

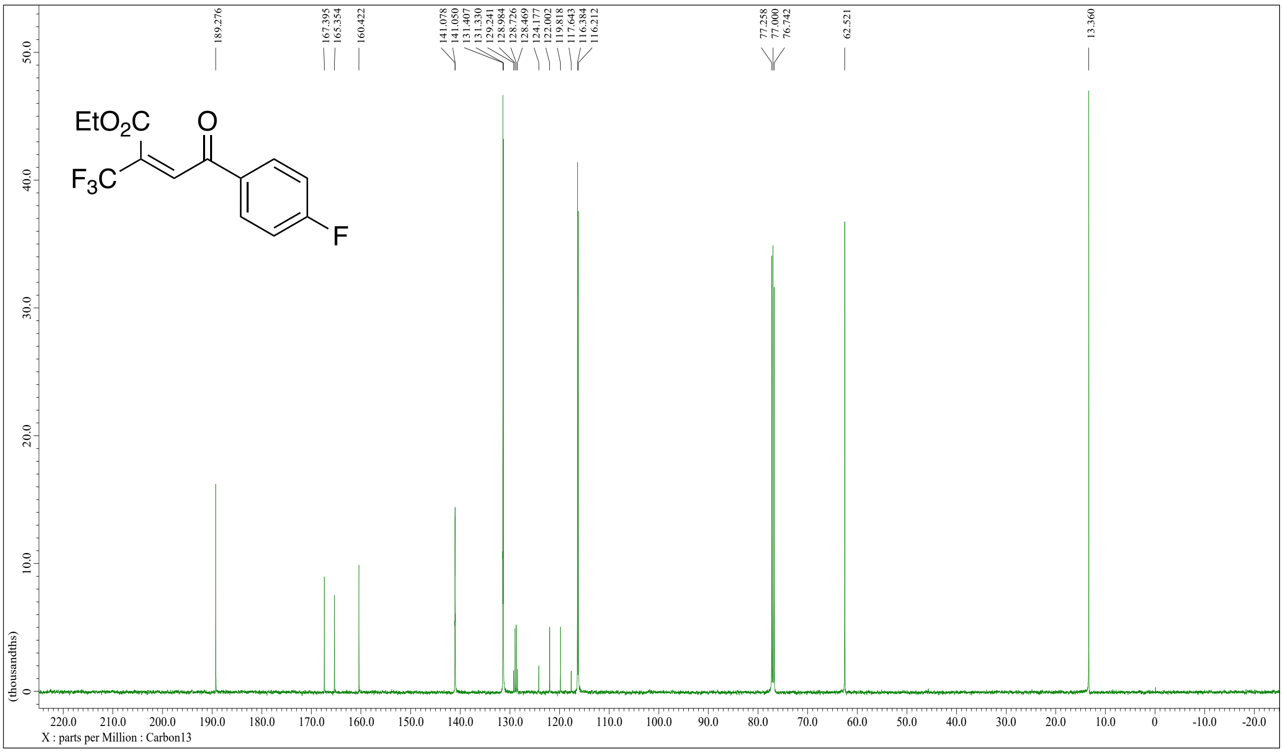

^{19}F NMR (375 MHz, $CDCl_3$) **3d**

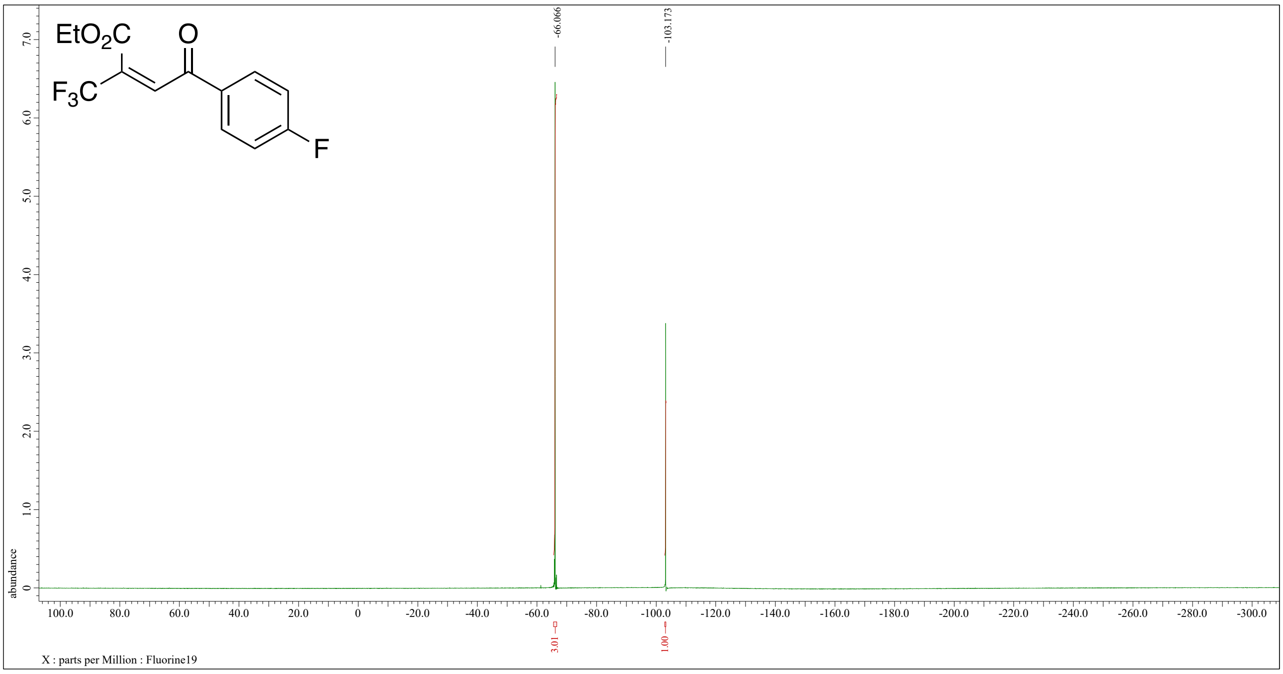

^{1}H NMR (500 MHz, $CDCl_3$) **3e**

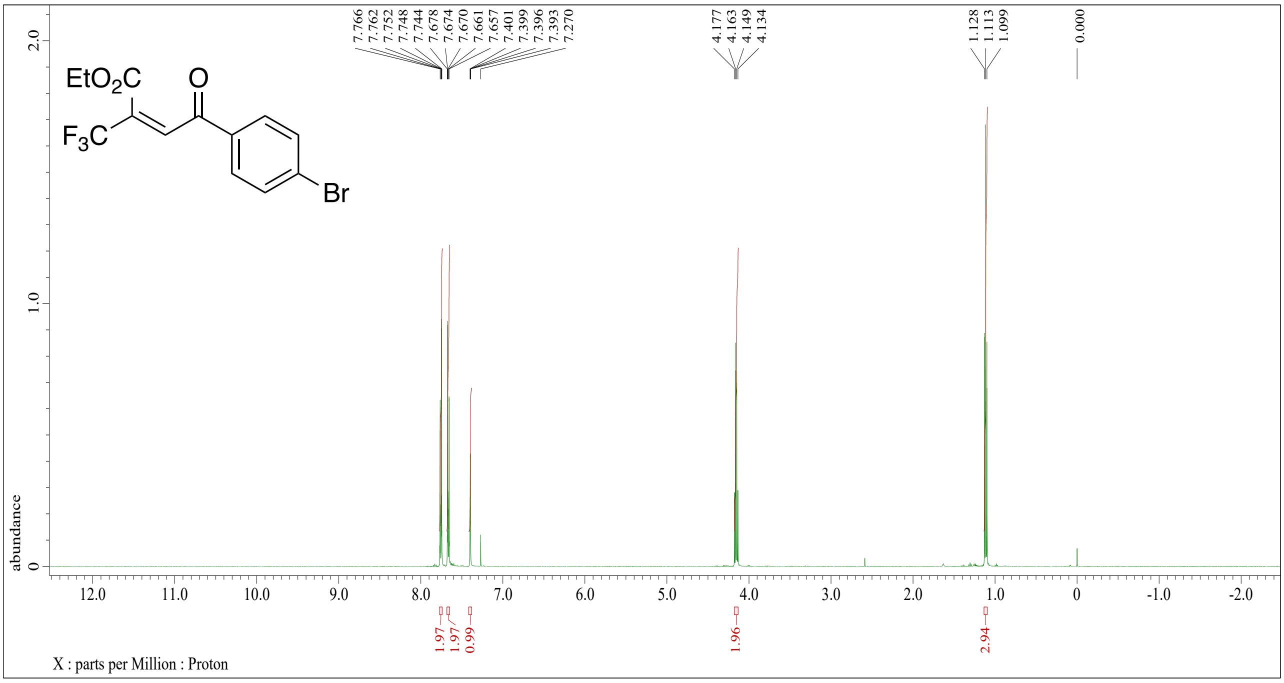

^{13}C NMR (125 MHz, $CDCl_3$) **3e**

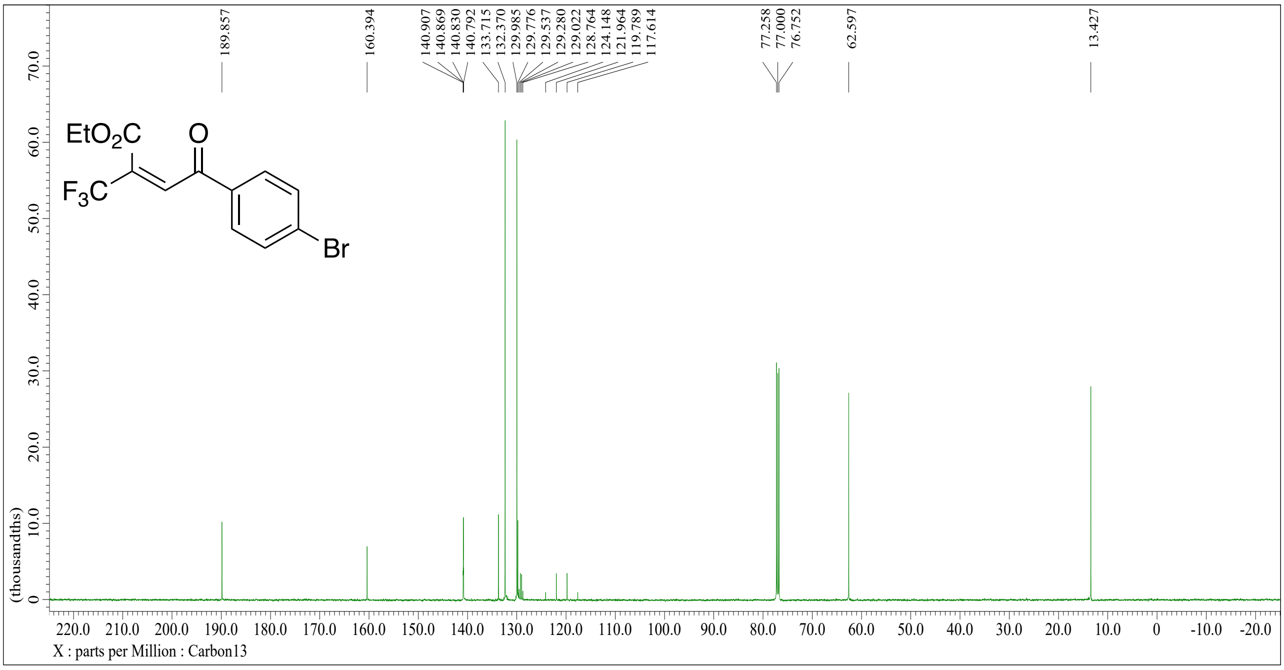

^{19}F NMR (375 MHz, $CDCl_3$) **3e**

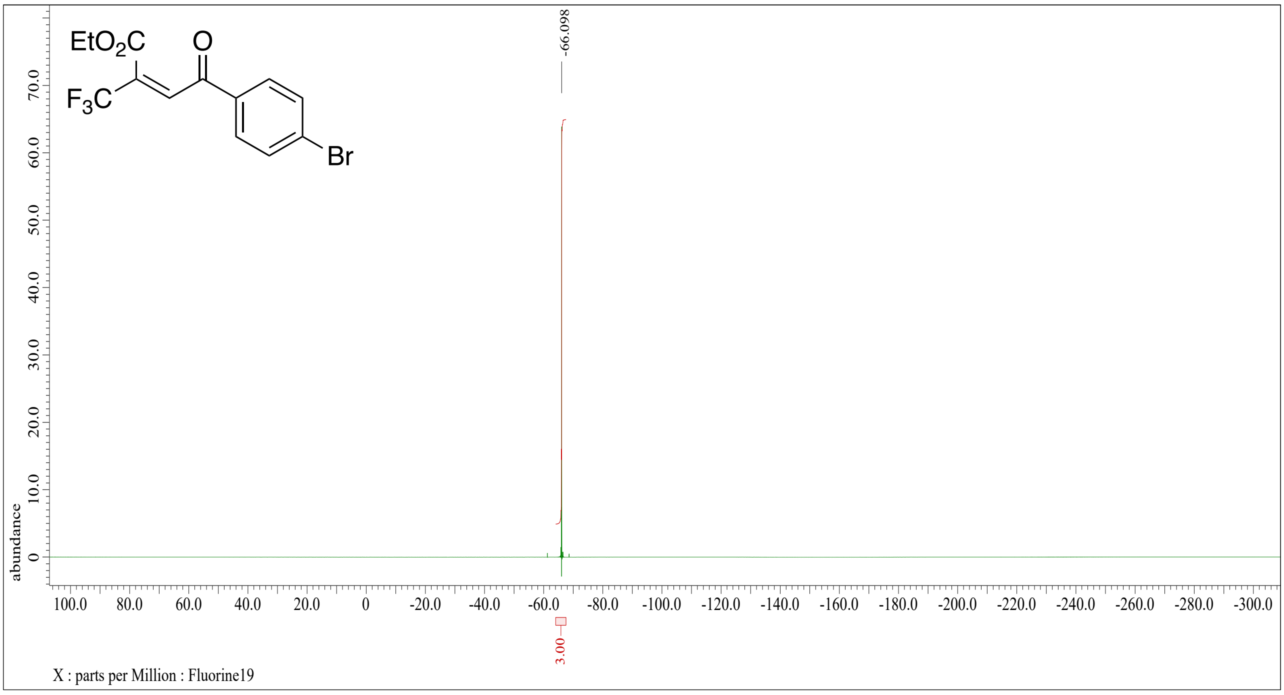

^{1}H NMR (400 MHz, $CDCl_3$) **3f**

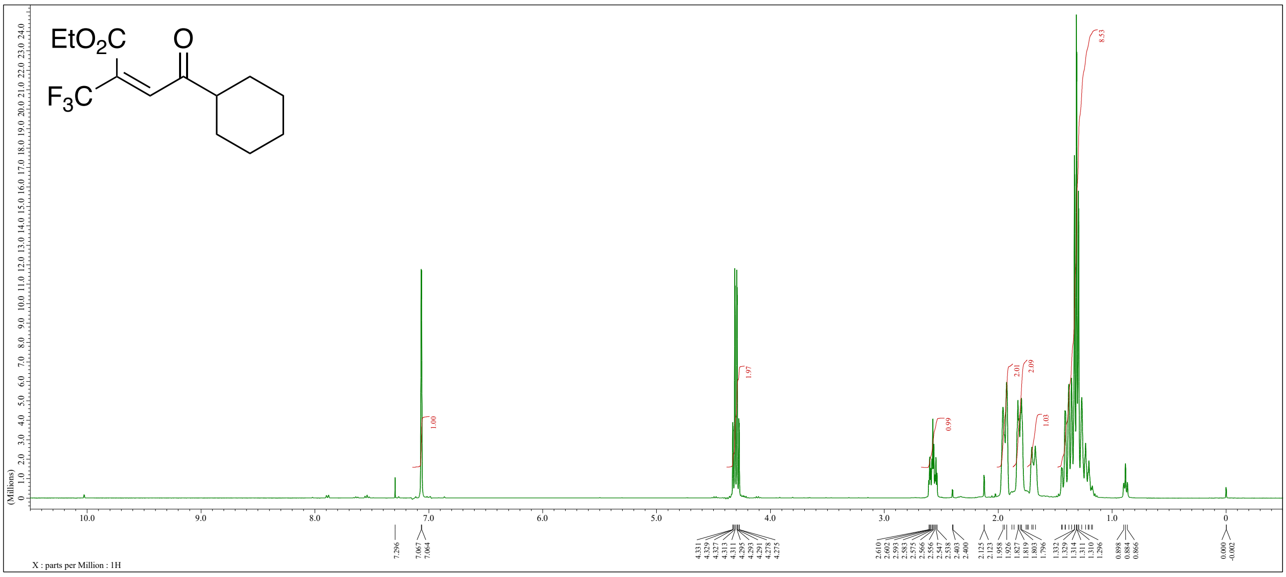

^{13}C NMR (100 MHz, $CDCl_3$) **3f**

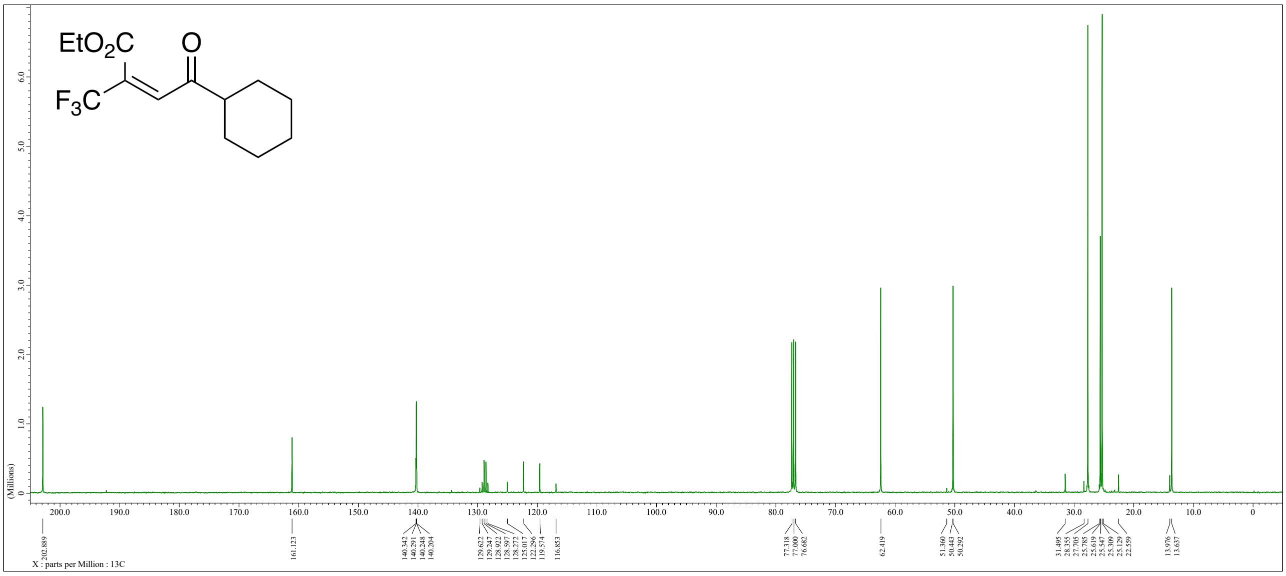

^{19}F NMR (375 MHz, $CDCl_3$) **3f**

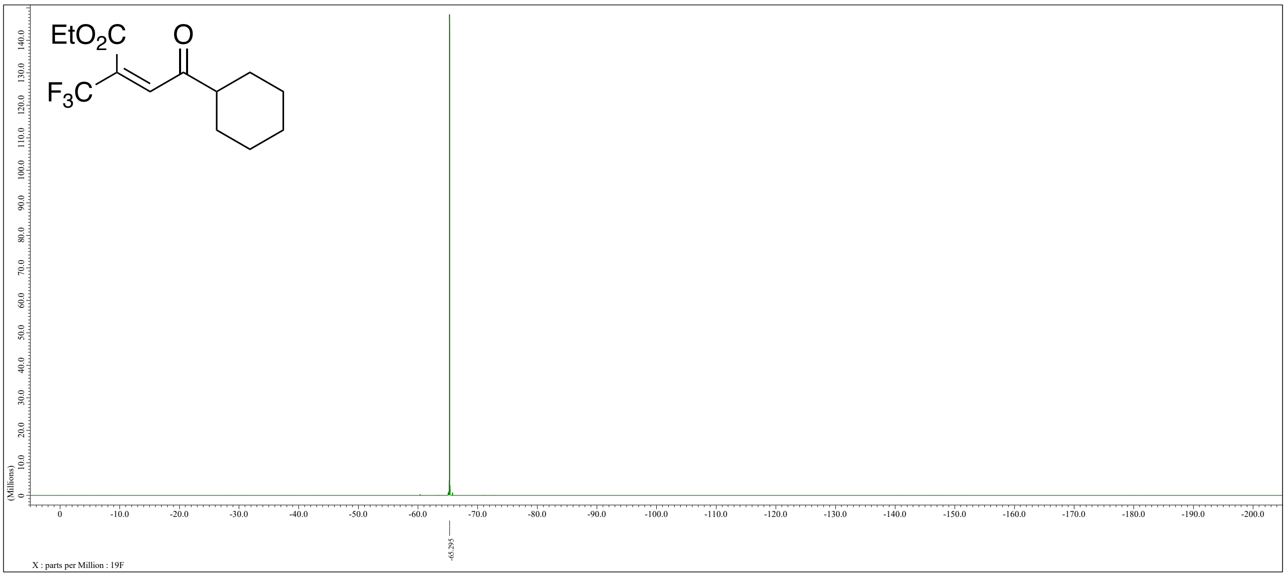

^1H NMR (400 MHz, $CDCl_3$) **3g** (known compound)

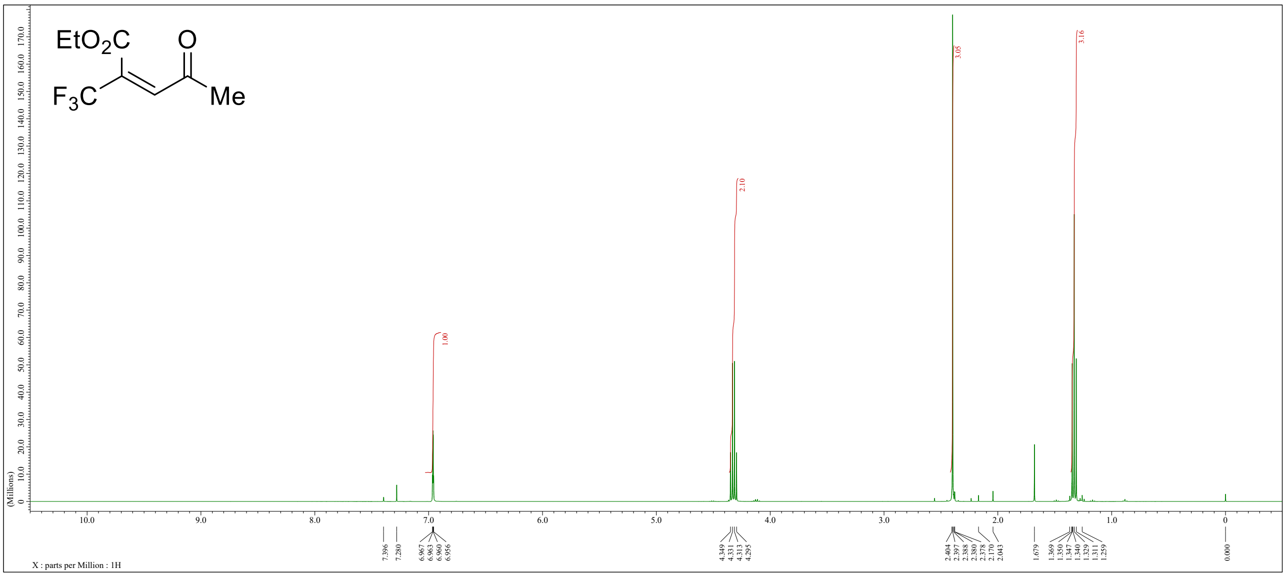

^{1}H NMR (400 MHz, $CDCl_3$) **6**

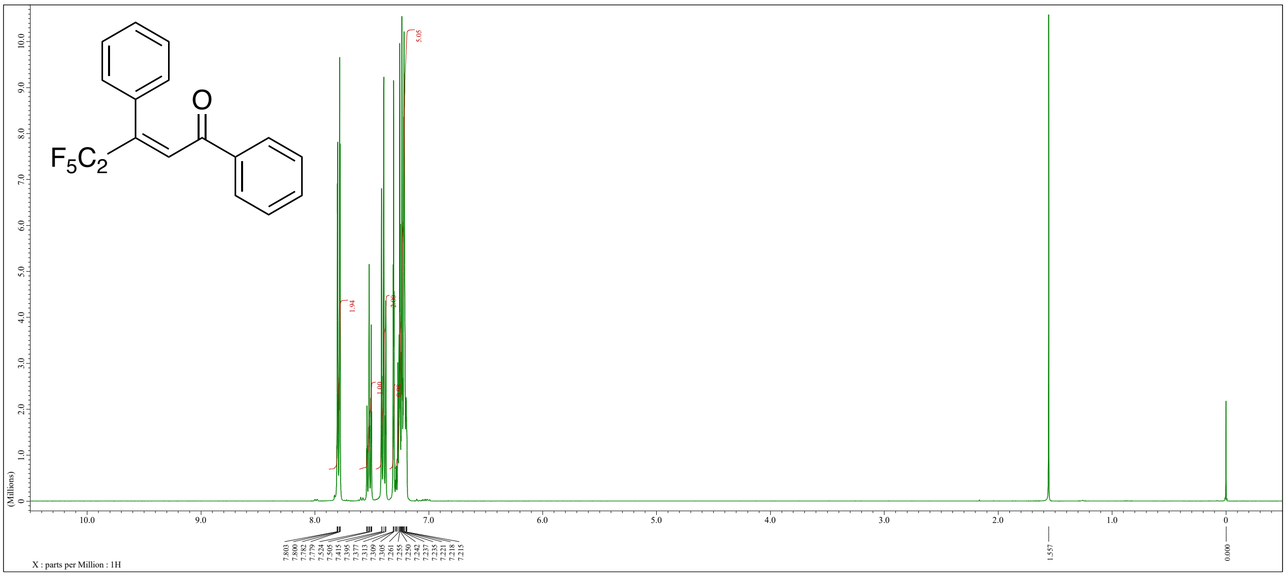

^{13}C NMR (100 MHz, $CDCl_3$) **6**

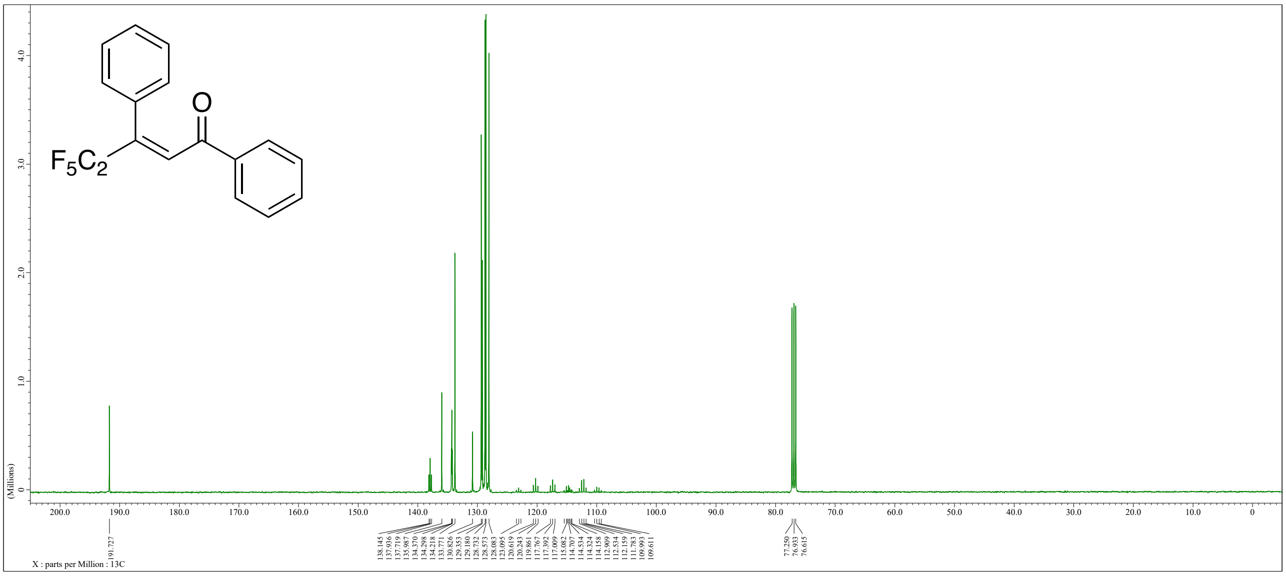

^{19}F NMR (375 MHz, $CDCl_3$) **6**

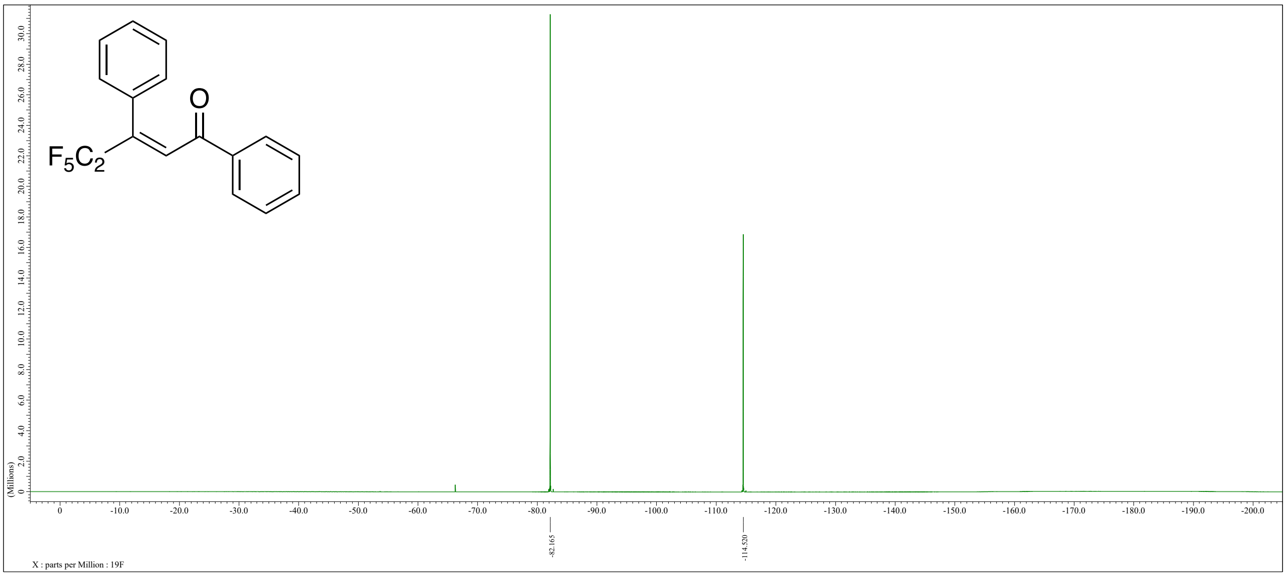

^{1}H NMR (500 MHz, $CDCl_3$) **2a**

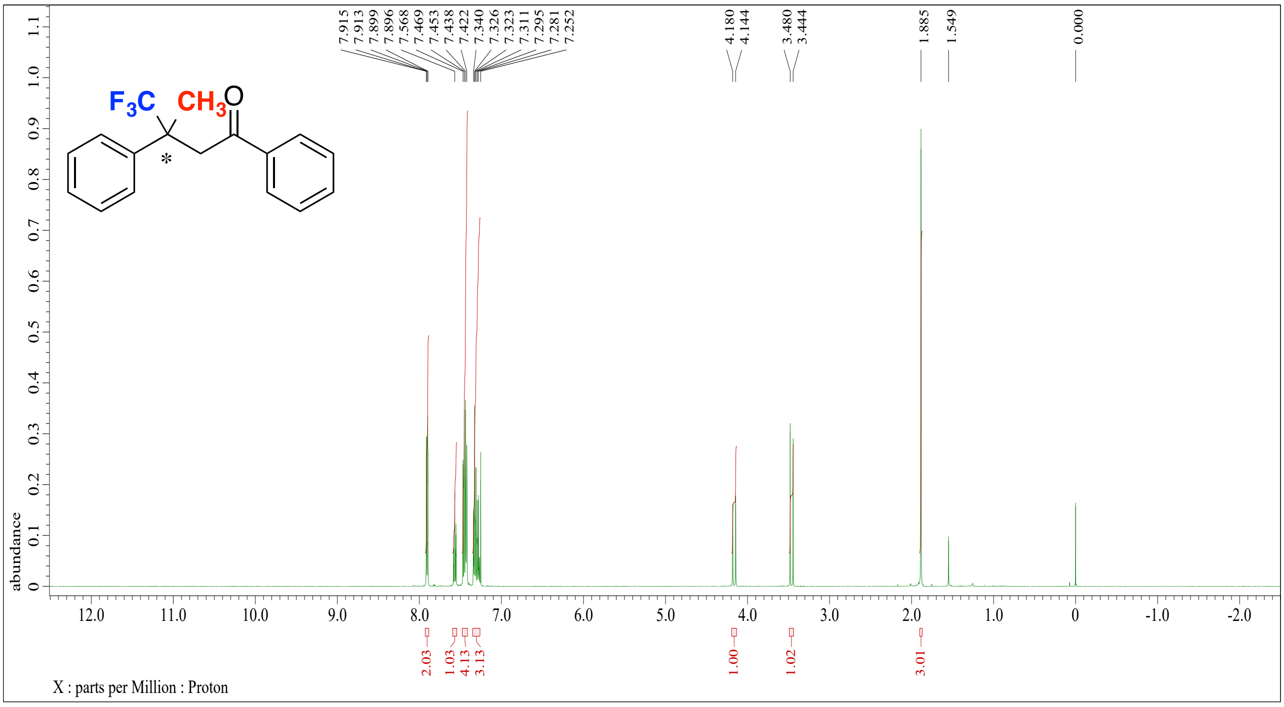

^{13}C NMR (125 MHz, $CDCl_3$) **2a**

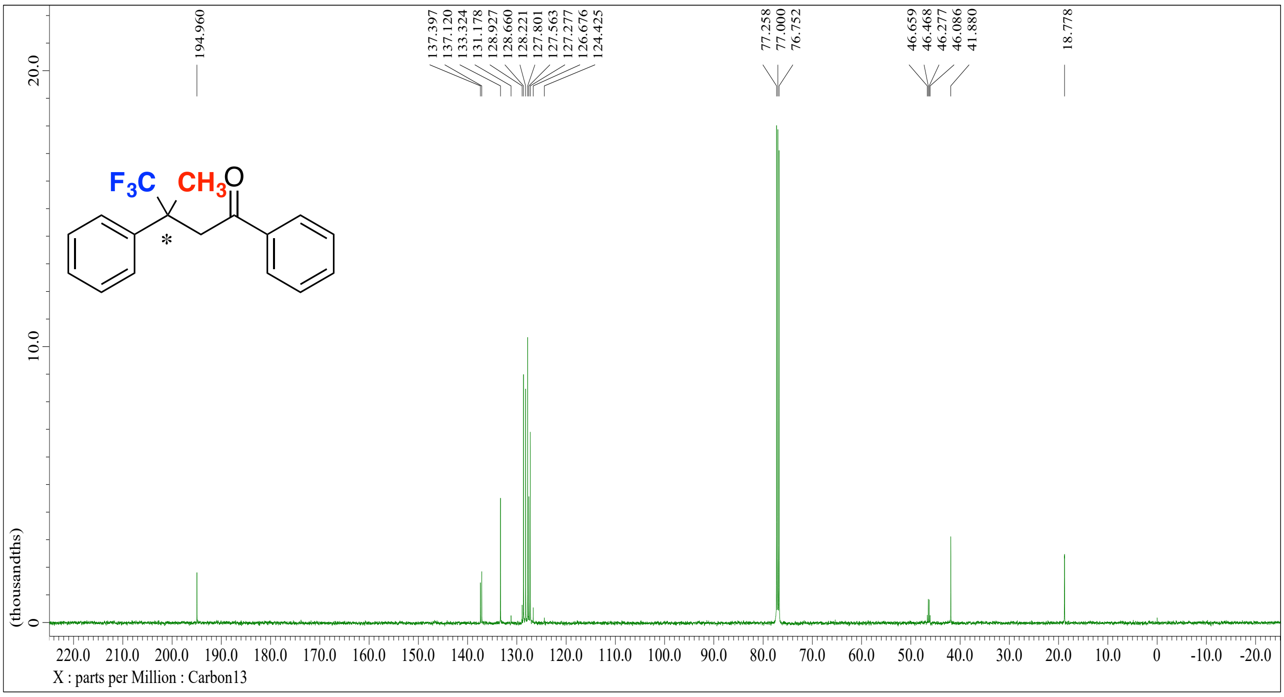

^{19}F NMR (375 MHz, $CDCl_3$) **2a**

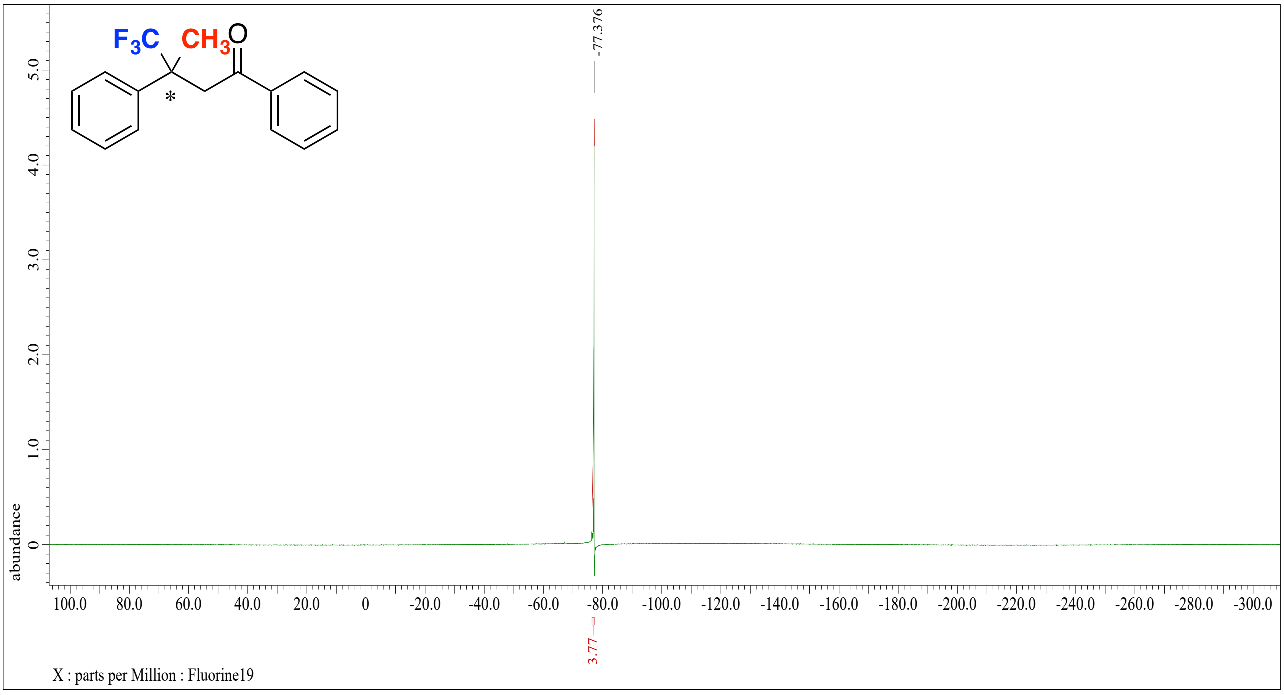

^{1}H NMR (500 MHz, $CDCl_3$) **2b**

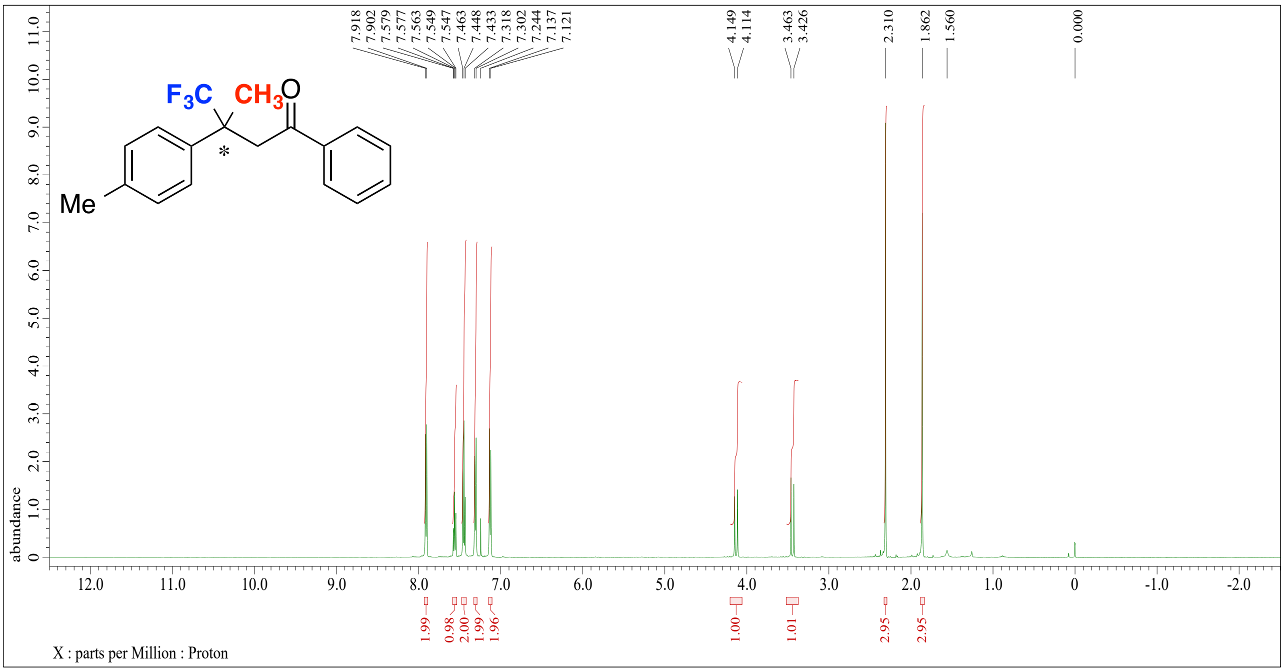

^{13}C NMR (125 MHz, $CDCl_3$) **2b**

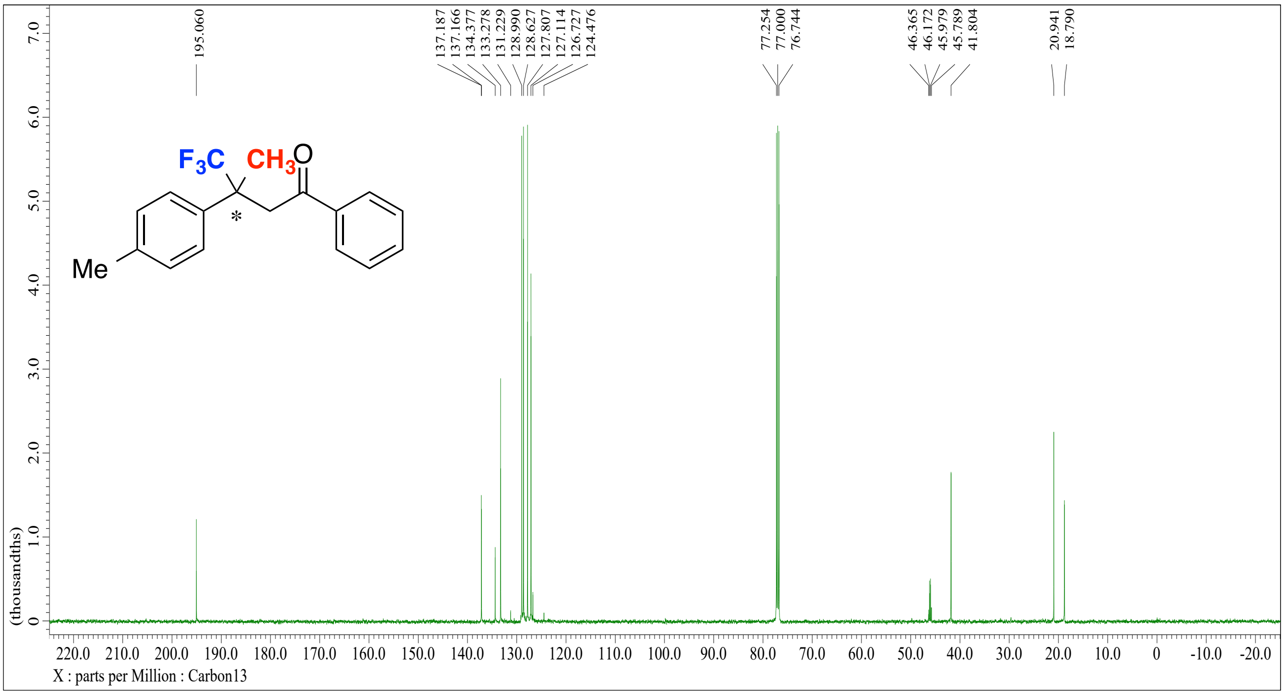

^{19}F NMR (375 MHz, $CDCl_3$) **2b**

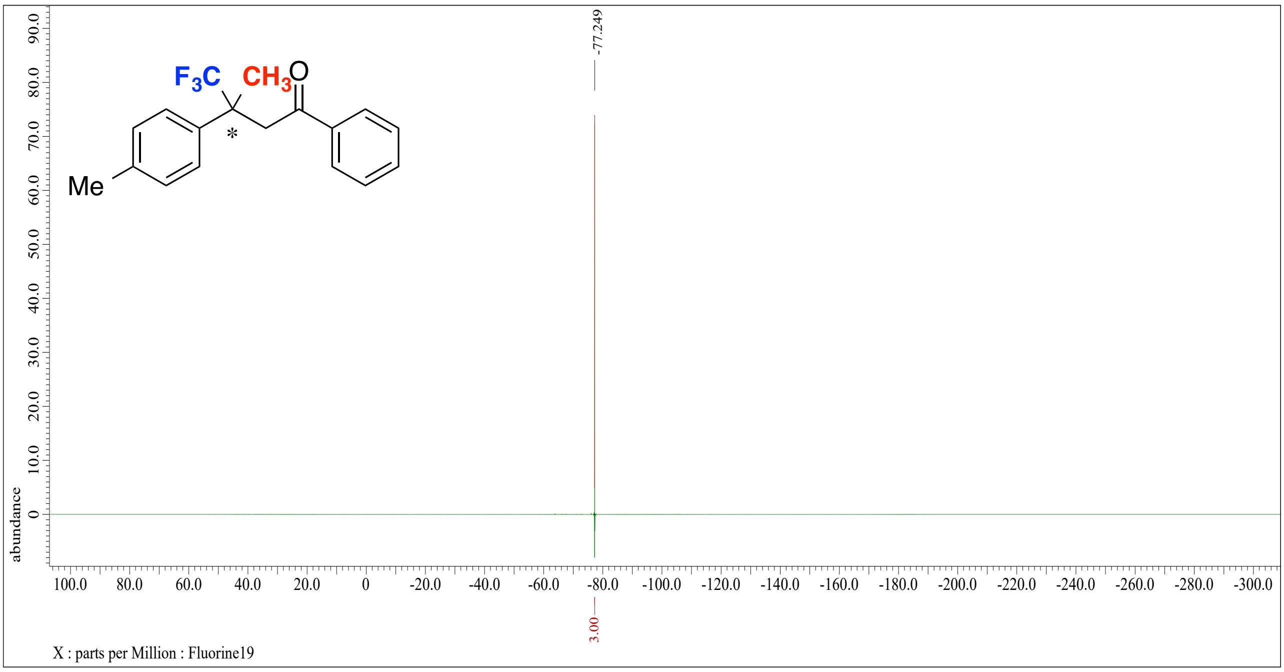

^{1}H NMR (400 MHz, $CDCl_3$) **2c**

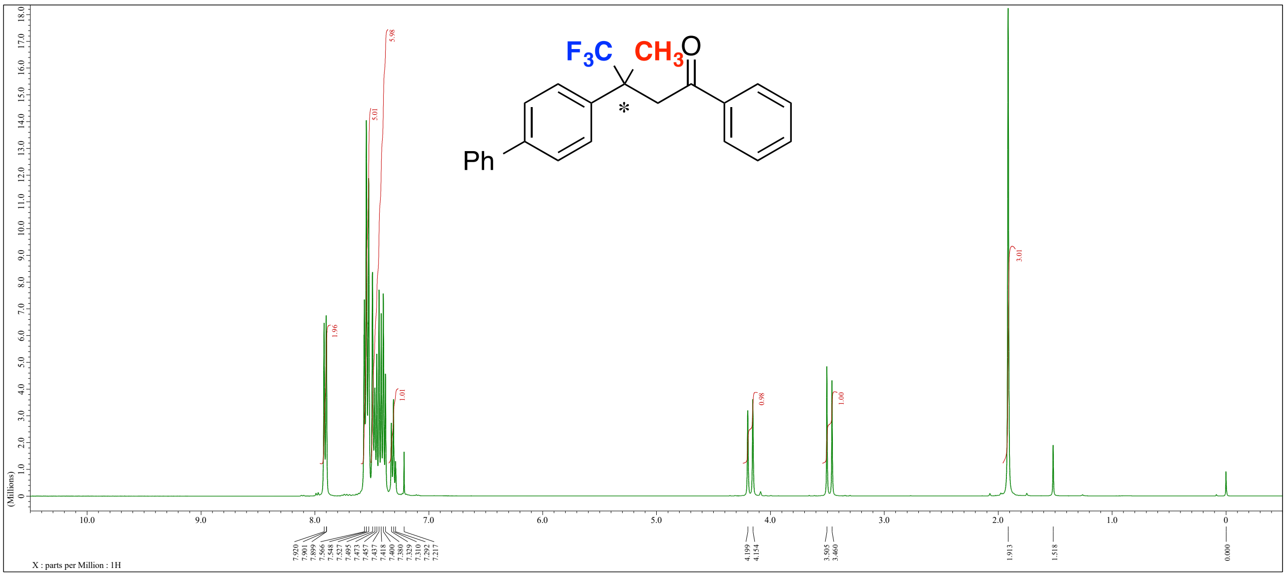

^{13}C NMR (100MHz, $CDCl_3$) **2c**

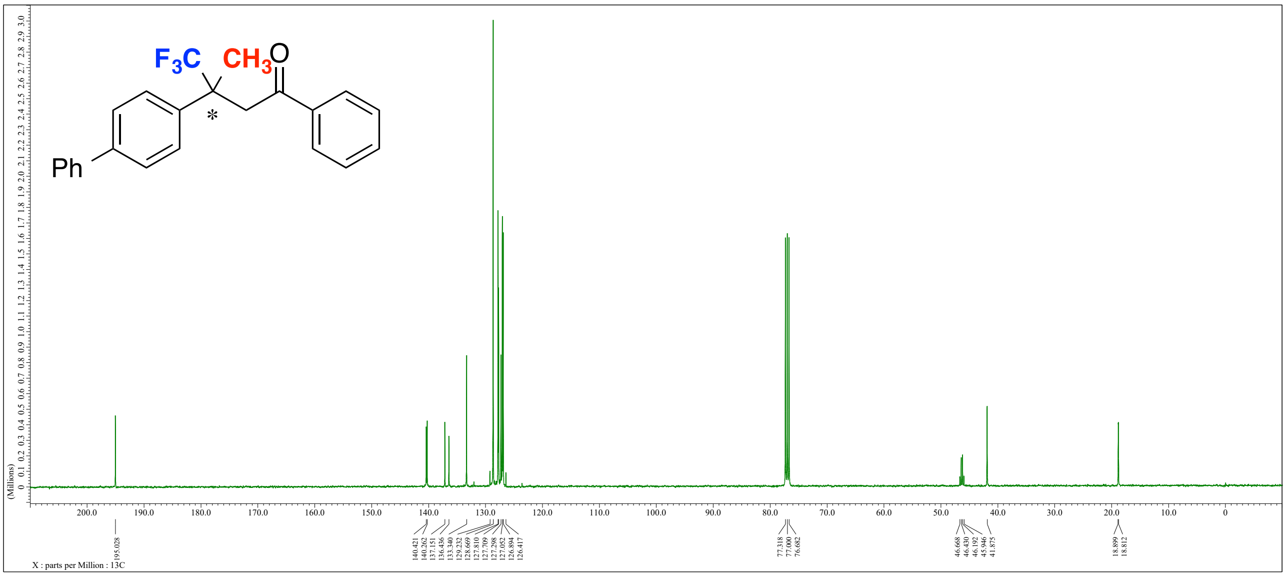

^{19}F NMR (375 MHz, $CDCl_3$) **2c**

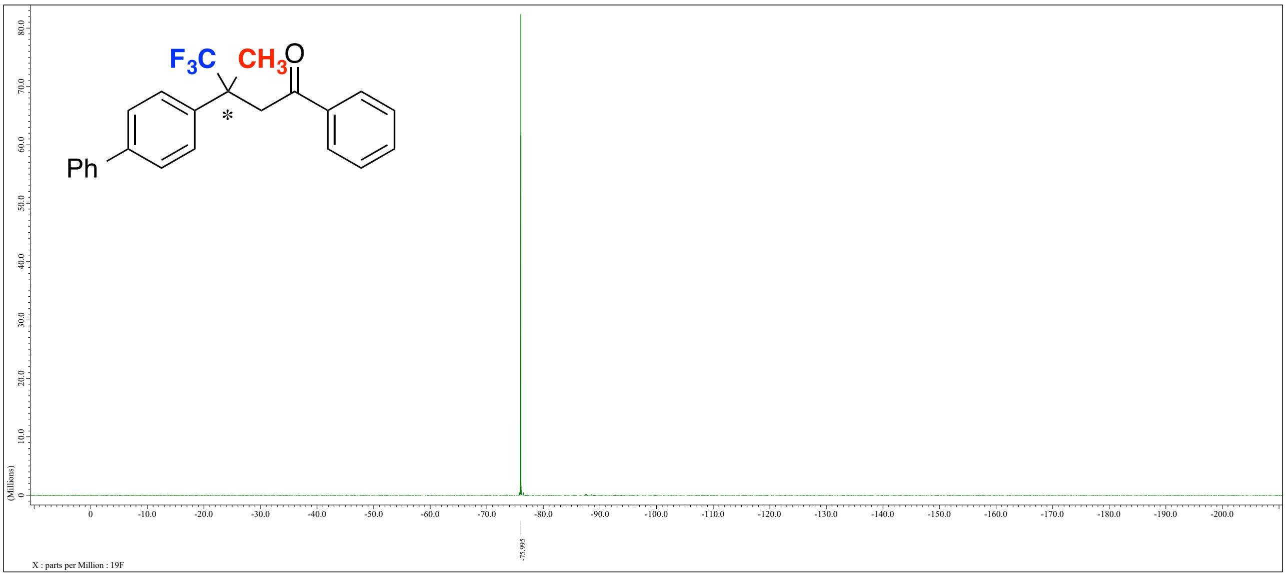

^{1}H NMR (500 MHz, $CDCl_3$) **2d**

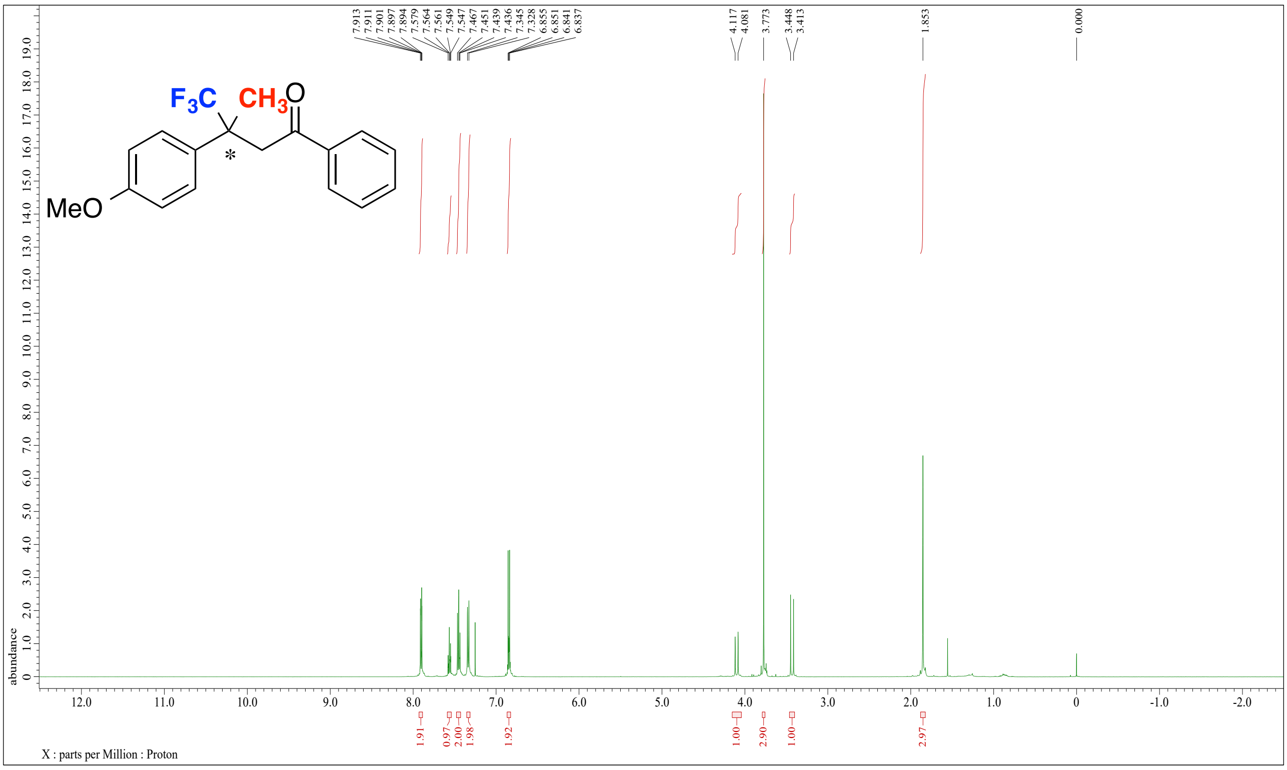

^{13}C NMR (125 MHz, $CDCl_3$) **2d**

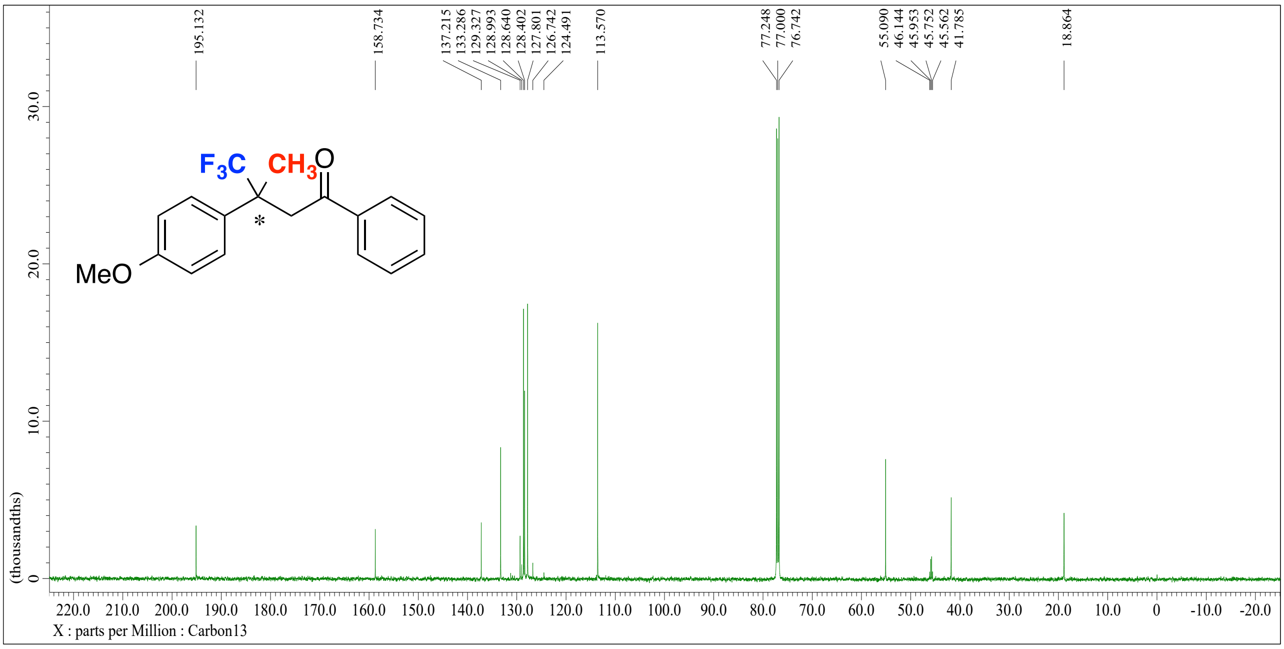

^{19}F NMR (470 MHz, $CDCl_3$) **2d**

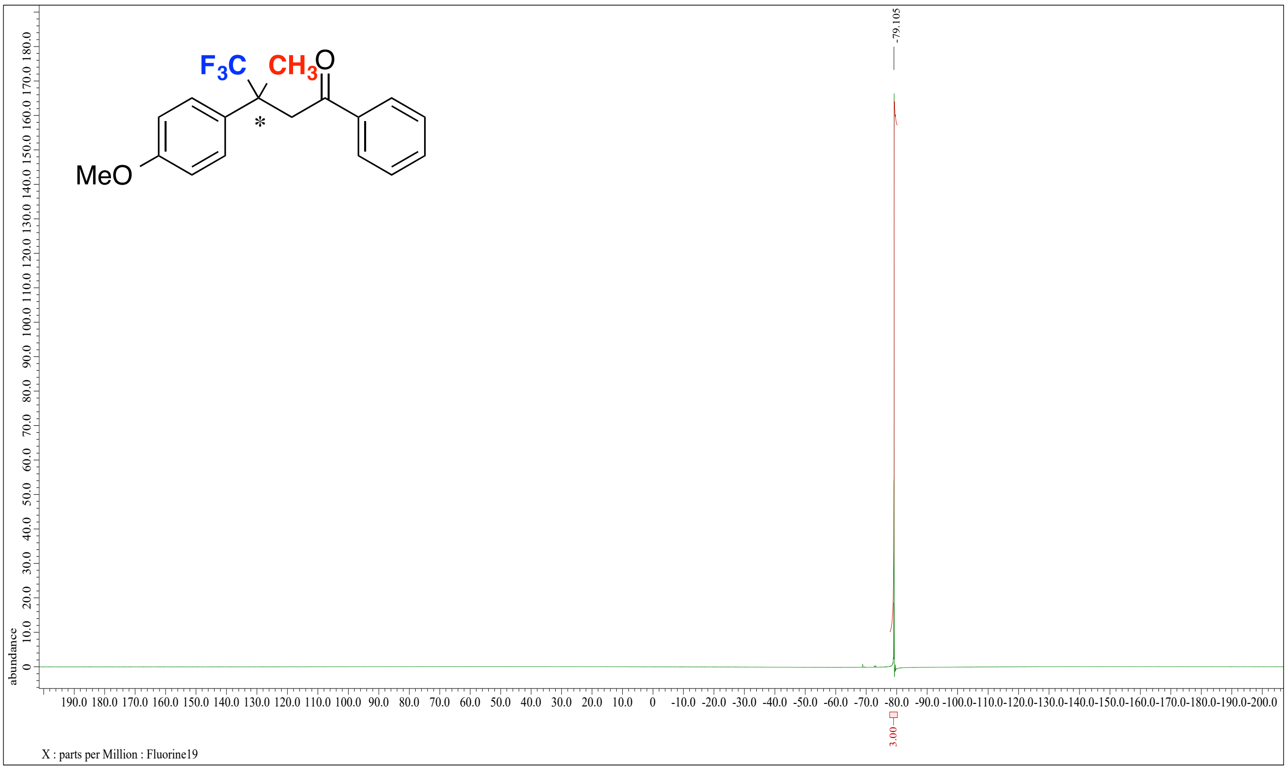

^{1}H NMR (500 MHz, $CDCl_3$) **2e**

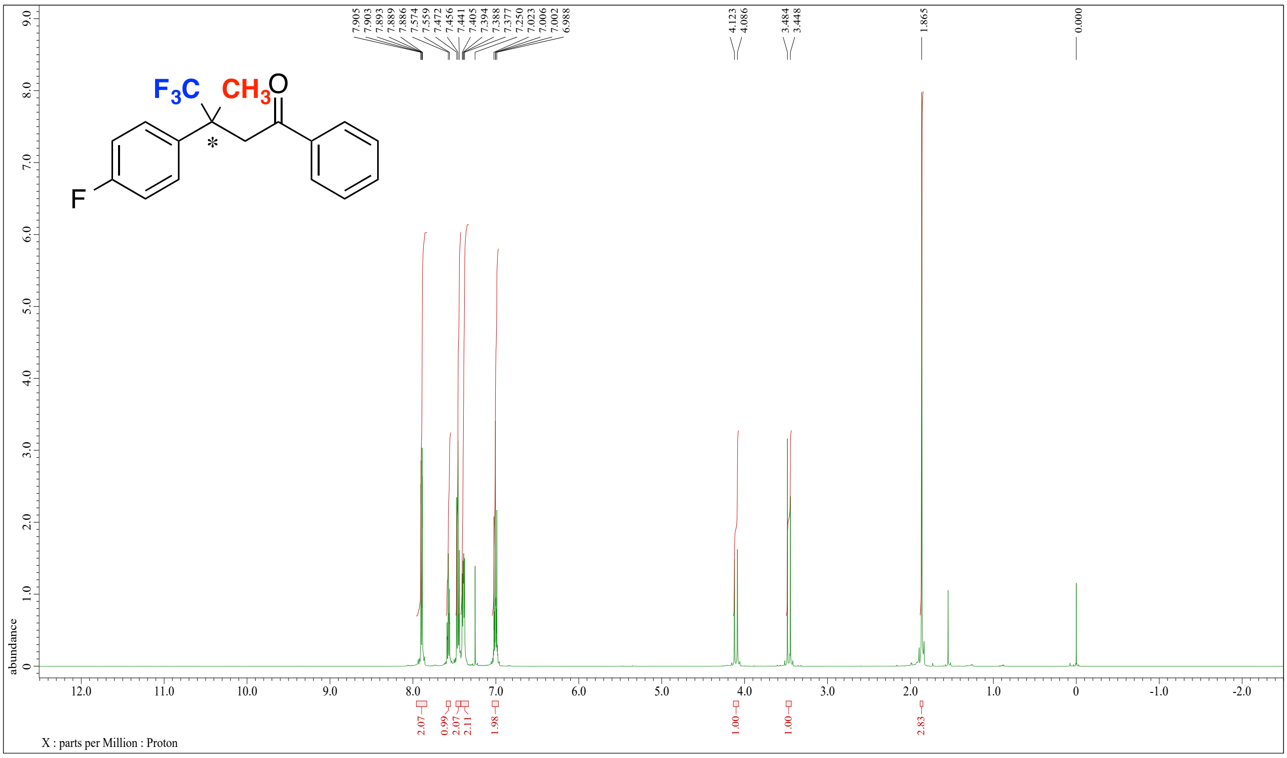

^{13}C NMR (125 MHz, $CDCl_3$) **2e**

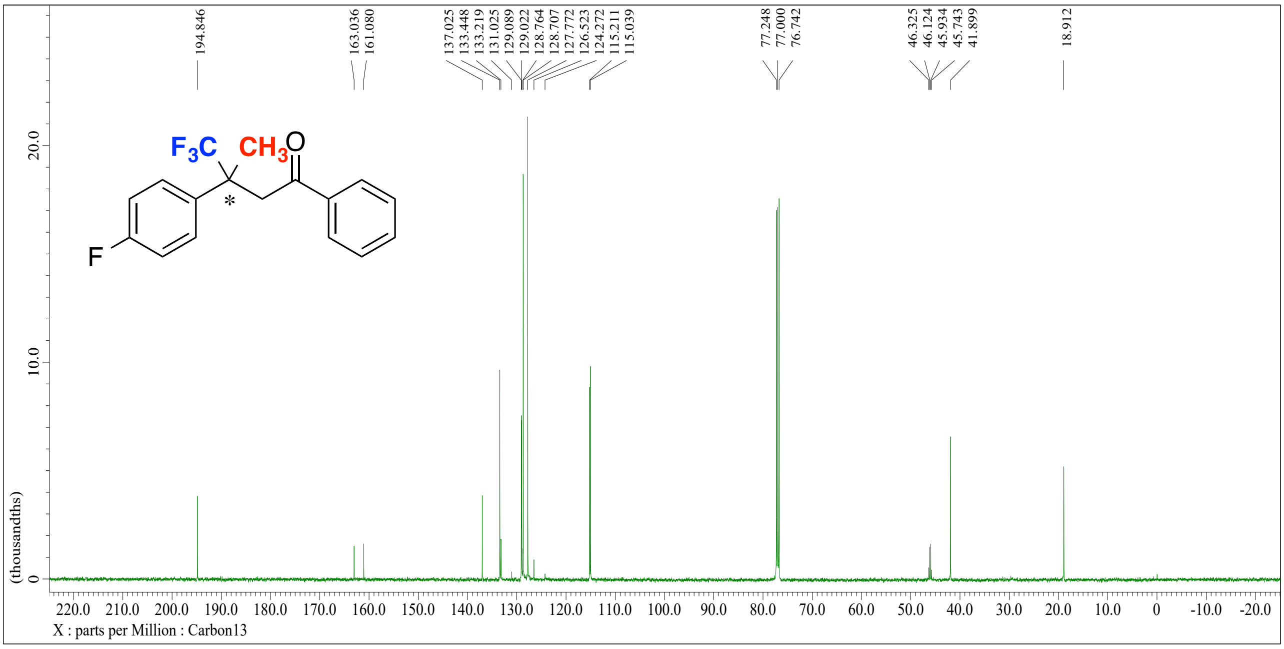

^{19}F NMR (470 MHz, $CDCl_3$) **2e**

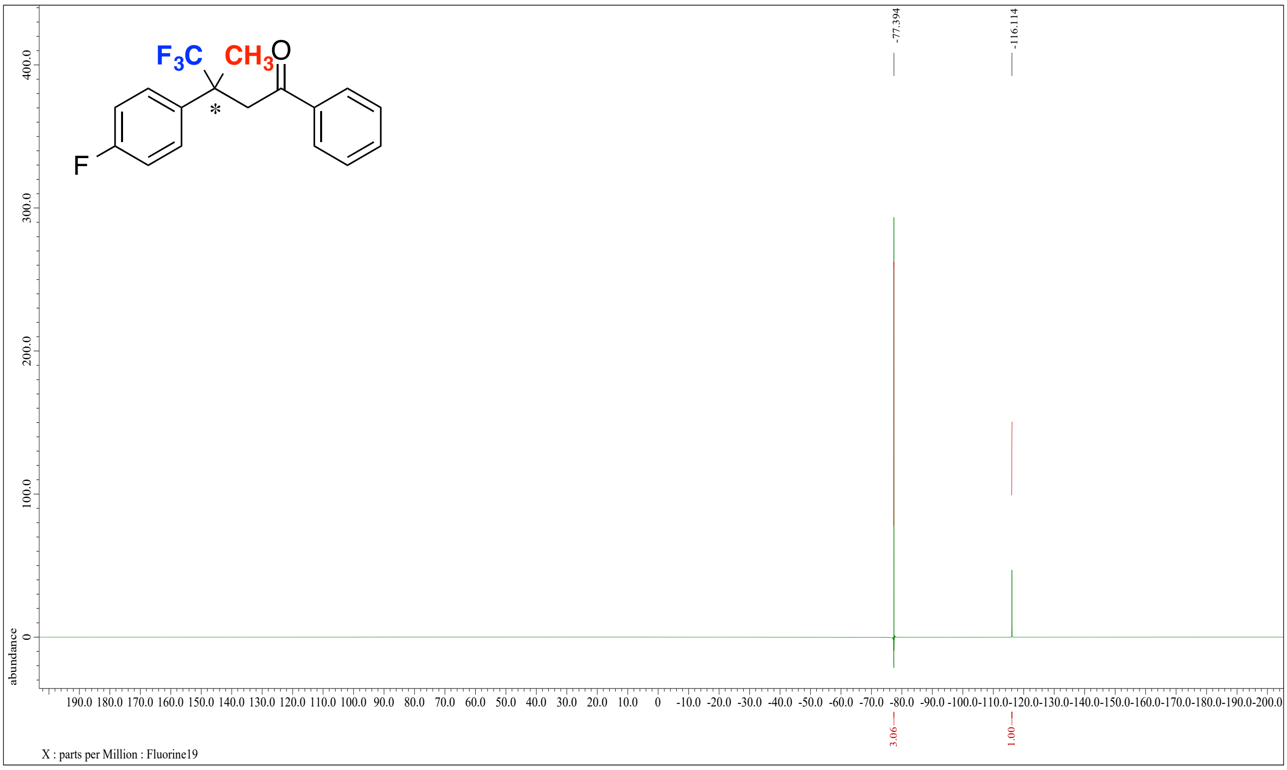

^{1}H NMR (500 MHz, $CDCl_3$) **2g**

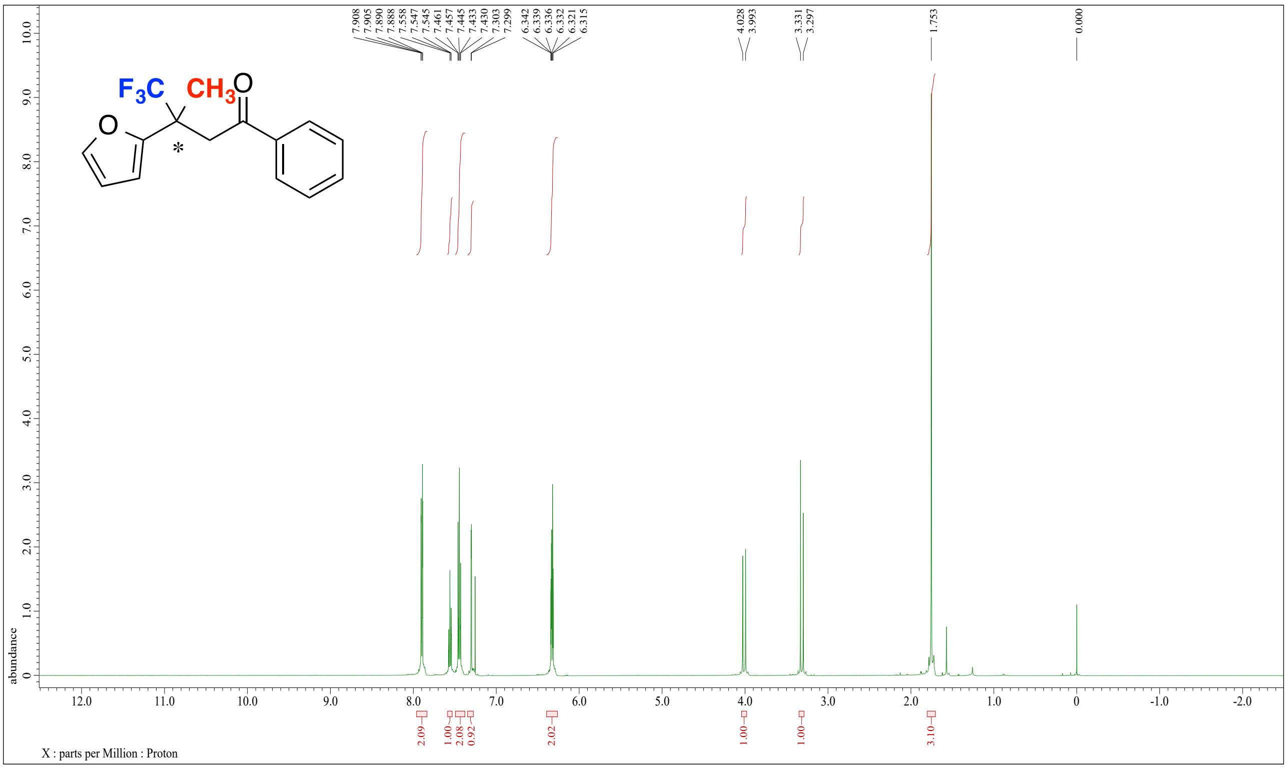

^{13}C NMR (125 MHz, $CDCl_3$) **2g**

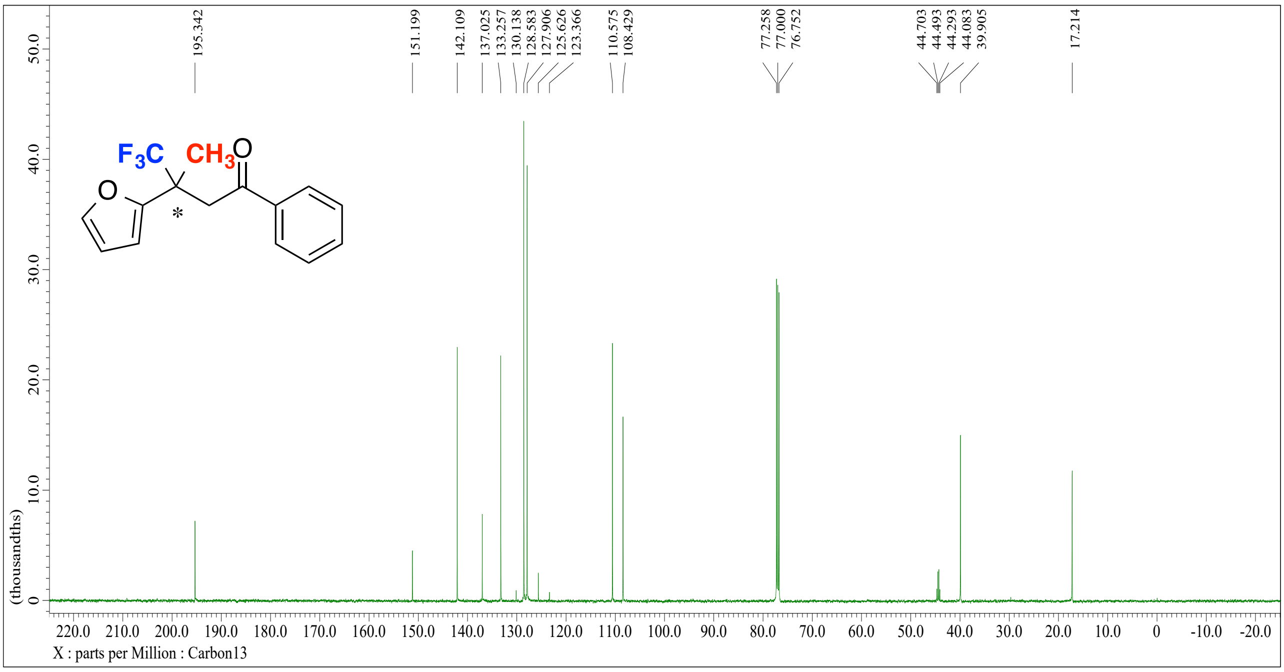

^{19}F NMR (470 MHz, $CDCl_3$) **2g**

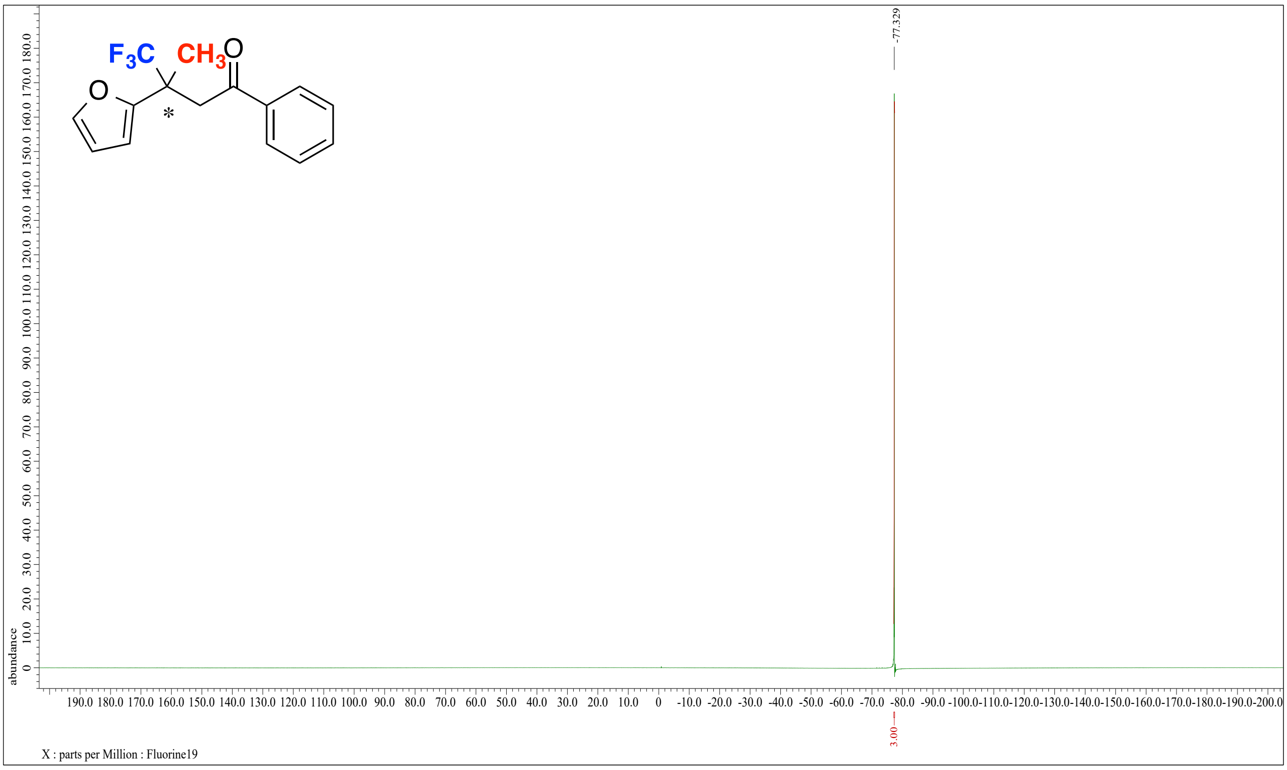

^{1}H NMR (500 MHz, $CDCl_3$) **2h**

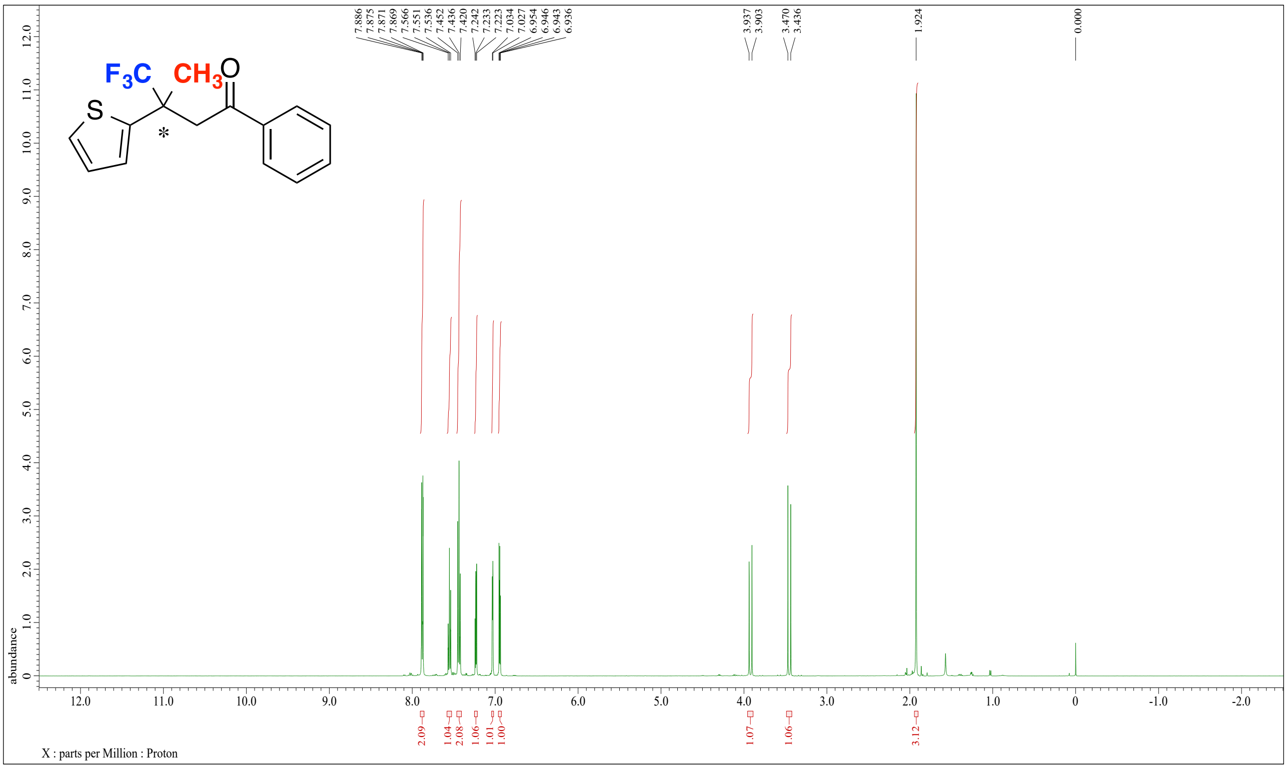

^{13}C NMR (125 MHz, $CDCl_3$) **2h**

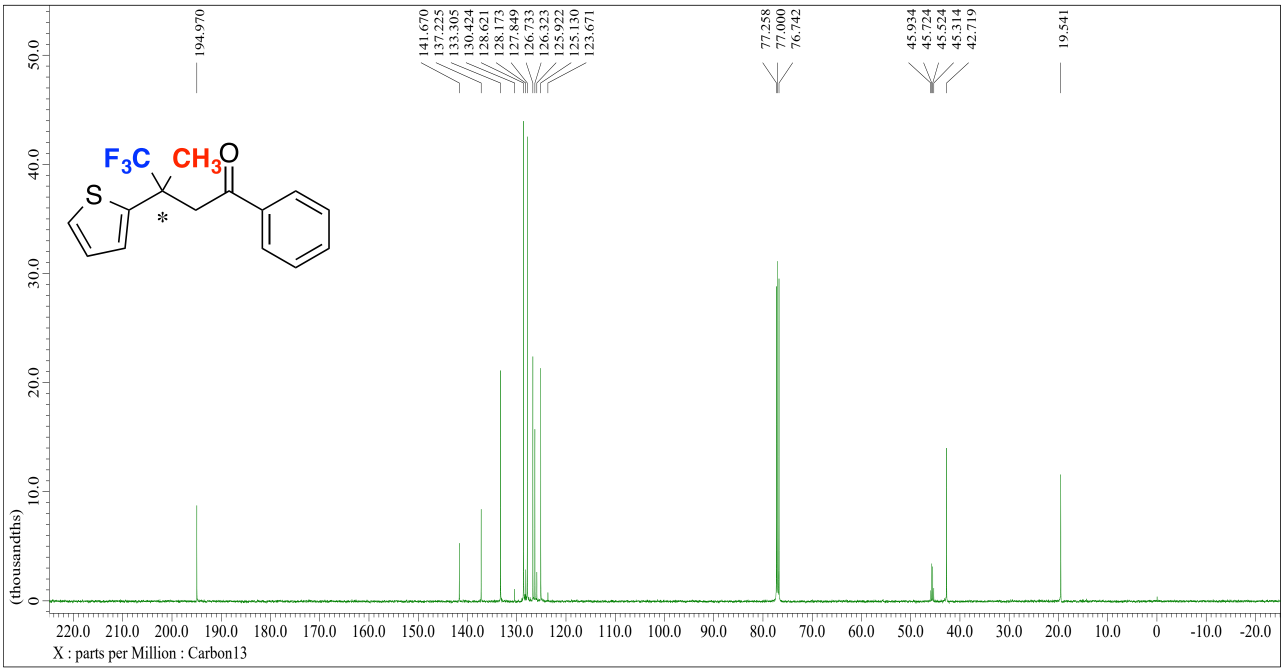

^{19}F NMR (375 MHz, $CDCl_3$) **2h**

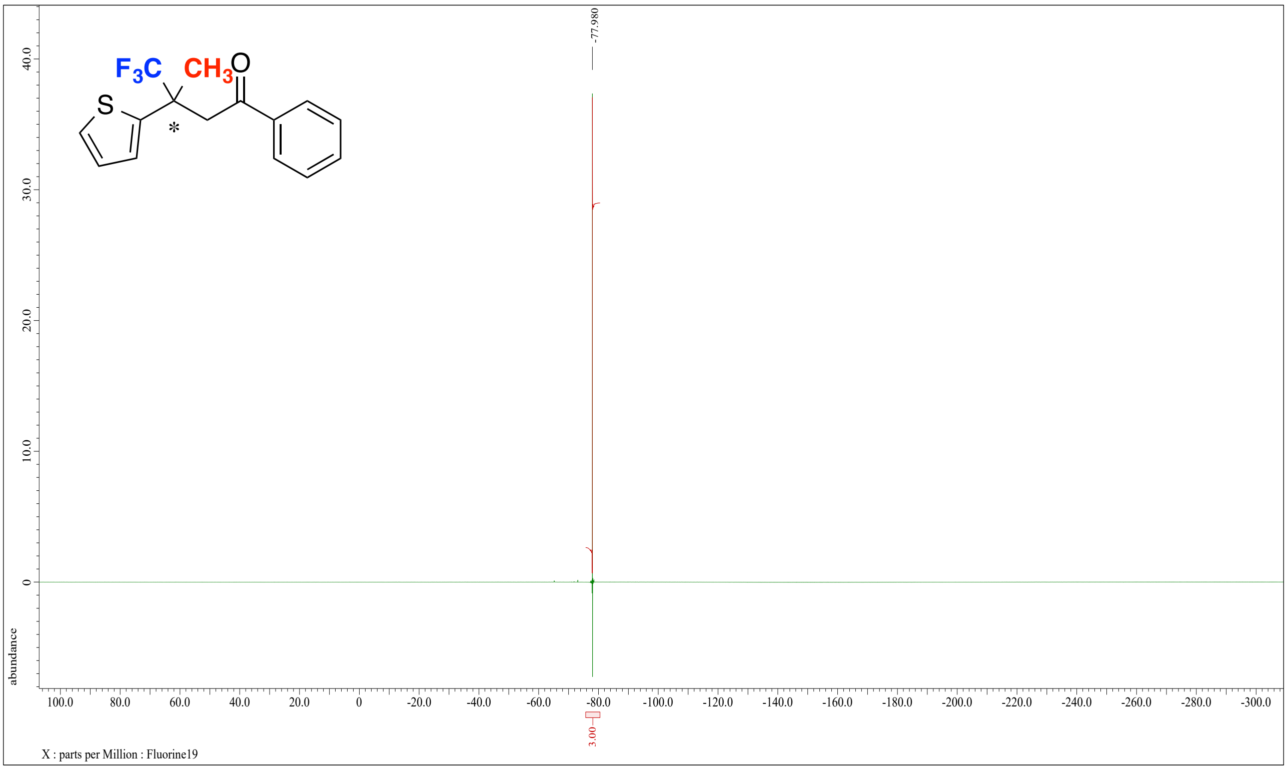

^{1}H NMR (400 MHz, $CDCl_3$) **2i**

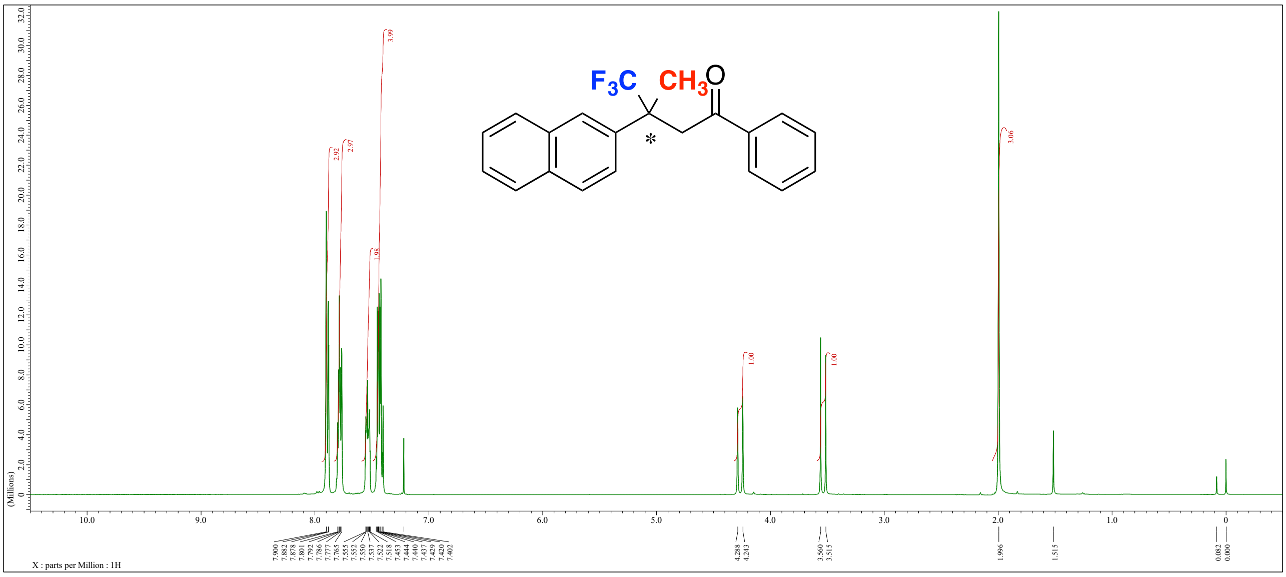

^{13}C NMR (100MHz, $CDCl_3$) **2i**

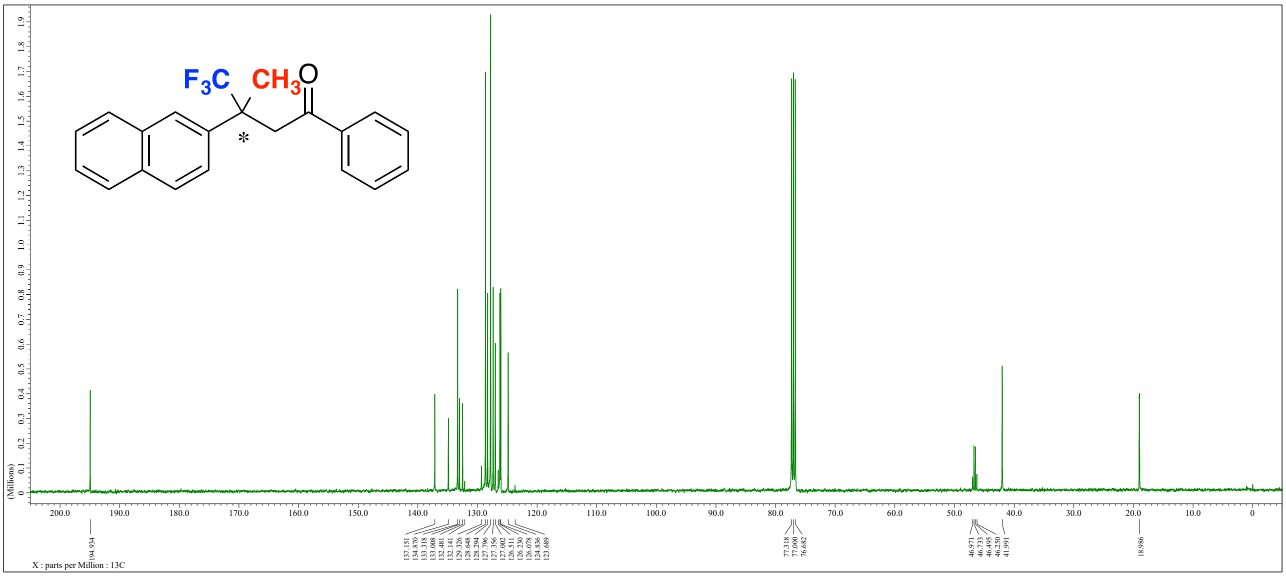

^{19}F NMR (375 MHz, $CDCl_3$) **2i**

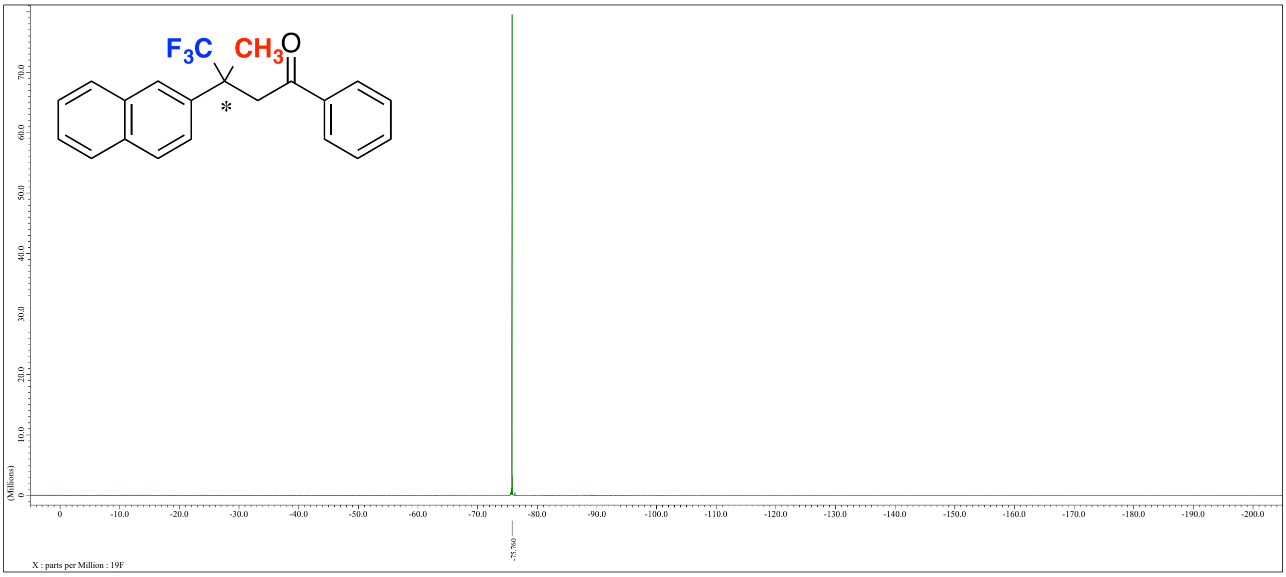

^{1}H NMR (500 MHz, $CDCl_3$) **4a**

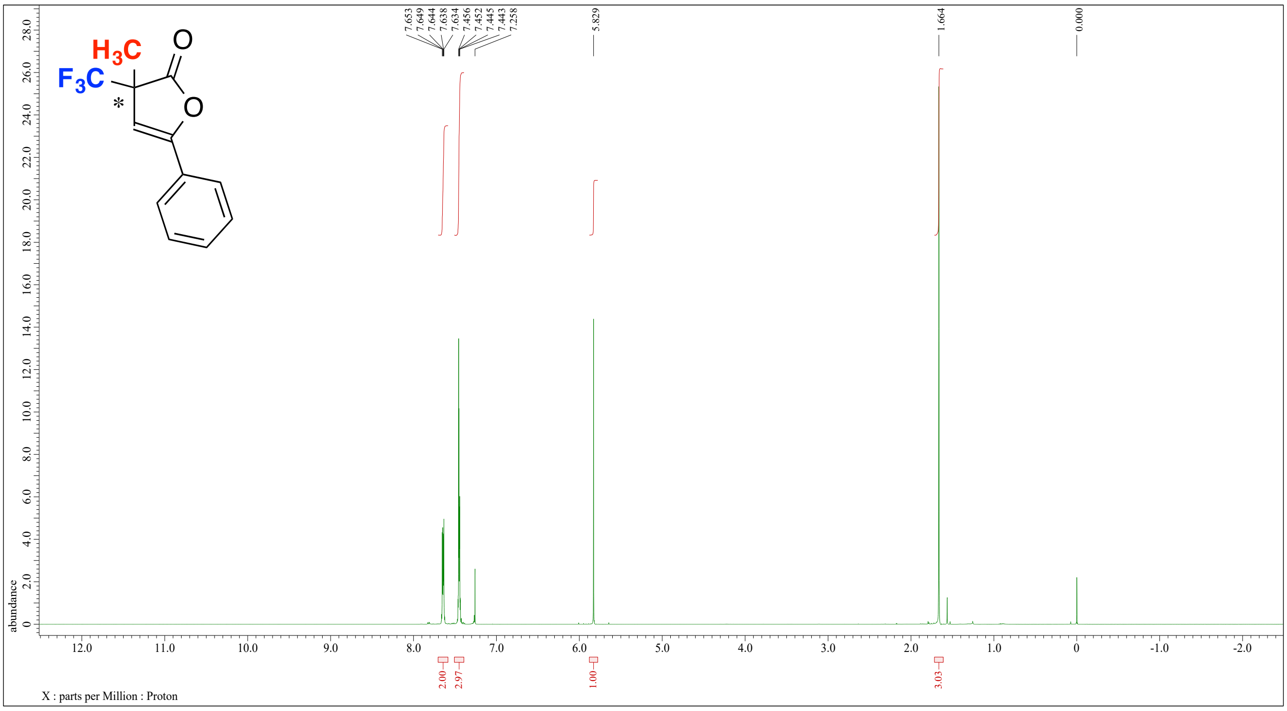

^{13}C NMR (125 MHz, $CDCl_3$) **4a**

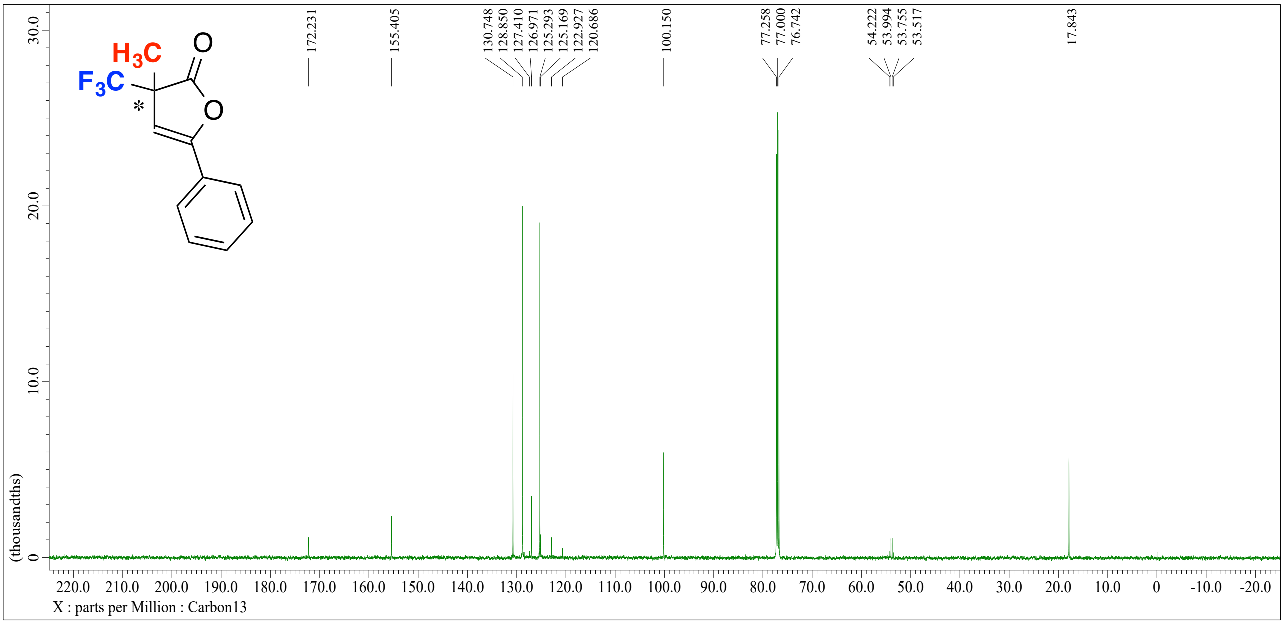

^{19}F NMR (375 MHz, $CDCl_3$) **4a**

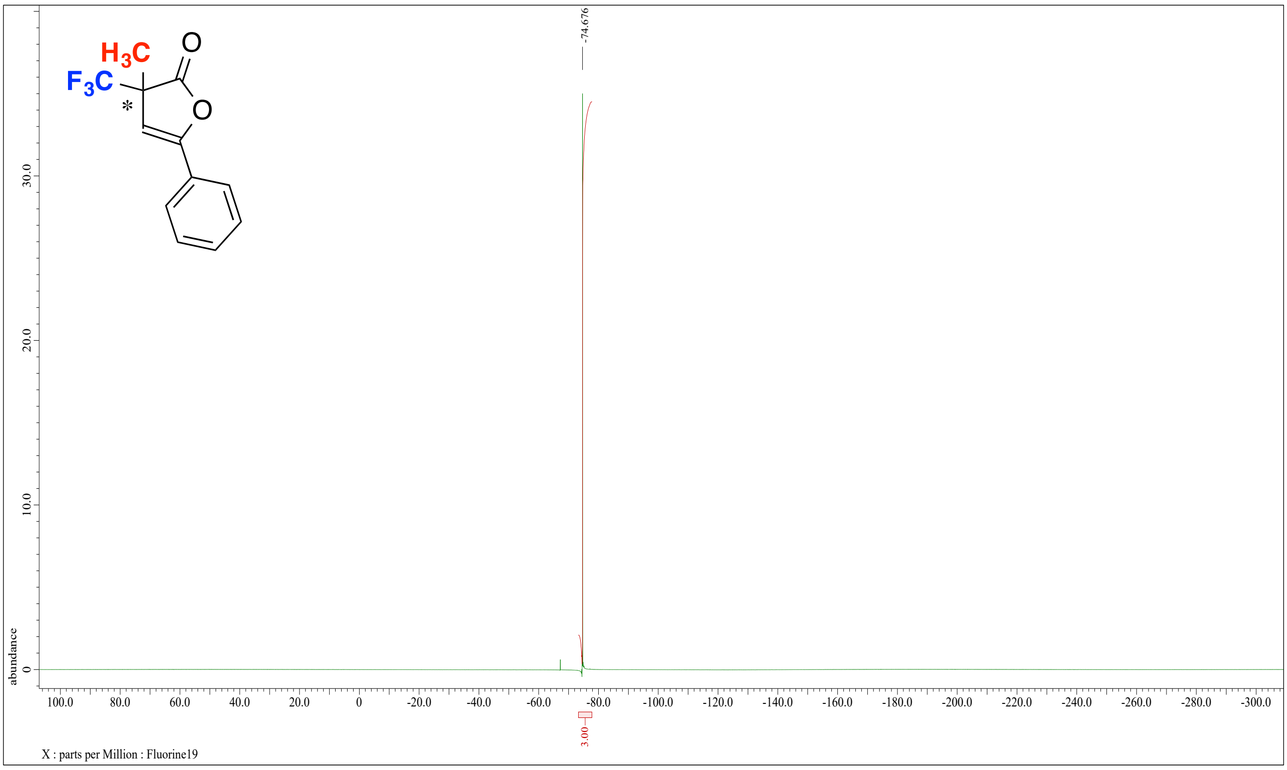

^{1}H NMR (500 MHz, $CDCl_3$) **4b**

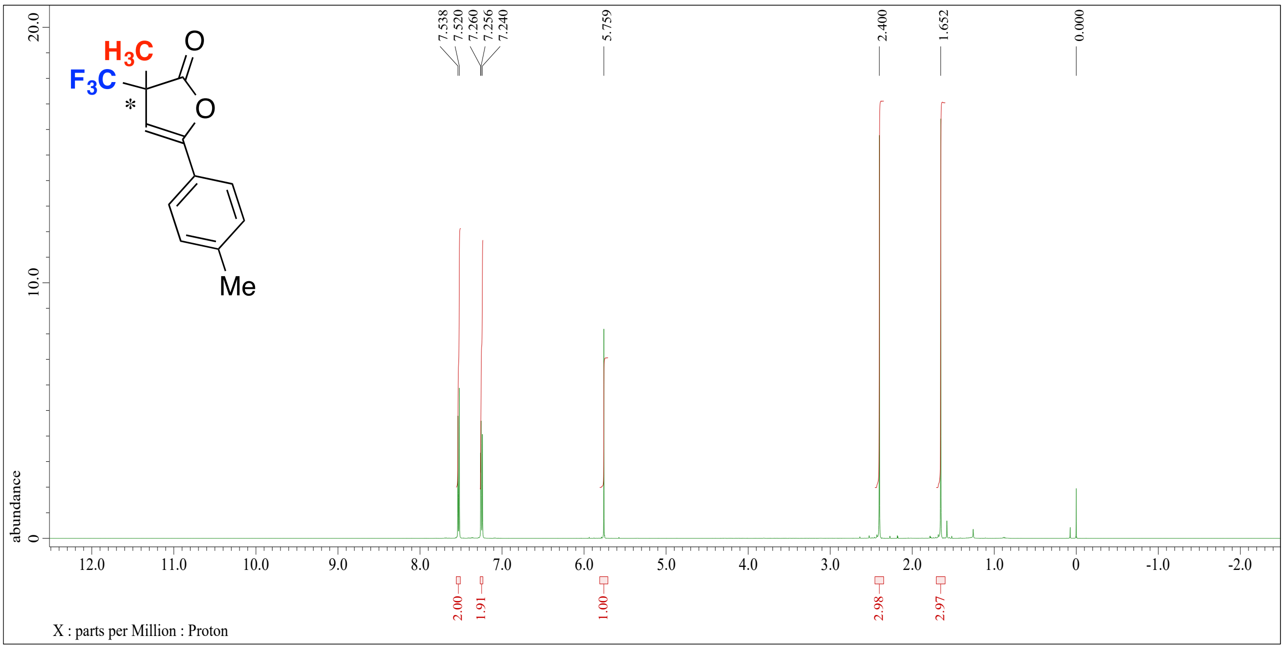

^{13}C NMR (125 MHz, $CDCl_3$) **4b**

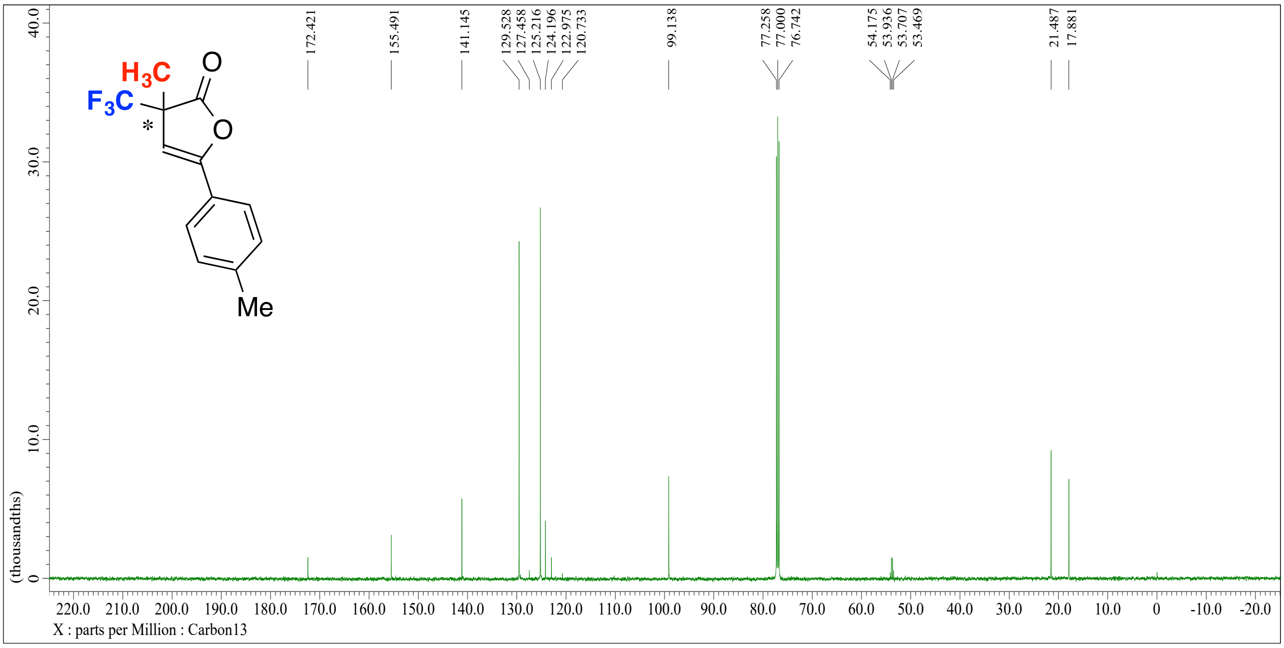

^{19}F NMR (375 MHz, $CDCl_3$) **4b**

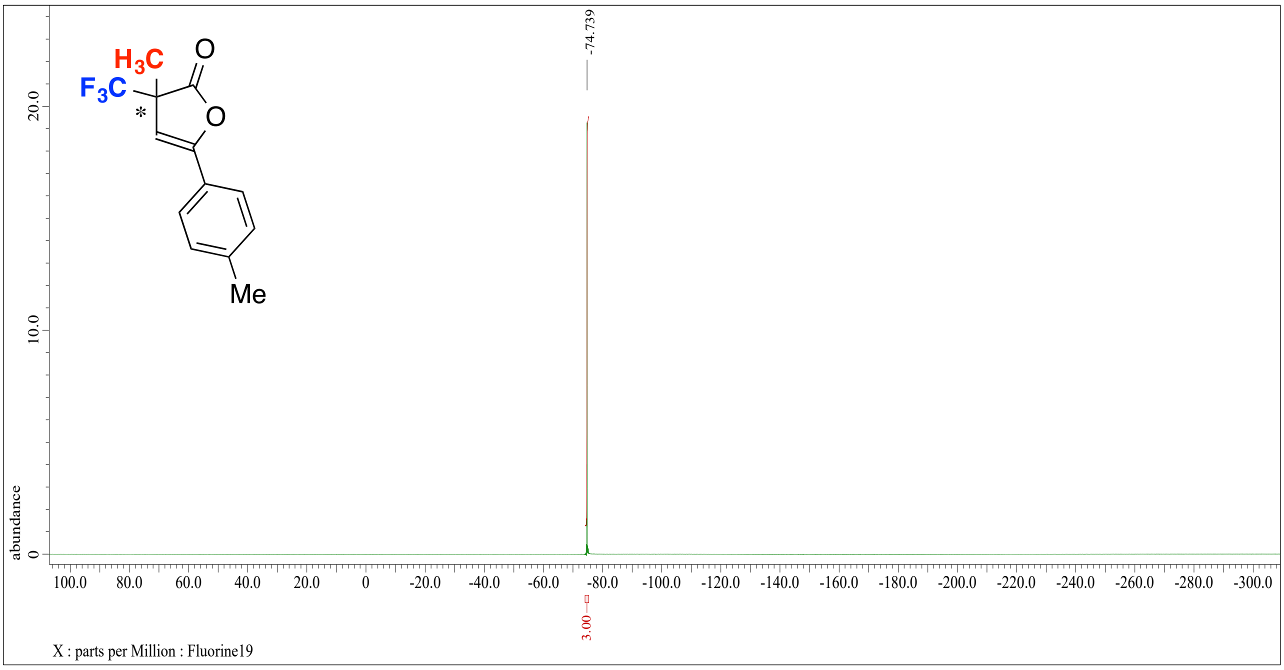

^{1}H NMR (500 MHz, $CDCl_3$) **4c**

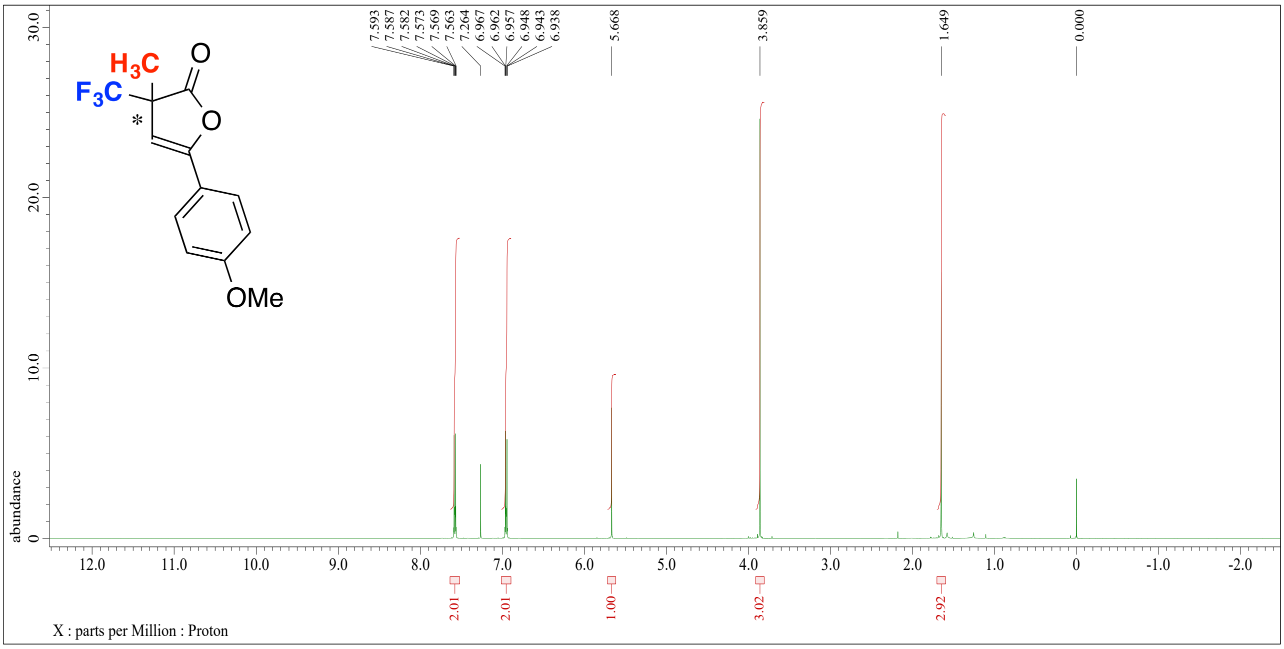

^{13}C NMR (125 MHz, $CDCl_3$) **4c**

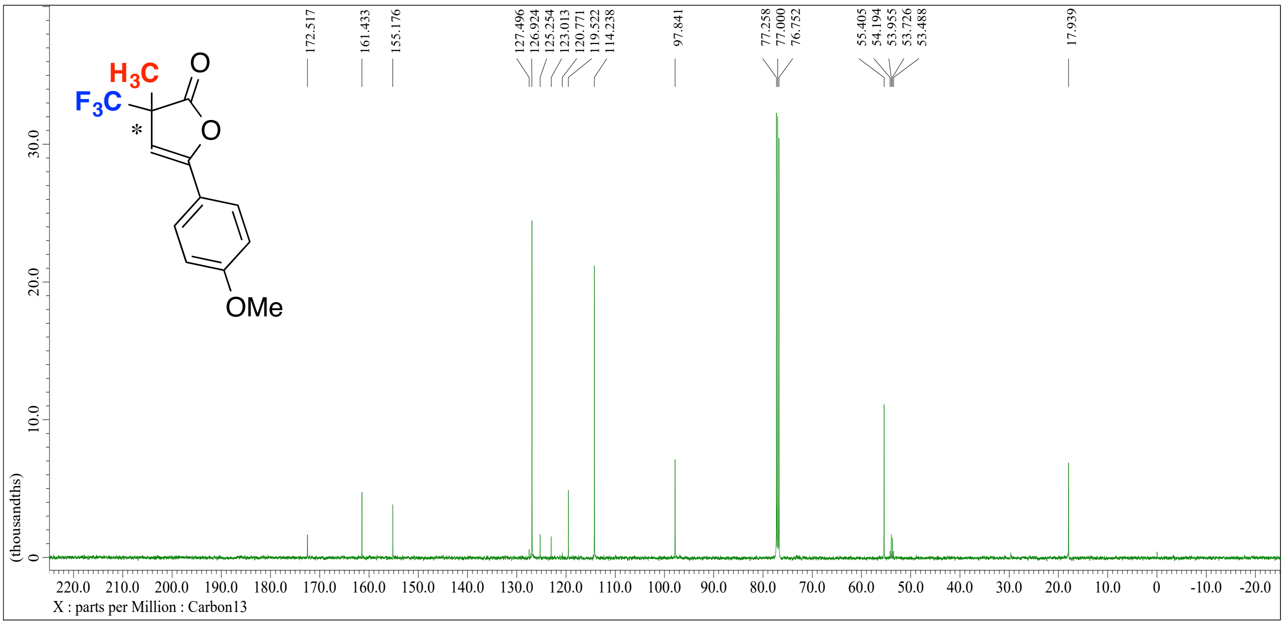

^{19}F NMR (375 MHz, $CDCl_3$) **4c**

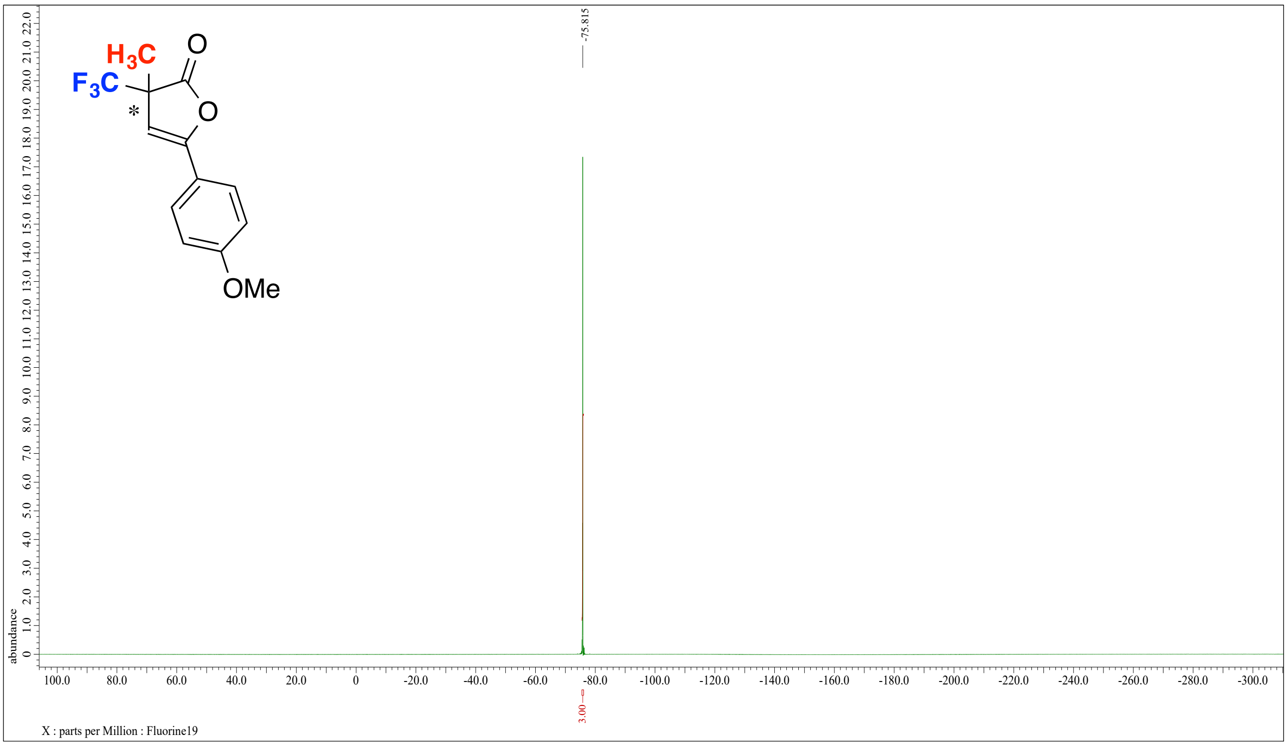

^{1}H NMR (500 MHz, $CDCl_3$) **4d**

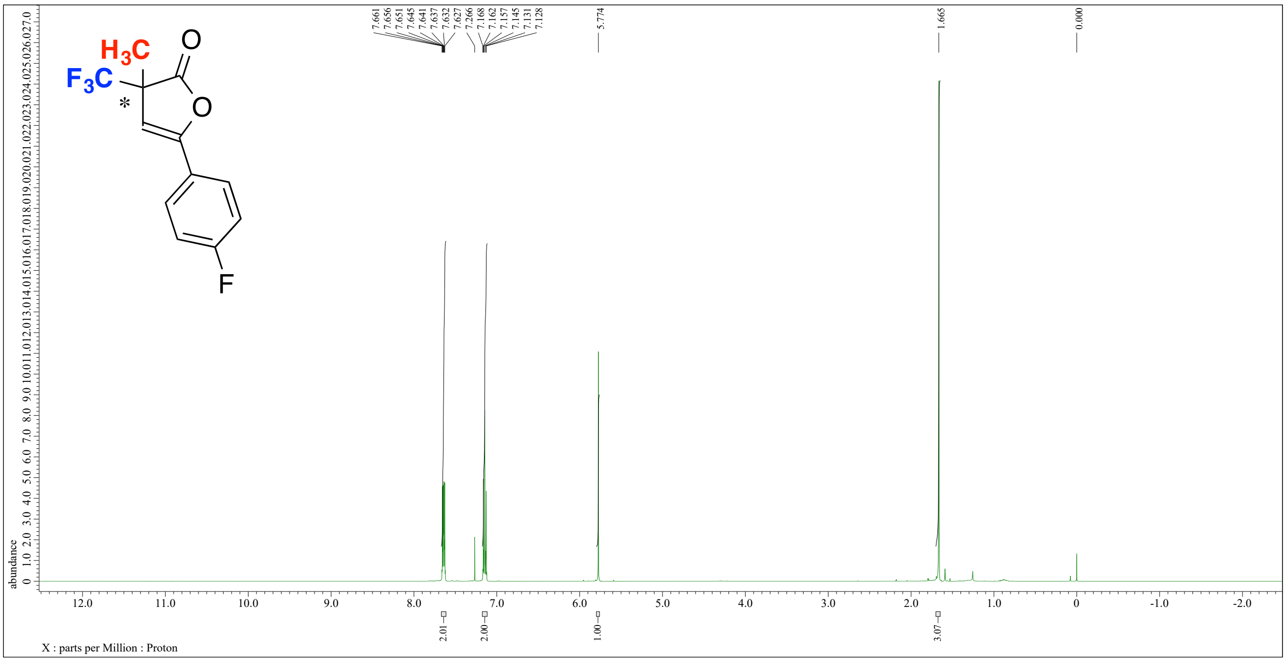

^{13}C NMR (125 MHz, $CDCl_3$) **4d**

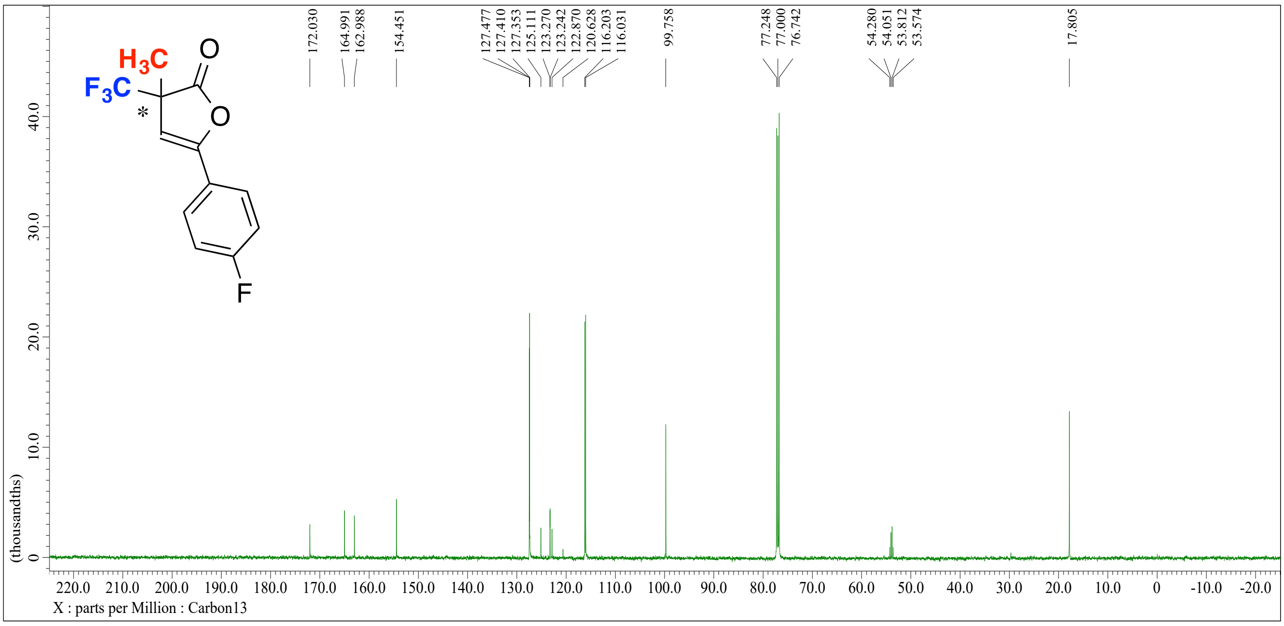

^{19}F NMR (375 MHz, $CDCl_3$) **4d**

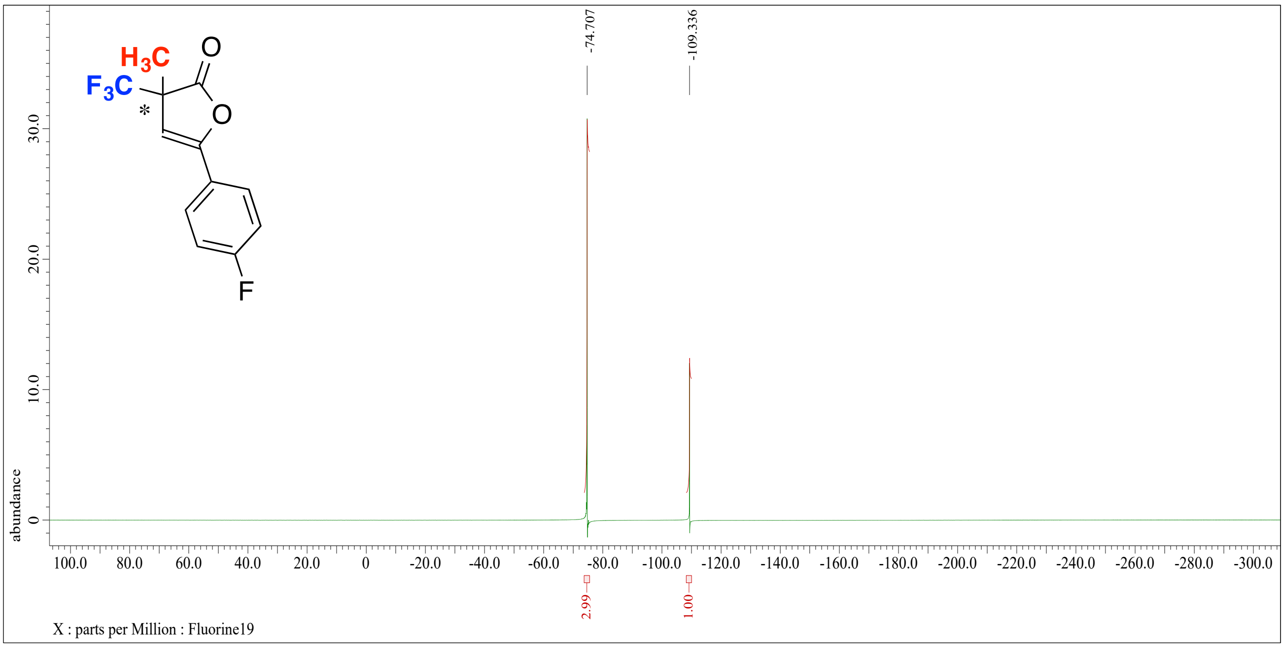

^{1}H NMR (500 MHz, $CDCl_3$) **4e**

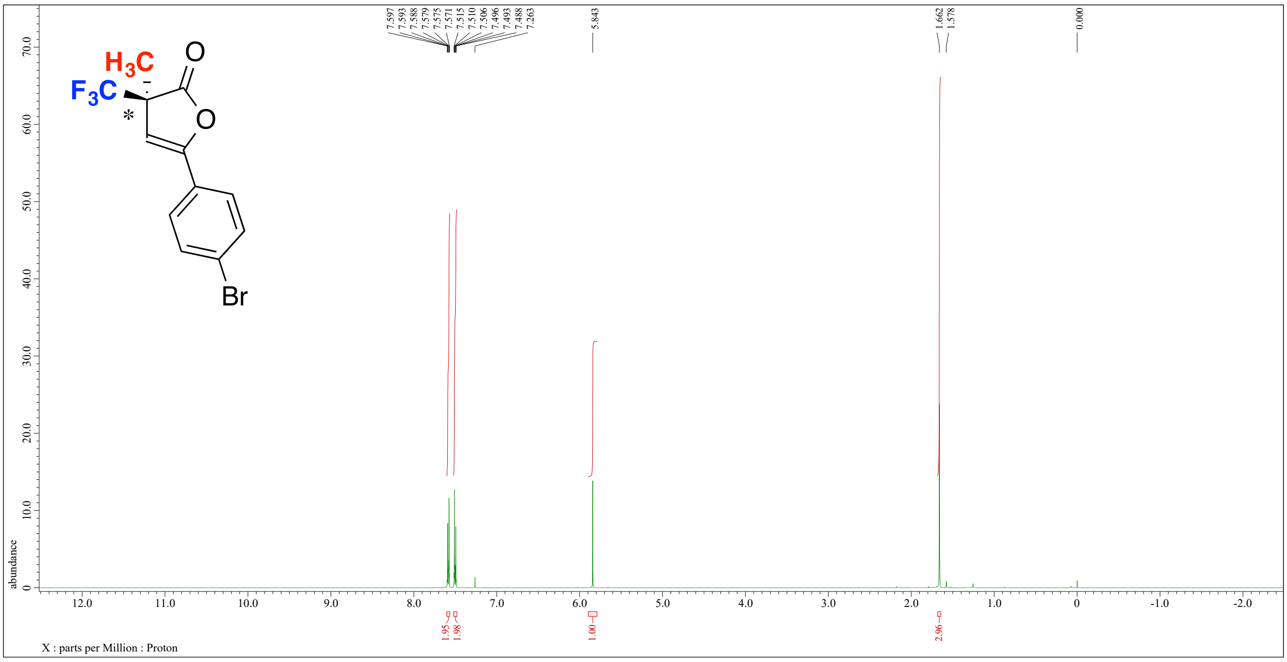

^{13}C NMR (125 MHz, $CDCl_3$) **4e**

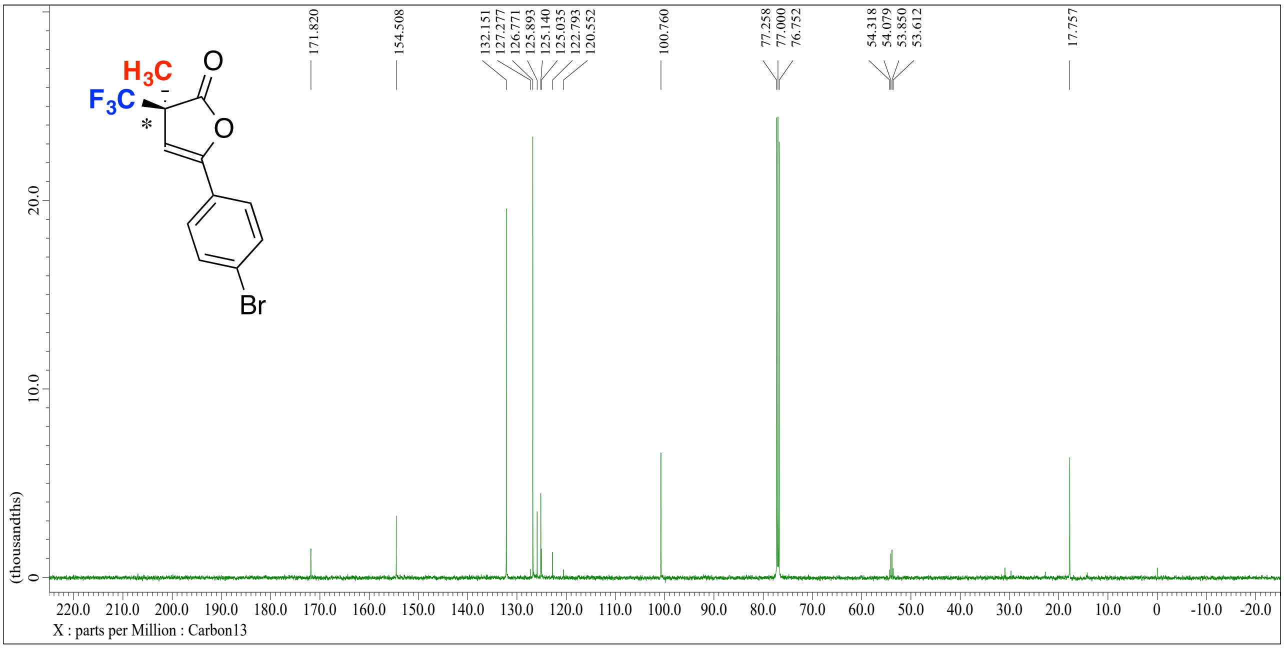

^{19}F NMR (375 MHz, $CDCl_3$) **4e**

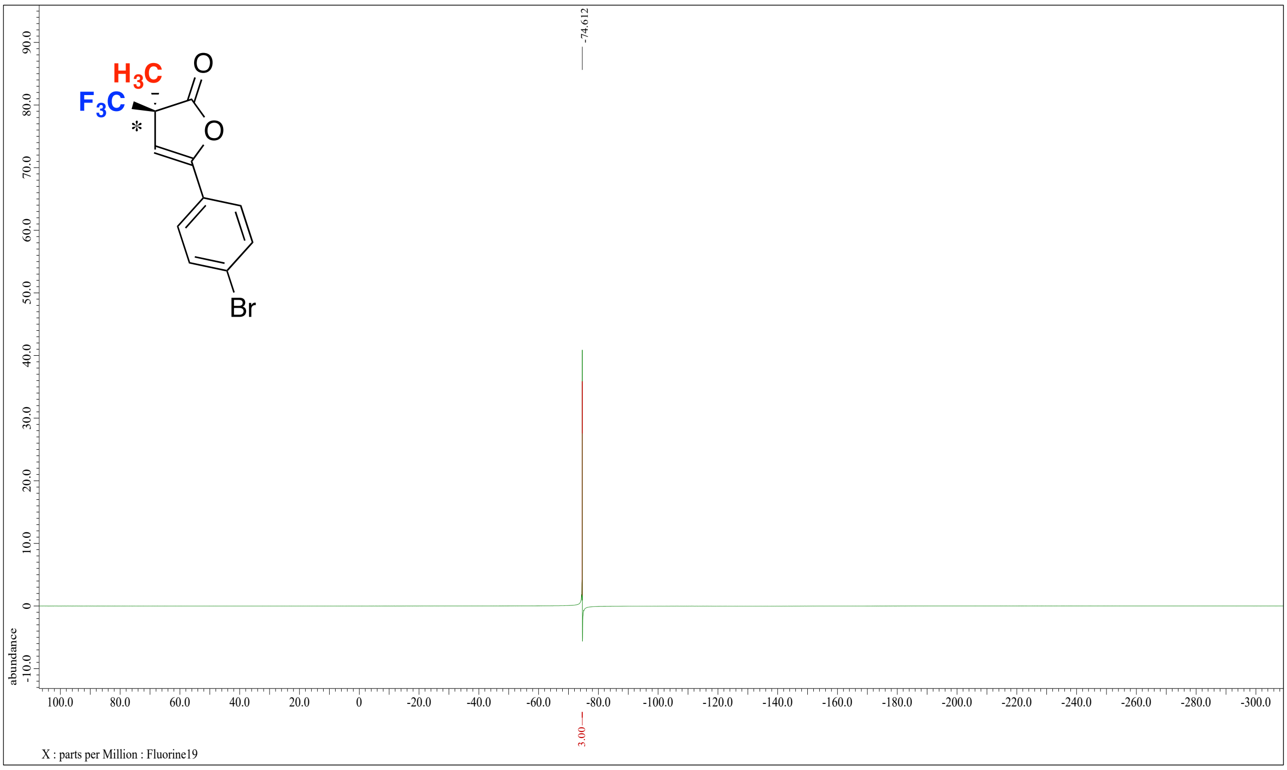

^{1}H NMR (400 MHz, $CDCl_3$) **4f**

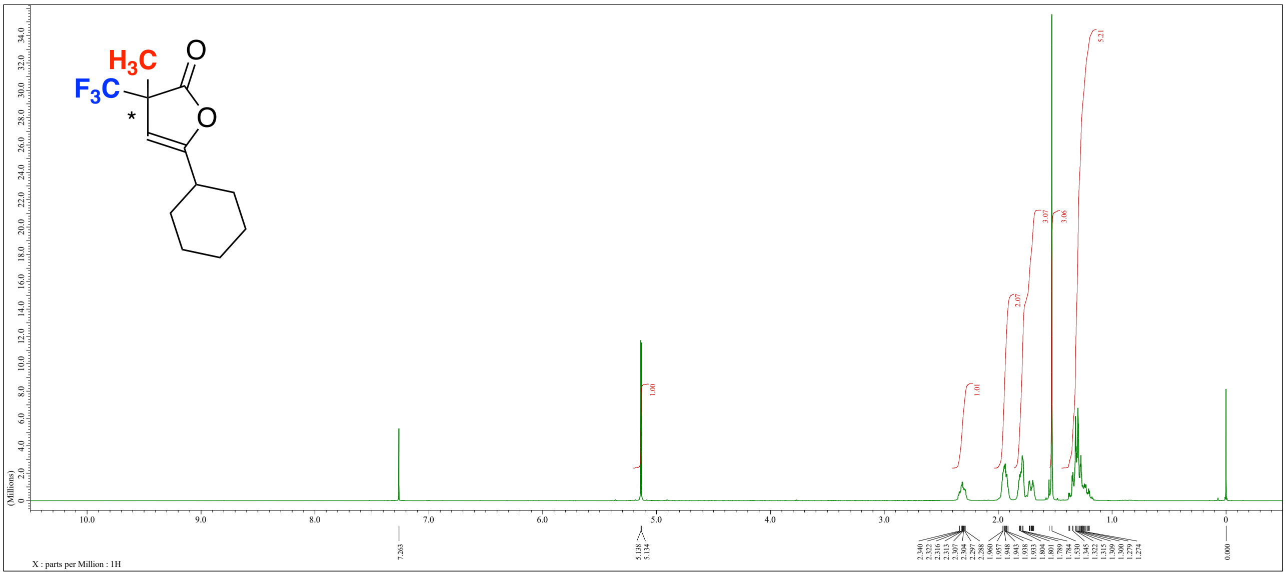

^{13}C NMR (100 MHz, $CDCl_3$) **4f**

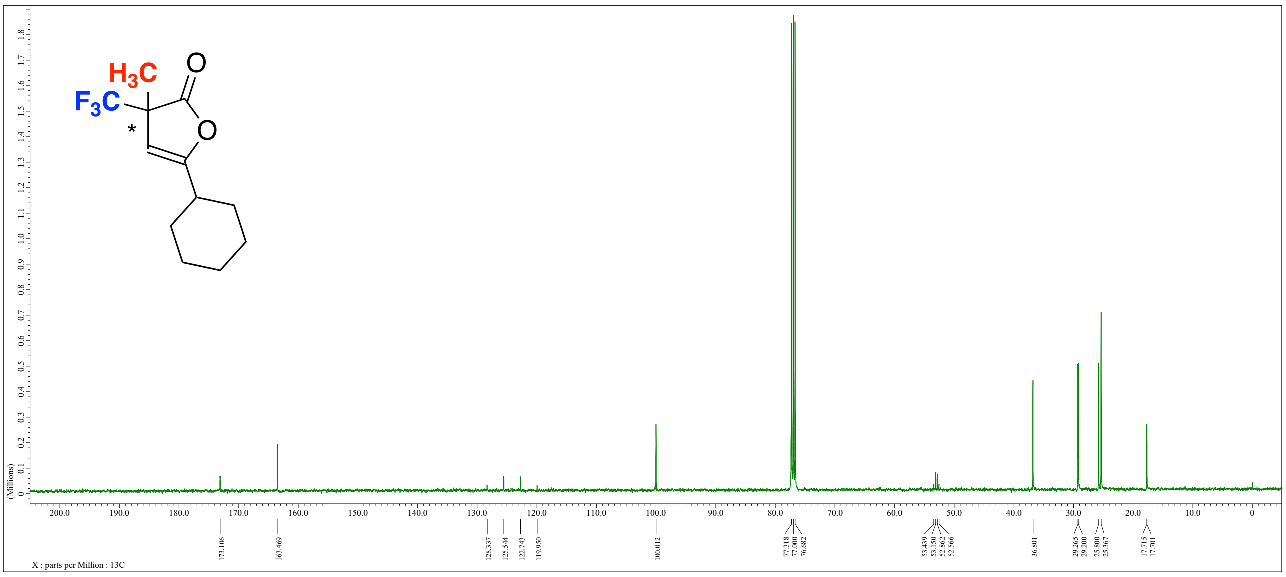

^{19}F NMR (375 MHz, $CDCl_3$) **4f**

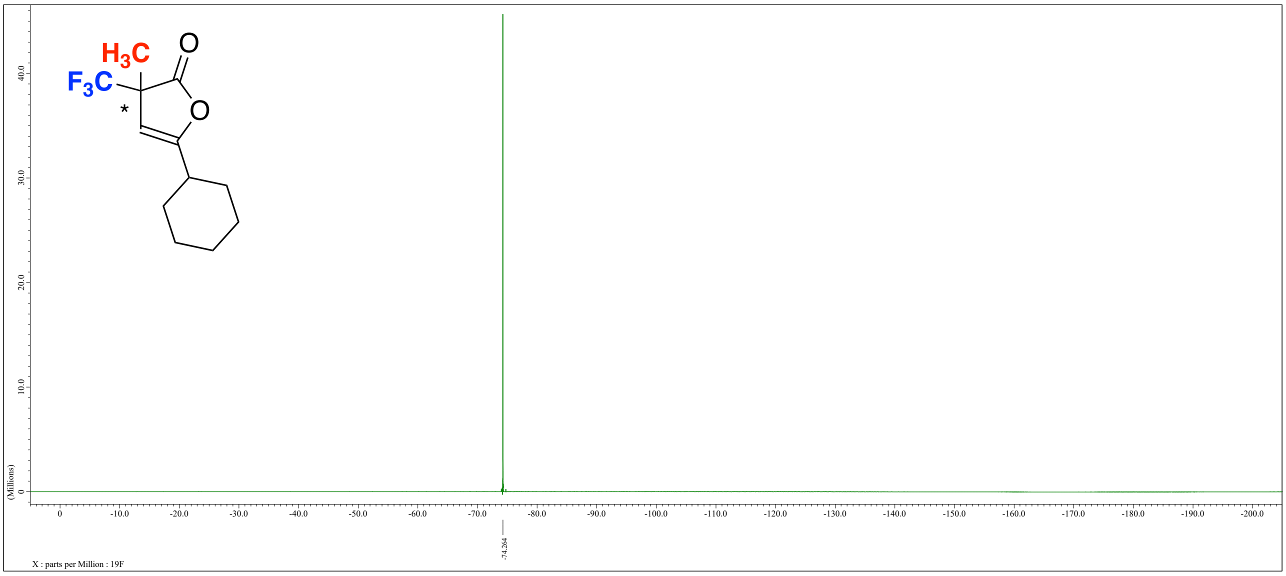

^{1}H NMR (400 MHz, $CDCl_3$) **4h**

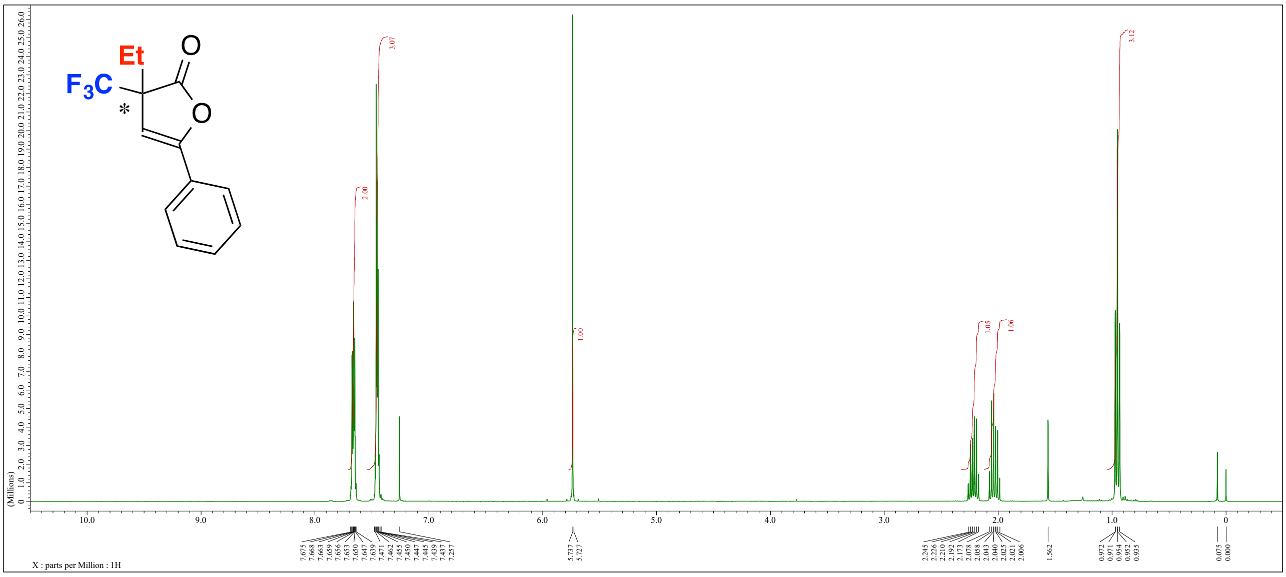

^{13}C NMR (100 MHz, $CDCl_3$) **4h**

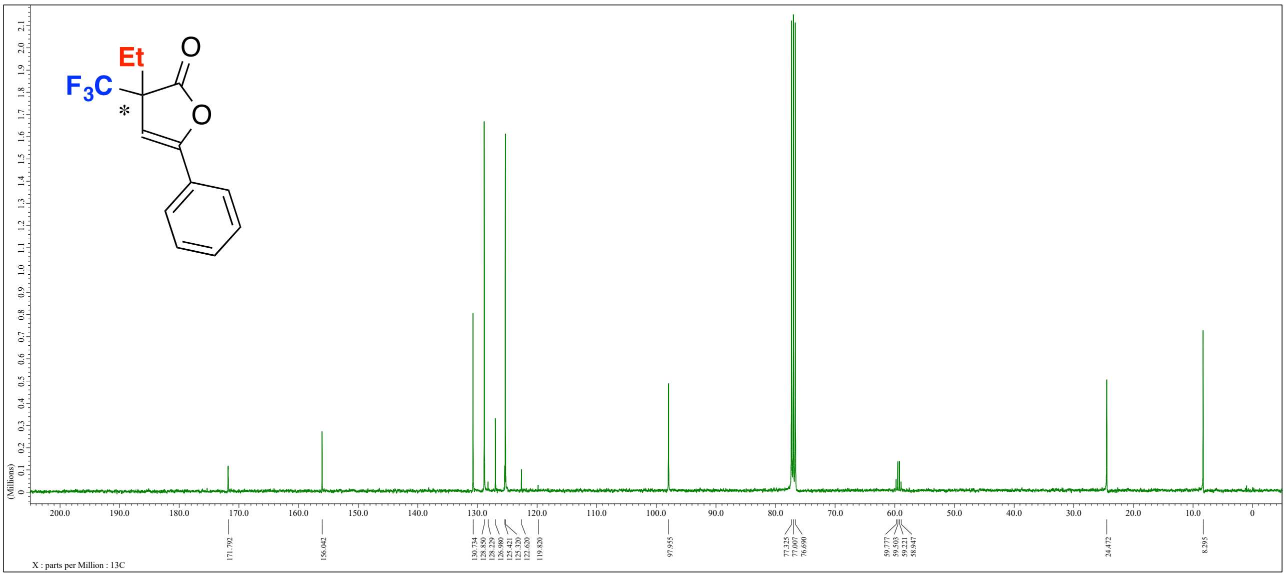

^{19}F NMR (375 MHz, $CDCl_3$) **4h**

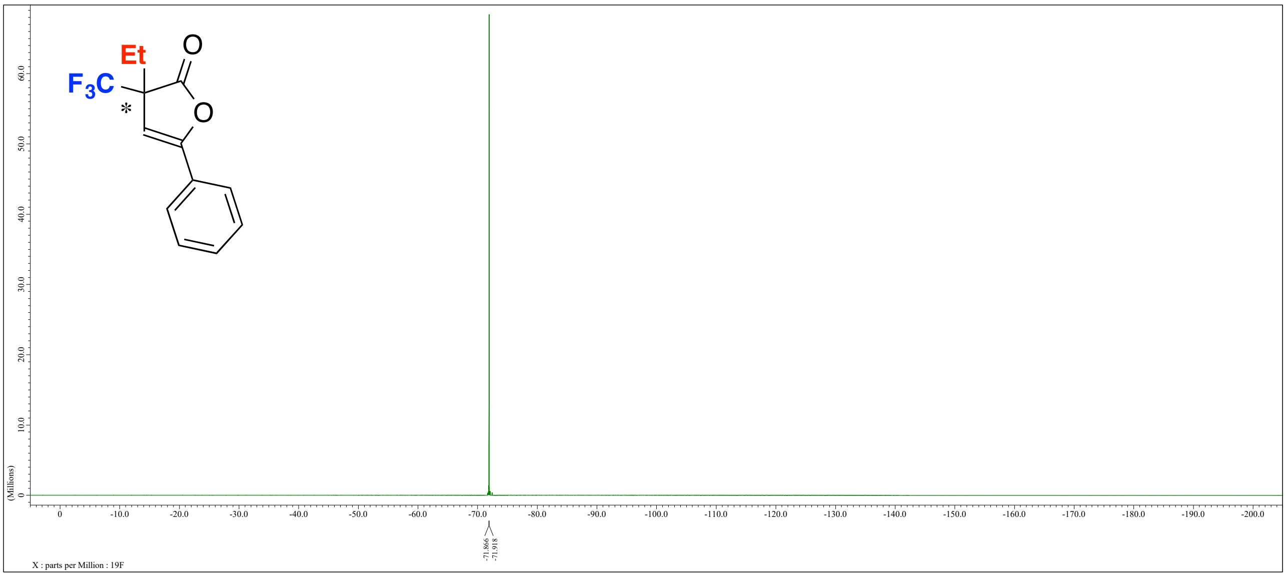

^{1}H NMR (400 MHz, $CDCl_3$) **5a**

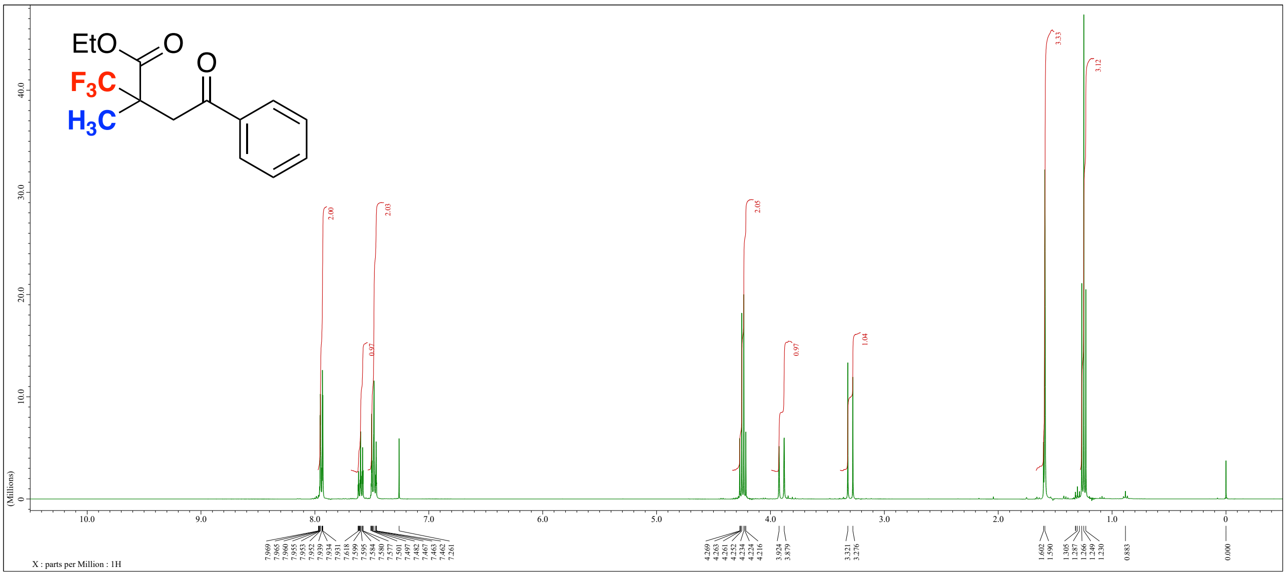

^{13}C NMR (100 MHz, $CDCl_3$) **5a**

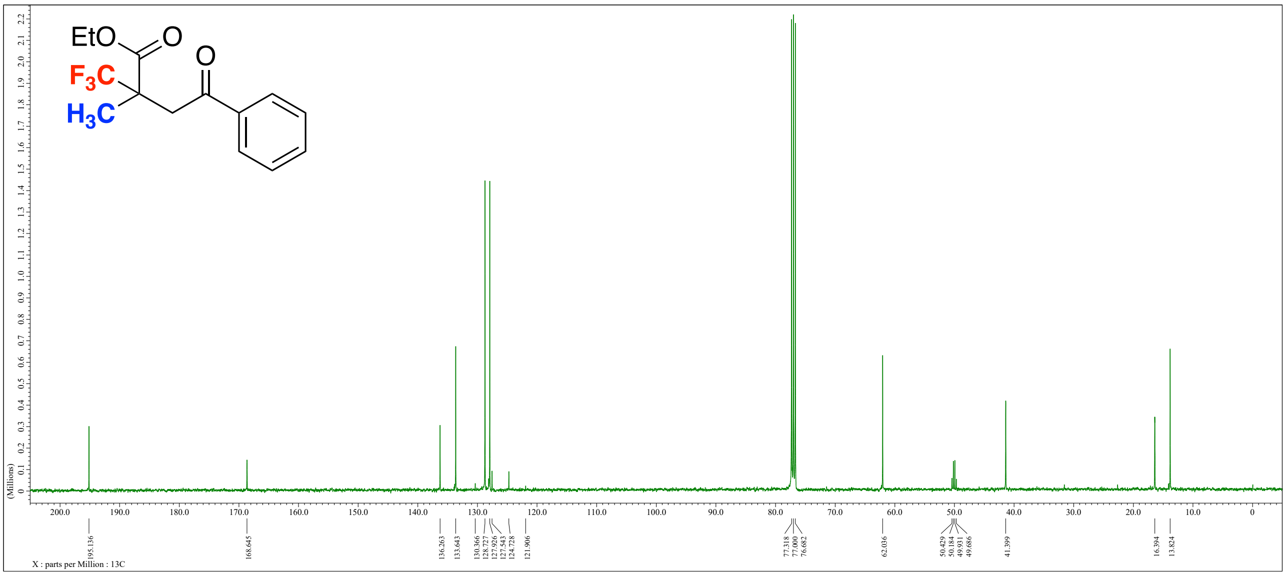

^{19}F NMR (375 MHz, $CDCl_3$) **5a**

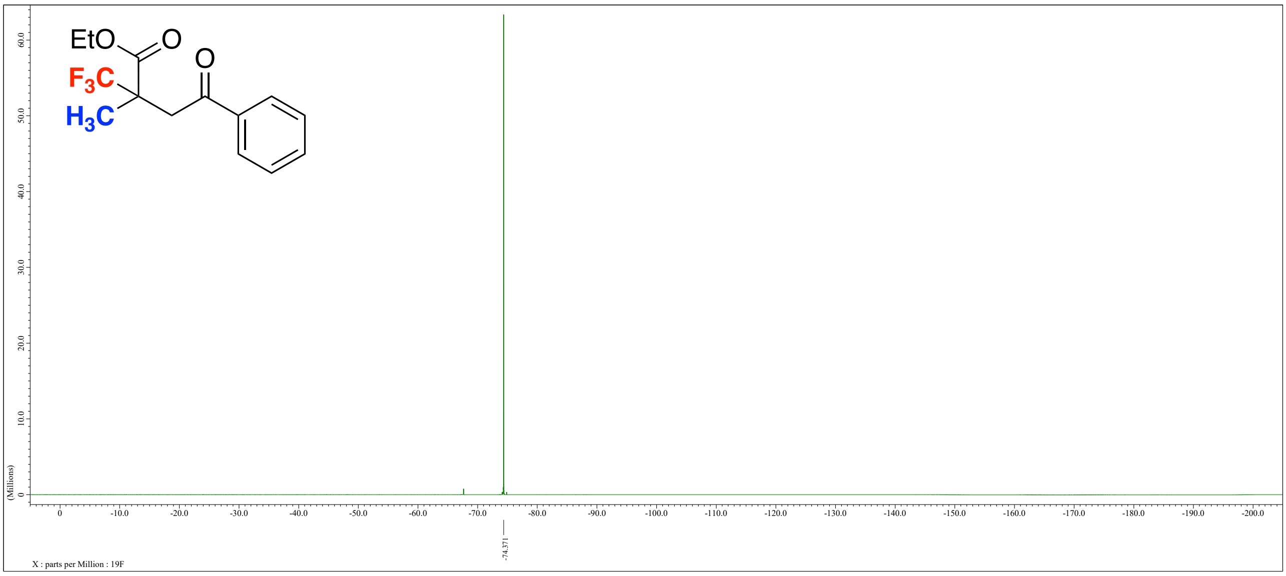

^{1}H NMR (400 MHz, $CDCl_3$) **5g**

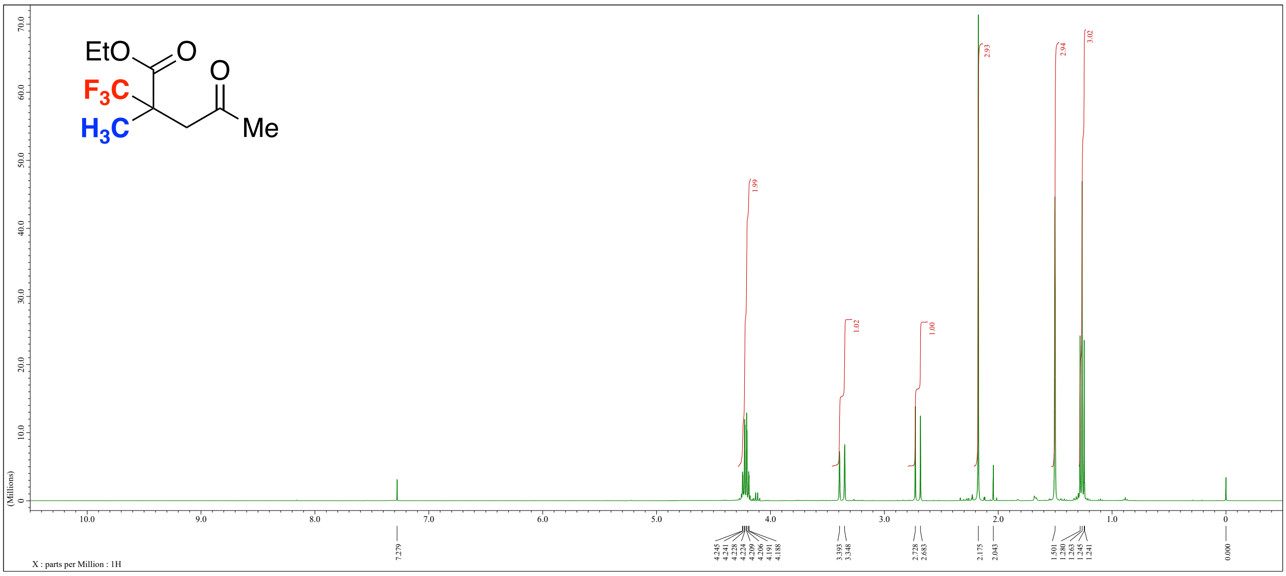

^{13}C NMR (100 MHz, $CDCl_3$) **5g**

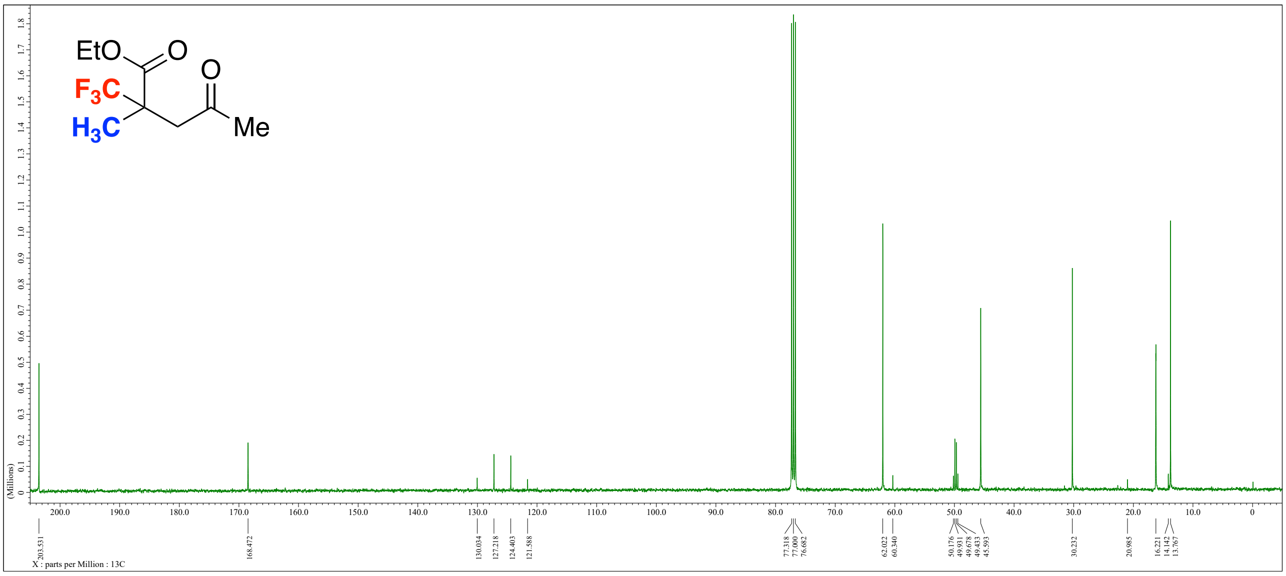

^{19}F NMR (375 MHz, $CDCl_3$) **5g**

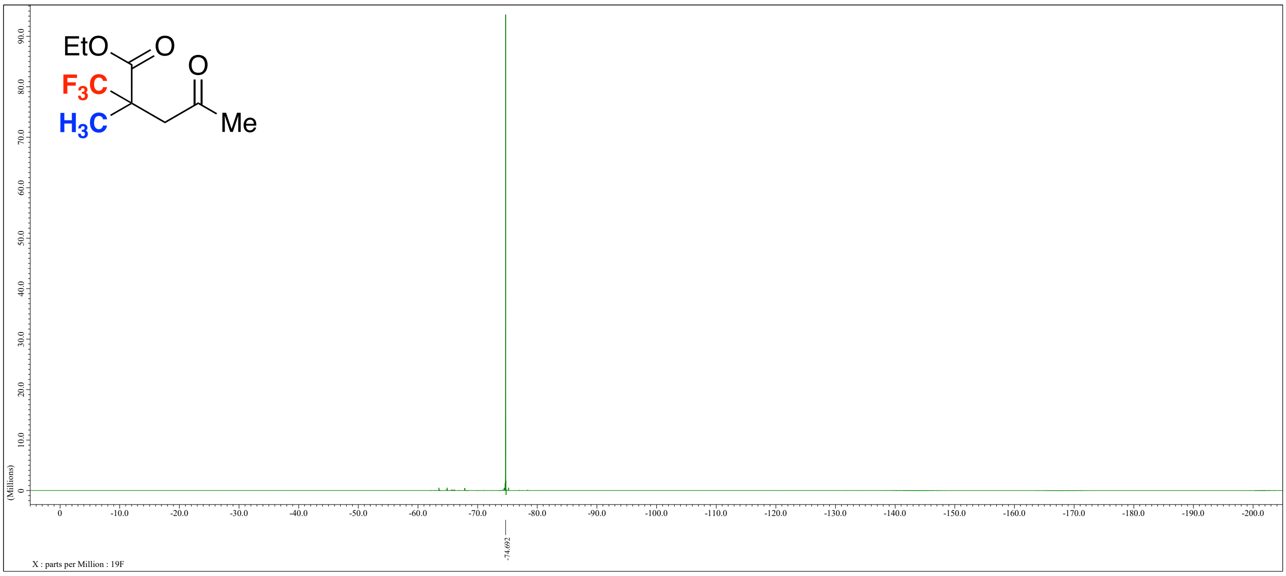

Supplement: Supplementary file 1 — ol4c01691_si_001.pdf [file ol4c01691_si_001.pdf]
